# Photoswitchable fatty acids enable optical control of TRPV1

**DOI:** 10.1038/ncomms8118

**Published:** 2015-05-22

**Authors:** James Allen Frank, Mirko Moroni, Rabih Moshourab, Martin Sumser, Gary R. Lewin, Dirk Trauner

**Affiliations:** 1 Department of Chemistry and Center for Integrated Protein Science, Ludwig Maximilians University Munich, Butenandtstrasse 5–13, Munich 81377, Germany; 2 Molecular Physiology of Somatic Sensation, Max Delbrück Center for Molecular Medicine, Berlin 13125, Germany; 3 Department of Anesthesiology, Campus Charité Mitte und Virchow Klinikum, Charité Universitätsmedizin Berlin, Augustburgerplatz 1, Berlin 13353, Germany

## Abstract

Fatty acids (FAs) are not only essential components of cellular energy storage and structure, but play crucial roles in signalling. Here we present a toolkit of photoswitchable FA analogues (FAAzos) that incorporate an azobenzene photoswitch along the FA chain. By modifying the FAAzos to resemble capsaicin, we prepare a series of photolipids targeting the Vanilloid Receptor 1 (TRPV1), a non-selective cation channel known for its role in nociception. Several azo-capsaicin derivatives (AzCAs) emerge as photoswitchable agonists of TRPV1 that are relatively inactive in the dark and become active on irradiation with ultraviolet-A light. This effect can be rapidly reversed by irradiation with blue light and permits the robust optical control of dorsal root ganglion neurons and C-fibre nociceptors with precision timing and kinetics not available with any other technique. More generally, we expect that photolipids will find many applications in controlling biological pathways that rely on protein–lipid interactions.

## Abstract (web summary)

Fatty acids are ancient lipids with numerous functions, from metabolic processes as a source of energy to structural and signalling roles within cell membranes. Here, the authors present azobenzene-modified fatty acids and their application as photoswitchable agonists of the Vanilloid Receptor 1.

## Graphical abstract

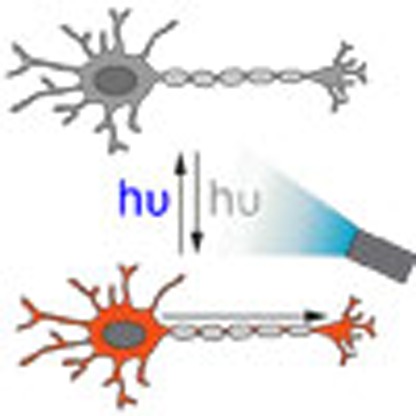

Lipids serve not only as sources of energy and integral components of membranes but are also involved in cellular communication through participation in a variety of signalling cascades and the modulation of transmembrane proteins^1^. Over the past several decades, interest in lipid chemistry has been overshadowed by advancements in proteomic and genomic technologies. However, recent developments in lipid research, including analysis of the lipidome^2^, have shed new light on the roles of these molecules at all levels of biology. Many lipids consist of fatty acids (FAs). These ancient molecular building blocks typically feature a long linear carbon chain (up to 28 carbons)^3^ that often contains one or several *cis*- double bonds.

The Vanilloid Receptor 1 (TRPV1) is the most studied of the transient receptor potential ion channels^4^,^5^. This family of non-selective cation channels is renowned for its ability to respond to a wide variety of chemical and physical inputs^6^,^7^. TRPV1 is involved in the regulation of body temperature^8^ and the transduction of painful stimuli from the periphery towards the central nervous system^9^. It is expressed in sub-populations of sensory nerve fibres within the dorsal root and trigeminal ganglia^10^, where it responds to temperatures greater than 43 °C^11^, protons^12^, as well as environmental toxins and poisons^13^,^14^. Importantly, TRPV1 is modulated by a plethora of FA amides, including the endogenous arachidonic acid derivatives anandamide^15^ and *N*-arachidonoyl dopamine^16^. Its most famous exogenous agonist is the vanilloid capsaicin (CAP), the pungent component of chilli peppers^11^. Synthetic TRPV1 agonists include olvanil^17^ and arvanil^18^, which are FA-derived vanilloids developed as non-pungent CAP analogues.

TRPV1 is not only involved in responses to noxious stimuli, but is also believed to initiate the neurogenic inflammatory response^19^, which has made it an attractive target for novel analgesics^11^. However, these attempts have proven more challenging than anticipated, as TRPV1 is involved in a variety of other biological pathways that can lead to unwanted side effects. An agonist or antagonist that could be applied globally but activated only locally could offer a solution to this problem. In addition to this, such a tool would be highly valuable for untangling the complex interactions that TRPV1 has with other proteins, such as the serotonin (5-HT), bradykinin (BK) and recently GABA_B_ receptors^5^,^20^,^21^.

Precision control can be achieved through photoswitchable small molecules that act as transducers between a light stimulus and protein function^22^,^23^,^24^. In 2013, our group was the first to place TRPV1 under reversible optical control when we developed a series of photoswitchable antagonists which could optically control TRPV1 in the presence of CAP to activate the channel^25^. Based on the structure of CAP and other TRPV1 agonists, we saw the opportunity to develop a photoswitchable TRPV1 agonist which would permit optical control over the ion channel without the use of a second factor.

In this study, we present a toolkit of photolipids that allow us to place lipid-modulated biological targets, such as TRPV1, under the precise spatial and temporal control of light. We show that AzCA4 permits optical control over TRPV1 in complex neural systems with a higher degree of spatiotemporal precision than is currently possible via other methods. More generally, this work represents the first example of the fusion of photopharmacology with lipid signalling and consequently sets the groundwork for future research in this field.

## Results

### Photolipid syntheses

**Figure 1:**
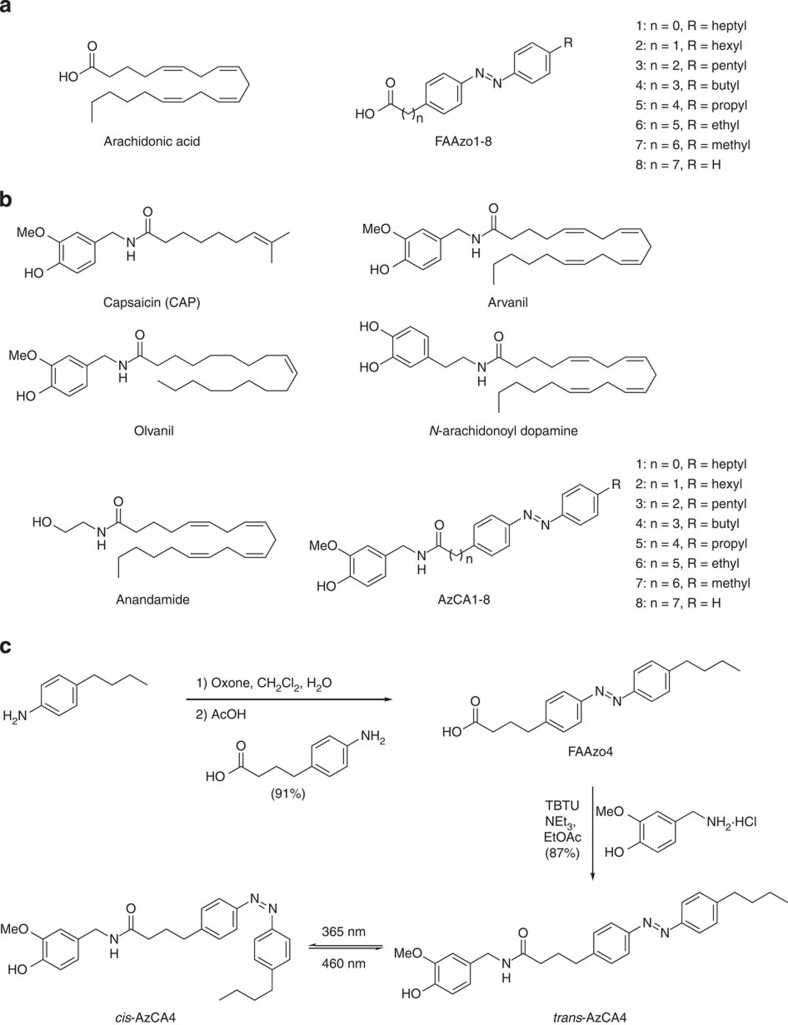
Photolipids for the optical control of TRPV1. (**a**) Chemical structures of arachidonic acid and FAAzo1–8. (**b**) Chemical structures of TRPV1 agonists capsaicin (CAP), arvanil, olvanil, *N*-arachidonoyl dopamine and anandamide alongside photoswitchable vanilloids, AzCA1–8. (**c**) Chemical synthesis of AzCA4. Isomerization between *cis*- and *trans*-AzCA4 could be induced by irradiation with *λ*=365 nm (*trans* to *cis*) and *λ*=460 nm (*cis* to *trans*), respectively.

In an effort to mimic FAs with a chain length of 18 carbons, we prepared a series of 8 photoswitchable FA derivatives, FAAzo1–8. Each of these compounds contained an azobenzene photoswitch, which allowed for controllable *cis*/*trans*-isomerization along the length of the chain (Fig. 1a). FAAzo1–8 were prepared in between two and six steps in moderate yield (Supplementary Fig. 1)^26^. In their dark-adapted state, the FAAzos existed predominantly in the *trans*-configuration. Ultraviolet–visible spectroscopy showed that isomerization from *trans*- to *cis*- could be achieved by irradiation at *λ*=365 nm and this process could be reversed by *λ*=460 nm light.

Supported by structure–activity relationships and recent structural data^27^, we envisioned a unique opportunity to create a series of photoswitchable vanilloids for the optical control of TRPV1. Our approach combined the vanilloid head group of CAP with the FAAzos, which would act as a photoswitchable tail mimicking that of *N*-arachidonoyl dopamine, anandamide, olvanil or arvanil (Fig. 1b). The preparation of these compounds (Fig. 1c and Supplementary Fig. 2) required only a peptide coupling between vanillamine and the appropriate FAAzo to afford eight photoswitchable vanilloids, AzCA1–8, in good yields (Fig. 1b). Photoswitching of AzCA1–8 was also achieved using *λ*=365/460 nm and they showed similar photoswitching properties when compared with the FAAzos (Supplementary Fig. 3). As such, both FAAzos and AzCAs could be classified as ‘regular' azobenzenes that require ultraviolet-A light for isomerization to their thermally unstable *cis*-form.

### Optical control over TRPV1 in HEK293T cells

**Figure 2:**
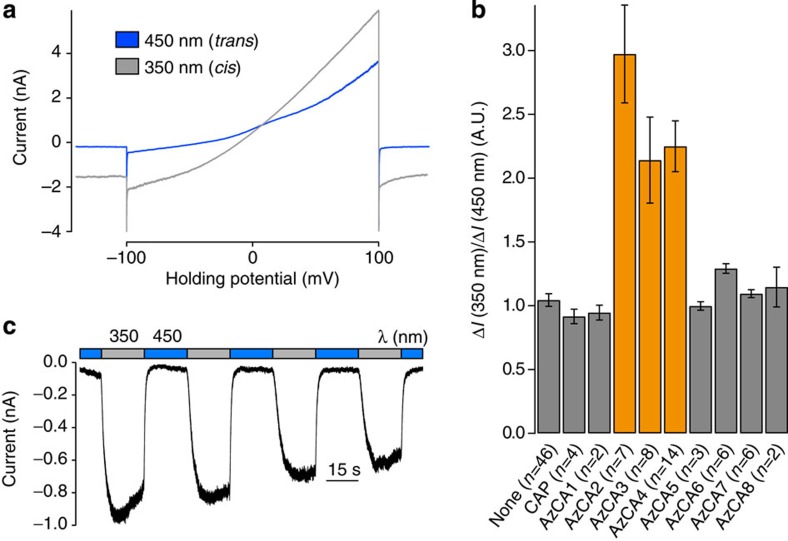
AzCAs permit optical control of TRPV1 in HEK293T cells. HEK293T cells expressing TRPV1–YFP were observed using whole-cell patch clamp electrophysiology after the application of an AzCA derivative (1 μM). Error bars were calculated as s.e.m. (**a**) Voltage ramps were applied under both *λ*=350 nm and *λ*=450 nm irradiation. Larger currents were observed under *λ*=350 nm irradiation (AzCA4). (**b**) AzCA2, AzCA3 and AzCA4 emerged as photoswitchable TRPV1 agonists. (**c**) When voltage clamped, photoswitching could be repeated over many cycles (AzCA3).

We evaluated the photopharmacology of AzCA1–8 using whole-cell electrophysiology in human embryonic kidney (HEK) 293T cells transiently expressing the yellow fluorescent protein (YFP)-tagged ion channel (TRPV1-YFP)^28^. Each compound (1 μM) was continuously applied while alternating between irradiation at *λ*=350 nm and at *λ*=450 nm, until a steady state of activity/desensitization was achieved on photoswitching. Their relative light-dependant activities were then assessed by performing voltage ramps (−100 to +100 mV over 5 s) under irradiation at both wavelengths (Fig. 2a and Supplementary Fig. 4a). Among the eight derivatives tested, three compounds—AzCA2, AzCA3 and AzCA4 (at 1 μM)—showed the most profound TRPV1 photoswitching effect (Fig. 2b and Supplementary Fig. 4b). For all three derivatives, larger currents were observed under irradiation at *λ*=350 nm, indicating that these compounds had higher efficacies towards TRPV1 in their *cis*-configuration. At higher concentrations (>300 nM, bath application), smaller cellular currents were observed on application of the AzCAs in their dark-adapted states. This showed that in both configurations, the AzCAs were TRPV1 agonists; however, in all cases a larger current was observed under *λ*=350 nm irradiation. Working at an optimized concentration (1 μM by puff pipette), the AzCAs could be applied to cells in the dark without any observable effect and this allowed immediate TRPV1 activation on irradiation with ultraviolet-A light (Supplementary Fig. 5). Photoswitching could be repeated over multiple cycles and only minor channel desensitization was observed (Fig. 2c).

The washout of AzCA4 with buffer was very slow and photoswitching currents persisted for minutes under constant perfusion. However, application of capsazepine (5 μM), a TRPV1 antagonist known to bind competitively against CAP, was capable of displacing AzCA4 and rapidly abolished inward currents on ultraviolet stimulation (Supplementary Fig. 6)^29^,^30^. In control experiments, no light-induced activity was observed in cells lacking TRPV1, before the application of an AzCA derivative (Supplementary Fig. 7a) or after the application of CAP (1 μM) (Supplementary Fig. 7b). The application of FAAzo2, FAAzo3 and FAAzo4 caused no effect (5 μM, *n*⩾3 for each) in TRPV1-responding cells.

**Figure 3:**
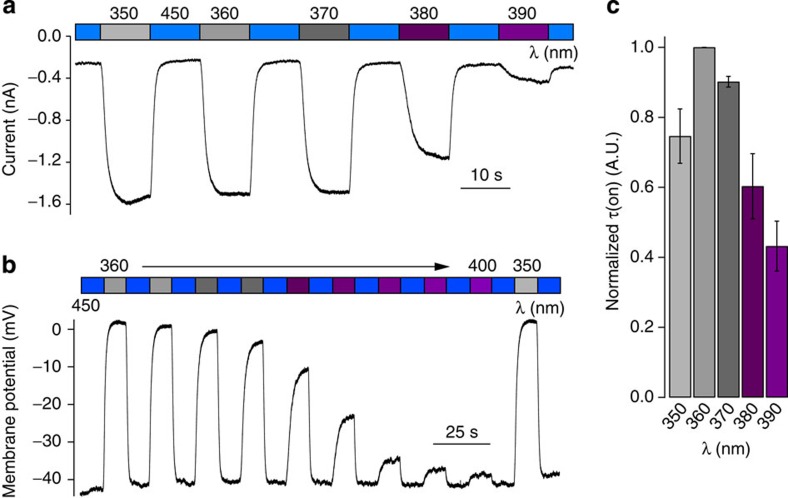
TRPV1 activation can be precisely controlled with light. Cellular currents were observed using whole-cell patch clamp electrophysiology in HEK293T cells expressing TRPV1–YFP after the application of an AzCA derivative (1 μM). Error bars were calculated as s.e.m. (**a**) The current magnitude could be controlled by adjusting the ON wavelength between *λ*=350 and 390 nm (AzCA3). (**b**) When current clamped, the membrane potential could be controlled by adjusting the ON wavelength between *λ*=350 and 400 nm (AzCA4). (**c**) The activation rate could be controlled by adjusting the ON wavelength between *λ*=350 and 390 nm (AzCA3, *n*=3).

In voltage clamp experiments, the magnitude of the cellular currents could be controlled by adjusting the ON wavelength between *λ*=350–390 nm. As shown by an action spectrum, the largest currents were observed under *λ*=350 nm and they became smaller towards *λ*=390 nm (Fig. 3a and Supplementary Fig. 8). Current clamp experiments revealed that the cellular membrane potential could be controlled in a similar manner, with *λ*=360 nm yielding the largest depolarization (Fig. 3b). Through exponential curve fitting, we evaluated the effects of irradiation on the TRPV1 activation kinetics. The fastest ON response was observed at *λ*=360 nm and all other *τ*_on_ values were normalized to this *τ*-value for comparison (Fig. 3c). At longer wavelengths, a slower response was observed. Taken together, these results indicate that the AzCAs permit precise, optical control over the activity of TRPV1 that cannot be achieved with other lipophilic agonists, such as CAP, alone. As AzCA4 could be prepared in high yield at low cost, we decided to focus our future investigations on this compound.

### AzCA4 allowed optical control over cultured DRG neurons

Having demonstrated that AzCA4 acts as a photoswitchable TRPV1 agonist that is relatively inactive in the dark, we next evaluated its activity in isolated wild-type (wt) mouse dorsal root ganglia (DRG) neurons using both electrophysiology and intracellular Ca^2+^ imaging.

**Figure 4:**
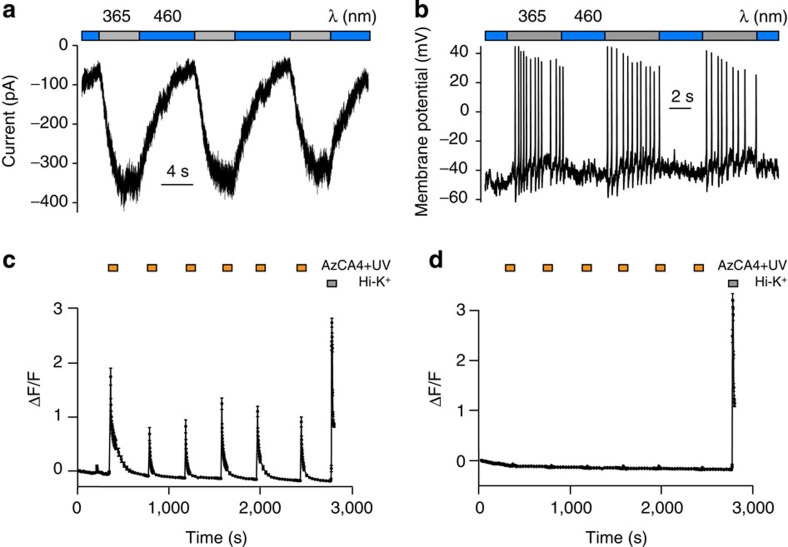
AzCA4 enabled optical control over cultured wt DRG neurons. (**a**) After the bath application of AzCA4 (200 nM), neuronal currents could be modulated by monochromatic irradiation. When clamped at a holding potential of −60 mV, an inward current was observed on irradiation with *λ*=365 nm and this effect could be reversed with irradiation at *λ*=460 nm. (**b**) When clamped at a current of 0 pA and after the application of AzCA4 (200 nM), the neuronal membrane potential could be controlled. AP firing could be induced by irradiation at *λ*=365 nm and halted with *λ*=460 nm. This process could be repeated over many cycles. (**c**) Intracellular Ca^2+^ imaging showed that AzCA4 (100 nM) significantly increased the level of intracellular Ca^2+^ in TRPV1-positive neurons only after irradiation at *λ*=365 nm (5 s). (**d**) Cells that did not respond to AzCA4 showed no increase in intracellular Ca^2+^ after the bath application of AzCA4 and an ultraviolet pulse (6 pulses, 100 nM, 5 s at *λ*=365 nm). These cells still responded to a Hi-K^+^ solution (100 mM), suggesting that they did not possess TRPV1. Error bars were calculated as s.e.m.

Whole-cell patch clamp electrophysiology showed that AzCA4 (200 nM) enabled reversible optical control over DRG neuronal activity. Switching between *λ*=365 nm and *λ*=460 nm in the voltage clamp configuration at a holding potential of −60 mV resulted in reversible control over the transmembrane currents (Fig. 4a). Accordingly, the membrane potential and excitability of DRG neurons could be optically controlled as well. Action potential (AP) firing was reversibly switched ON and OFF by irradiation with *λ*=365 nm and *λ*=460 nm, respectively (Fig. 4b).

Bath application of AzCA4 (100 nM) and irradiation at *λ*=365 nm (5 s) caused an increase in intracellular Ca^2+^ concentration in ∼30% of DRG neurons cultured from 2- to 3-week-old mice (Fig. 4c and Supplementary Fig. 9). The remaining 70% of the DRG neurons did not respond to AzCA4 but still responded to a high potassium (Hi-K^+^) solution (100 mM) (Fig. 4d). The percentage of AzCA4 responding neurons was consistent with the percentage of TRPV1 containing DRG neurons at this stage of mouse development (33.6±1.3% at P15, 30.5±3% at P22)^31^.

### AzCA4 is selective for TRPV1-expressing neurons in the DRG

TRPV1 knockout mutant mice (*Trpv1*^*−/−*^) have proven to be an excellent negative control to study the functional aspects of TRPV1 and the role of CAP-sensitive afferent neurons in the mammalian nervous system^32^. We used this mouse line to assay the selectivity of AzCA4 in DRG neurons. As expected, Ca^2+^ imaging in *Trpv1*^*−/−*^ mouse DRG neurons showed no neural activity in response to AzCA4 (300 nM) or CAP (300 nM). Control experiments showed that these neurons still responded to menthol (100 μM, data not shown), ATP (20 μM) and Hi-K^+^ (100 mM) solutions (Supplementary Fig. 10). In combination, these results indicate that AzCA4 acted solely on TRPV1-positive neurons in the DRG.

### Optical control of C-fibre nociceptors

**Figure 5:**
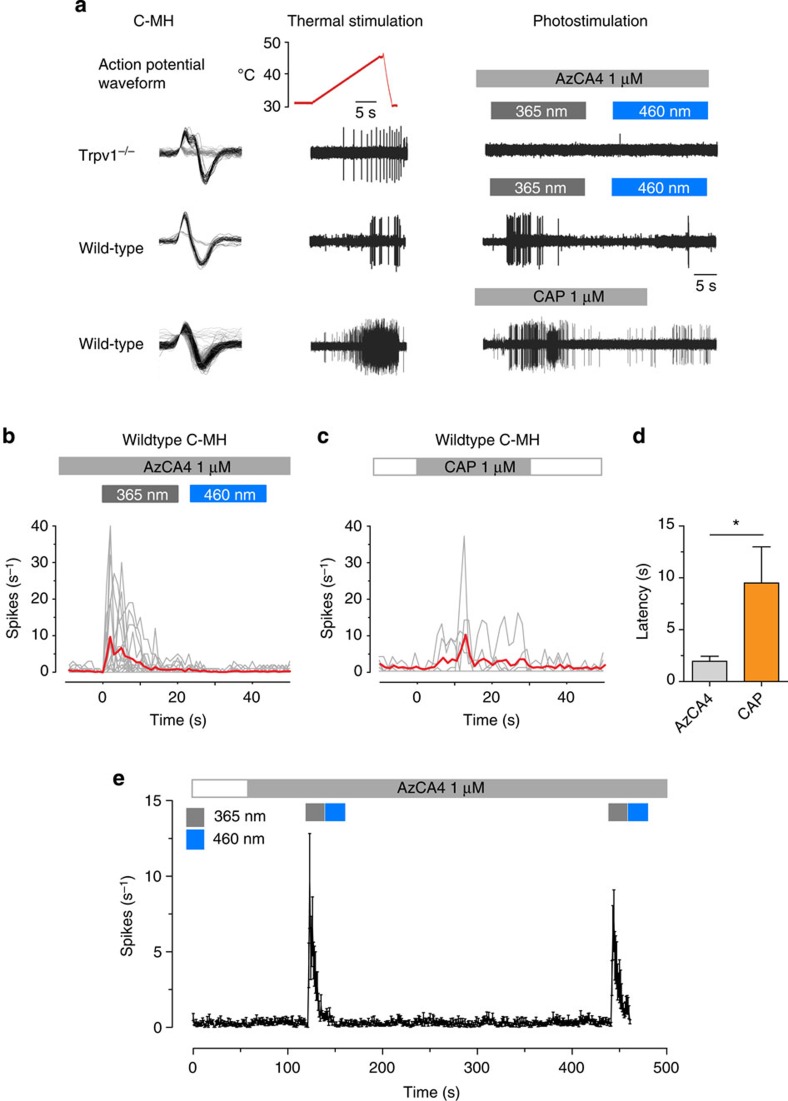
Optical activation of TRPV1 in C-fibre nociceptors. (**a**) A typical sample trace showing the activation of a heat-sensitive C-fibre nociceptor (C-MH) by optical switching of AzCA4 (1 μM) with *λ*=365 nm light and CAP (1 μM) application in wt mice. No photostimulation occurred in *Trpv1*^*−/−*^ mice. (**b**,**c**) The average AP spiking rate (red) and the spiking rate for individual C-MHs (grey) in response to photostimulation in the presence of AzCA4 (1 μM, *n*=19), and activation by CAP (1 μM, *n*=6). (**d**) The latencies to the occurrence of the first AP spikes after photostimulation and after the application of CAP (*P<0.05, Mann–Whitney test). (**e**) A second photostimulation in the presence of AzCA4 by *λ*=365 nm light can be triggered 5 min after the first photostimulation; data are represented as the mean and s.d. of the number of spikes per 1 s bins of all activated C-MH.

We next evaluated the effects of AzCA4 on heat-sensitive C-fibre nociceptors (C-MH) in the *ex vivo* skin nerve preparation of the saphenous nerve. In wt mice, 19 out of 35 C-MH responded to AzCA4 photoswitching compared with none of the 11 C-MH examined in *Trpv1*^*−/−*^ mice (*P*<0.001, *χ*^2^-test; Fig. 5a). We compared the activation of AzCA4 with that of CAP on C-MH in terms of the latency time to the first AP spike, as well as the AP discharge rates. Peak discharge rates during photostimulation with *λ*=365 nm surged rapidly to 9.6 spikes per second and lasted on average 11.0±6.9 s (Fig. 5b). The average peak discharge rates of C-MH due to AzCA4 photoswitching were not significantly different from CAP stimulation (Fig. 5c). However, the mean latency time to the first AP spike on irradiation with *λ*=365 nm in the presence of AzCA4 was significantly shorter (1.9±0.5 s; mean±s.e.m.) when compared with the latencies to the first spike after CAP application (9.5±3.5 s) (Fig. 5d, Mann–Whitney test, *P*<0.05). The discharges of C-MH in response to AzCA4 were usually transient and adapted during the 20 s photostimulation period. These responses were completely reproducible after a 5 min recovery interval, during which AzCA4 was kept on the receptive field with no signs of desensitization (Fig. 5e). Therefore, the photoswitching of AzCA4 could selectively activate TRPV1 receptors on the peripheral terminals of cutaneous C-MH to a similar magnitude as CAP. Advantageously, AzCA4 had the ability to trigger a more rapid neuronal response and did not require washout of the drug.

### Serotonin and BK sensitized DRG neurons to AzCA4

TRPV1-mediated hyperalgesia is a complex process, which underlies inflammatory pain^19^,^33^. At sites of tissue injury, a number of chemical agents are released, which cause an inflammatory response resulting in an increased thermal pain sensation in response to normally non-painful stimuli. Several G protein-coupled receptors (GPCRs) mediate TRPV1 sensitization^33^,^34^. BK- and 5-HT-triggered GPCR cascades are able to decrease the threshold for TRPV1 activation and increase the number of receptors at the cell surface^5^,^33^,^35^,^36^.

**Figure 6:**
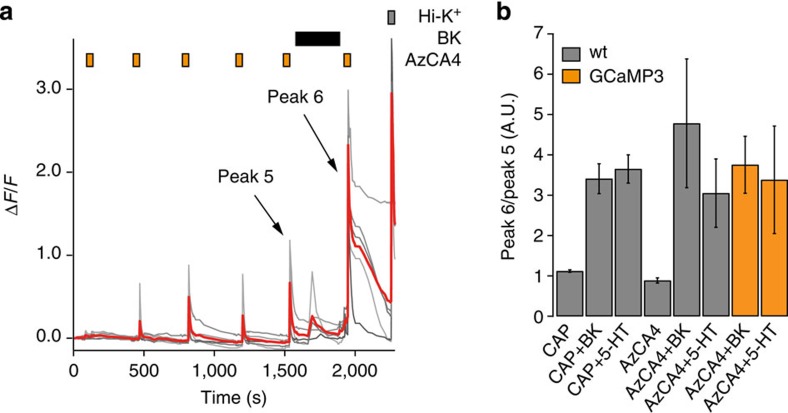
Serotonin and BK sensitized TRPV1 to AzCA4. Intracellular Ca^2+^ imaging showed that both BK and 5-HT sensitized TRPV1 to CAP (100 nM) and AzCA4 (200 nM), with ultraviolet irradiation (*λ*=365 nm, 5 s) in cultured wt DRG neurons. (**a**) After the application of BK (200 nM), an increased intensity and duration of Ca^2+^ influx was observed on application of AzCA-4 with ultraviolet irradiation when compared with the previous pulse. The neurons responded to a Hi-K^+^ solution (100 mM). Shown here are five representative traces (grey) and average Δ*F*/*F* value (red). (**b**) TRPV1 sensitization experiments in wt mouse DRG neurons (grey) and the *Trpv1*^*Cre/GCaMP3*^ mouse line (orange). The results were plotted as the ratio of peak heights for Peak 6/Peak 5 for the wt mouse as follows: CAP with no sensitization agent (*n*=520); CAP with BK (*n*=210); CAP with 5-HT (*n*=175); AzCA4 with no sensitizing agent (*n*=36); AzCA4 with BK (*n*=15); and AzCA4 with 5-HT (*n*=12). For the *Trpv1*^*Cre/GCaMP3*^ mouse line, the results are plotted for the following: AzCA4 with BK (*n*=28) and AzCA4 with 5-HT (*n*=10). Error bars were calculated as s.e.m.

We used intracellular Ca^2+^ imaging in cultured wt mouse DRG neurons to determine whether these endogenous inflammatory agents still sensitized TRPV1 towards AzCA4. Our experiments showed that TRPV1 became sensitized to AzCA4 (200 nM) after the application of both BK (200 nM) and 5-HT (100 μM) (Fig. 6a,b). After 5 pulses of AzCA4 and ultraviolet irradiation, a steady state of TRPV1 desensitization was reached and the cells were washed with the sensitizing agent for 5 min (Fig. 6a). In both cases, a final pulse of AzCA4 and ultraviolet irradiation resulted in increased levels of intracellular Ca^2+^ when compared with the previous pulse. Experiments with both inflammatory agents produced similar results to those achieved when using CAP as the channel agonist (Fig. 6b and Supplementary Fig. 11). These results suggest that AzCA4 could be used as a tool to study the involvement of TRPV1 in inflammatory pain.

### AzCA4 is compatible with genetic tools

Genetically encoded Ca^2+^ indicators, such as GCaMP3 (ref. 37), have proven very useful for the study of neuronal activity *in vitro* and *in vivo*. We therefore tested the ability of AzCA4 to work in combination with this genetic tool. We crossed a mouse line that expresses the Cre recombinase under the promoter of TRPV1 (ref. 38), with a reporter mouse line that expresses GCaMP3 in a Cre-dependant manner (*Trpv1*^*Cre/GCaMP3*^). As the majority of DRG neurons express TRPV1 at embryonic stage E14.5 (ref. 31), we observed that 90% of the DRG neurons in culture showed a faint basal fluorescence, confirming that recombination had occurred in most neurons at an earlier developmental stage.

However, on application of CAP or AzCA4 alongside ultraviolet irradiation, only 30% of the neurons showed a Ca^2+^-dependent increase in fluorescence. This confirmed not only the correct function and localization of GCaMP3 in TRPV1-positive DRG neurons, but also that this small molecular photoswitch, AzCA4, can be used in combination with a genetic tool.

To further confirm the applicability of AzCA4 and the use of genetically encoded Ca^2+^ indicators to address physiologically relevant issues, we repeated the sensitization experiments on the *Trpv1*^*Cre/GCaMP3*^ mouse line (Fig. 6b, orange). These results again confirmed that TRPV1 could be sensitized to AzCA4 by BK or 5-HT, even in the *Trpv1*^*Cre/GCaMP3*^ mouse line.

### QX-314 can be selectively transported into DRG neurons

**Figure 7:**
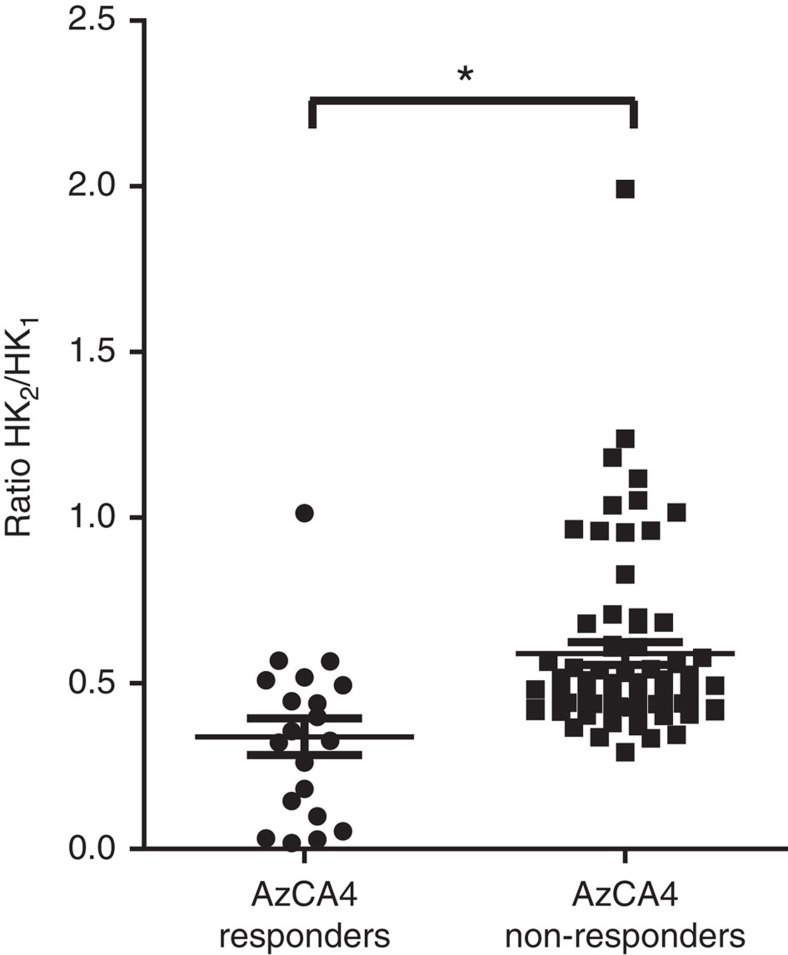
QX-314 could be shuttled into TRPV1-positive DRG neurons using AzCA4. After the co-application of AzCA4 (200 nM) and QX-314 (5 mM), the peak heights of the Ca^2+^ signals of the Hi-K^+^ (40 mM) pulses before (HK_1_) and after (HK_2_) drug application were compared. The results were plotted as the peak height ratio of two pulses, HK_2_/HK_1_, calculated for both AzCA4 responding and AzCA4 non-responding neurons. The error bars were calculated as s.e.m.

QX-314 is a permanently charged lidocaine derivative that blocks Na^+^ channels from the intracellular side but is unable to penetrate the plasma membrane due to its charged nature^39^. It has been shown that QX-314, and its photoswitchable derivative QAQ^40^, could be shuttled into cells via TRPV1 when co-applied with CAP, to open the channel^41^. We hypothesized that intracellular Ca^2+^ build-up is not only caused by Ca^2+^ influx through TRPV1 but is a consequence of AP firing leading to Ca^2+^ release from intracellular stores. We showed AzCA4 (200 nM) in combination with *λ*=365 nm irradiation, opened TRPV1 and allowed QX-314 to enter TRPV1-positive neurons in the DRG. The cells were first washed with a Hi-K^+^ solution (40 mM) causing massive electrical activity. After the cells recovered, four pulses of AzCA4 (200 nM, 5 s at *λ*=365 nm) reached a steady state of TRPV1 desensitization. Next, AzCA4 was co-applied with QX-314 (5 mM), followed by another pulse of AzCA4 and a final pulse of the Hi-K^+^ solution. By comparing the peak heights of the first and final Hi-K^+^ pulses (HK_1_ and HK_2_, respectively), we observed that the cells that were responsive to AzCA4 showed a lower HK_2_/HK_1_ ratio when compared with cells that did not respond to AzCA4 (Fig. 7). These results indicate that in combination with AzCA4, TRPV1 could be used as an import channel to localize a charged anaesthetic in TRPV1-positive cells, only.

## Discussion

In this study, we present eight photoswitchable FAs, the FAAzos. We expect that these lipophilic modules will allow us to place a variety of lipid-modulated biological targets under photopharmacological control. The FAAzos are FAs mimetics, resembling in particular highly unsaturated FAs such as arachidonic acid. These compounds could be used as molecular building blocks for the construction of more complicated photolipids, which could facilitate the optical control of a wide range of ion channels, GPCRs and enzymes associated with FA signalling.

We hypothesized that an azobenzene photoswitch would be best suited towards integration into the FA backbone, as its hydrophobic nature may cause only a minimal disruption to the properties of a natural aliphatic chain. The FAAzos allow the position of the switch to be fine-tuned to complement structure–activity relationships between the ligand and its target. As a first installment illustrating this concept, we showed that the FAAzos could be incorporated into other photolipids through a simple amide coupling reaction. In HEK293T cells, three AzCAs stood out as the most promising candidates to enable the optical control of TRPV1 and showed significant, light-dependant efficacy at concentrations as low as 100 nM. All three compounds were more potent in their *cis*-configuration. Owing to the structural similarities between the AzCAs and other vanilloid TRPV1 agonists, we presume that the AzCAs act on the same vanilloid binding site as CAP, olvanil and arvanil. This is further supported by the requirement of the vanilloid headgroup, as was shown by the inactivity of FAAzo2–4. Channel activation by AzCA4 can also be completely blocked by the application of capsazepine, a known competitive antagonist for CAP, suggesting a common binding site and mode of activation.

We then demonstrated that AzCA4 was a powerful modulator of DRG neurons in wt mice and the *Trpv1*^*Cre/GCaMP3*^ mouse line. Control experiments in untransfected HEK293T cells and *Trpv1*^*−/−*^ mice showed no response to AzCA4, even on light stimulation. These results rule out the possibility of action through off-target mechanisms. This characteristic is essential for the study of signal transduction in the nociceptive neurons involved in inflammatory pain. To this end, we envision that AzCA4 could be used to study TRPV1-mediated hyperalgesia and the physiological processes involved with the inflammatory state that occurs at sites of tissue injury. We showed that application of components of the so-called ‘inflammatory soup', such as BK or 5-HT, could sensitize TRPV1 to AzCA4 in DRG neurons. Furthermore, we proved that AzCA4 could be used in conjunction with genetic tools such as the GCaMP3 Ca^2+^ indicator selectively expressed in TRPV1-positive mouse neurons.

Importantly, AzCA4 allowed for greater temporal control over TRPV1 than that which can be achieved by other small-molecule agonists such as CAP, olvanil and arvanil. By working at an optimized concentration, AzCA4 showed no activity towards TRPV1 in its *trans*-configuration, but rapid TRPV1 activation was observed when it was isomerized to its *cis*-configuration. This ON and OFF effect became even more pronounced in neuronal systems when the nonlinear, ‘all or nothing,' nature of the AP took effect. Previous studies have suggested that the rate of TRPV1 activation determines the balance between agonist potency and pungency^42^,^43^,^44^,^45^. Molecular weight and lipophilicity normally define the pharmacodynamics of TRPV1 agonists^42^,^43^. In the case of AzCAs, light provides another level of control. We showed that the magnitude and rate of cellular activation could be precisely tuned by adjusting the ON wavelength between *λ*=350 and 390 nm. Therefore, AzCAs could provide a platform for the further understanding of hyperalgesia and could lead to the development of new anaesthetics.

When using CAP to stimulate C-fibres in the saphenous nerve, a relatively slow increase in AP firing was observed (Fig. 5b,c). This effect was probably caused by the time required for CAP to diffuse through the skin and plasma membrane to reach the vanilloid binding site^46^,^47^,^48^. We showed that AzCA4 could be applied to neurons as its dark-adapted, relatively inactive configuration. On isomerization, TRPV1 was activated and AP firing was immediately observed. It is this characteristic that makes AzCA4 a useful tool for the study of nociception, a process that relies on the rapid transmission of noxious stimuli from the periphery towards the coordinating centres of the central nervous system. AzCA4 also possesses significant advantages when compared with other small molecules that have been used to place TRPV1 under the control of light. Caged CAP is a useful tool that has increased the level of control with which we are able to activate TRPV1 (ref. 49). However, compound uncaging and TRPV1 activation is a non-reversible, one-shot process. Repeated activation by uncaging relies on fast-acting transporters or deactivating enzymes to clear the synapse of the free ligand^50^. The fact that CAP and its analogues exhibit long-lasting effects suggests that they do not undergo transporter-mediated reuptake or significant enzymatic hydrolysis. These aspects are circumvented by the use of AzCA4, which allows for successive rounds of activation/inactivation without the requirement for washout of the drug. Previously, we showed that photoswitchable TRPV1 antagonists can optically control the activity of a constitutively active agonist on photoswitching^25^. In comparison, AzCA4 greatly simplifies this system and is capable of activating TRPV1 directly. This permits the optical control of neuronal excitability without the use of a second factor. Advantageously, AzCA4 is relatively inactive in the dark; therefore, a more rapid and reproducible initiation of activity can be achieved after it has distributed itself uniformly within more complex tissues.

In conclusion, this study provides the first application of photopharmacology to lipid signalling. Given the ubiquitous distribution of FA derivatives at all levels of nature, we envision that the FAAzos and their conjugates will emerge as broadly applicable tools for the optical control of biological functions which rely on protein–lipid interactions.

## Methods

### Whole-cell electrophysiology in HEK293T cells

HEK293T cells (obtained from the Leibniz-Institute DSMZ: 305) were incubated at 37 °C (10% CO_2_) in DMEM medium+10% fetal bovine serum and were split at 80%–90% confluency. For cell detachment, the medium was removed and the cells were washed with calcium-free PBS buffer and treated with trypsin for 2 min at 37 °C. The detached cells were diluted in growth medium and plated on acid-etched coverslips coated with poly-L-lysine in a 24-well plate. Cells (50,000) were added to each well in 500 μl standard growth medium along with the DNA (per coverslip: 500 ng TRPV1–YFP^28^) and JetPRIME transfection reagents, according to the manufacturer's instructions (per coverslip: 50 μl JetPRIME buffer, 0.5 μl JetPRIME transfection reagent). The transfection medium was exchanged for normal growth media 4 h after transfection and electrophysiological experiments were carried out 20–40 h later. Whole-cell patch clamp experiments were performed using a standard electrophysiology setup equipped with a HEKA Patch Clamp EPC10 USB amplifier and PatchMaster software (HEKA Electronik). Micropipettes were generated from ‘Science Products GB200-F-8P with filament' pipettes using a Narishige PC-10 vertical puller. The patch pipette resistance varied between 5 and 9 MΩ. The bath solution contained the following: Solution A (in mM: 150 NaCl, 6.0 CsCl, 1.0 MgCl_2_, 1.5 CaCl_2_, 10 HEPES and 10 glucose (adjusted to pH 7.4 with 3 M NaOH))^51^ or Solution B (in mM: 140 NaCl, 5 KCl, 5 HEPES, 1 MgCl and 5 glucose (adjusted to pH 7.4 with 3 M NaOH)). The pipette solution contained th following: Solution A' (in mM: 150 NaCl, 3 MgCl_2_, 10 HEPES and 5 EGTA (adjusted to pH 7.2 with 3 M NaOH)) or solution B' (in mM: 100 K-gluconate, 40 KCl, 5 HEPES, 5 MgATP and 1 MgCl (adjusted to pH 7.2 with 1 M KOH)). The cells were first visualized to contain TRPV1–YFP by irradiation at *λ*=480 nm using a Polychrome V (Till Photonics) monochromator. All cells had a leak current between −15 to −300 pA on breakin at −60 mV. All voltage clamp measurements were carried out at a holding potential of −60 mV. The cells were held at 0 pA for current clamp measurements. The compounds were applied by puff pipette using a ‘Toohey Spritzer pressure system IIe' at 25 psi. The puff pipette resistance varied between 3 and 5 MΩ. All experiments were performed at room temperature.

### Determination of AzCA photoswitching properties

The photoswitching properties of the prepared compounds were assessed using whole-cell voltage clamp electrophysiology in HEK293T cells transiently expressing TRPV1–YFP^28^. The compounds were dissolved as stock solutions in dimethylsulfoxide (DMSO; 2–6 mM) and then diluted into warmed extracellular solution at a concentration of 1 μM. The cell was held at −60 mV and voltage ramps (−100 to +100 mV over 5 s) were applied under both *λ*=450 nm and *λ*=350 nm irradiation. The AzCA derivative (1 μM) was then constantly applied (puff pipette application) while switching between the two wavelengths until a steady state of activity/desensitization was observed on photoswitching (Supplementary Fig. 4a). The voltage ramps were applied again under each wavelength and the current change (Δ*I*) between the baseline (−60 mV) and the ramp maximum (+100 mV) was recorded (Supplementary Fig. 4b). Δ*I*(350 nm) was normalized to Δ*I*(450 nm) and this potentiation factor was averaged over multiple cells and plotted in Fig. 2b. TRPV1 activation and inactivation kinetics were determined by exponential curve fitting in Igor Pro.

### Dorsal root ganglion neuronal culture

DRG were quickly dissected, collected in ice-cold DRG medium and digested in 1 mg ml^−1^ Collagenase IV (Gibco) at 37 °C for 50 min, to dissociate the tissue, followed by incubation in 0.05% trypsin (Gibco) in PBS at 37 °C for 15 min. The trypsin was removed and cells were re-suspended in 1 ml of DRG medium. After gentle trituration, the DRGs were loaded onto a 2-ml BSA pillow and centrifuged at 250*g* for 10 min, to separate the myelin and debris. The resulting cell pellet was suspended in 50 μl fresh DRG medium and plated onto poly-D-lysine (100 μg ml^−1^) and laminin (10 μg ml^−1^)-coated coverslips. The cells were flooded with medium 30 min after plating.

### Whole-cell electrophysiology in cultured DRG neurons

Electrophysiological recordings of DRG neurons from 14- to 21-day-old mice were performed using a HEKA 10 amplifier (HEKA Instrument) and an ITC Analog Digital Converter (HEKA) in whole-cell voltage clamp or current clamp configuration. The currents were filtered with a built-in 5 kHz 8-pole Bessel filter and digitized at 50 kHz. The currents were analysed using Clampfit 10.3 (Molecular Devices) and graphs were plotted in Prism 5 (Graphpad). Experiments were performed 1–3 days after plating. The Trpv1^Cre^ and the GCaMP3 reporter mice were acquired from Jackson Laboratory. DRG neurons were prepared for imaging from 25-week-old *Trpv1*^*Cre/GCaMP3*^ mice.

The extracellular solution contained in mM: 150 NaCl, 5 KCl, 10 HEPES, 10 glucose, 2 CaCl_2_, 1 MgCl_2_. The intracellular solution contained (values in mM): 130 KCl, 10 HEPES, 10 EGTA, 1 CaCl_2_, 1 MgCl_2_, 2 MgATP, 1 NaGTP, 4 NaCl and 4 PhosphoCreatine.

Thick-walled electrodes (Harvard Apparatus, 1.17 × 0.87 mm, external and internal diameter, respectively) were pulled with a Sutter P-197 puller to a final resistance of 3–5 MΩ. After the breakthrough, the intracellular solution was allowed to dialyse the intracellular medium for at least 1 min before the beginning of the recordings. Series resistance compensation reached values between 70 and 90%. Neurons were selected to have a leak current <−100 pA on breakin at −60 mV. All experimental procedures were carried out in accordance with the State of Berlin Animal Welfare requirements and were approved by this authority.

### Intracellular calcium imaging

DRG neurons plated on a 5-mm glass coverslip were placed in a recording chamber of 300 μl volume (Harvard Apparatus) and were continuously perfused with extracellular solution at a rate of 2 ml min^−1^. The wt neuron cells were loaded with Cal-520 (5 μM, AAT-Bioquest) calcium dye for 1 h at 37 °C in the presence of 0.02% pluronic acid dissolved in Ringer's solution (values in mM): 140 NaCl, 5 KCl, 2 CaCl_2_, 2 MgCl_2_, 10 HEPES, 10 glucose, adjusted to pH 7.4. CAP (100 nM, Tocris) was dissolved in extracellular solution from a stock concentration of 10 mM in ethanol. Fluorescent images were acquired with Metafluor Software (Molecular Devices) and analysed in Clampfit. All experiments were performed at room temperature. The dye excitation was performed at *λ*=480 nm. The results were plotted as the change in fluorescence over baseline fluorescence (Δ*F*/*F*) as a function of time (s).

### *Ex-vivo* skin nerve preparation

The skin-nerve preparation was used as previously described, to record from single primary afferents^52^. Mice were killed with CO_2_ inhalation for 2–4 min, followed by cervical dislocation. The saphenous nerve and the shaved skin of the hind limb were dissected free and placed in an organ bath at 32 °C. The chamber was perfused with a synthetic interstitial fluid (SIF buffer) composed of (in mM): 123 NaCl, 3.5 KCl, 0.7 MgSO_4_, 1.7 NaH_2_PO_4_, 2.0 CaCl_2_, 9.5 Na-gluconate, 5.5 glucose, 7.5 sucrose and 10 HEPES at a pH of 7.4. The skin was placed with the corium side up in the organ bath for pharmacological application to the receptive fields of single sensory units. The saphenous nerve was placed in an adjacent chamber on a mirror, and under microscopy fine filaments were teased from the nerve and placed on the recording electrode. Electrical isolation was achieved with mineral oil. Signals from the filaments were amplified (Neurolog System, Digitimer Ltd) and sampled using a data acquisition system (PowerLab 4.0, ADInstruments). The receptive fields of individual mechanically sensitive C-fibre units were identified by manually probing the skin with the blunted tip of a glass probe. Their conduction velocities (calculated by dividing conduction distance over electrical latency for the first spike) were determined using an electrical stimulator (1 MΩ). All C-fibres studied were identified by mechanical probing and were heat sensitive (C-MH). The conduction velocities were in the C-fibre range (<1 m s^−1^). The thermal sensitivity of identified C-fibres was tested with a computer-controlled peltier device (3 × 5 mm, Yale University Medical School, Medical Instruments, New Haven, USA). A heat ramp was delivered from 32 to 48 °C at a rate of 1 °C s^−1^ to the mechanically localized receptive fields of single C-fibres. The receptive fields of heat-sensitive C-fibres were isolated with a metal cylinder ring (5 mm inner diameter, 10 mm in height and 1.44 g weight) for the administration of the drugs (AzCA4 or CAP). The metal ring was tested for leakage before drug application. A stock solution of AzCA4 was prepared in DMSO and diluted in SIF buffer. One hundred microlitres of a 1-μM AzCA4 solution dissolved in SIF buffer was applied into the ring. The pharmacological testing protocol with AzCA4 had four distinct successive phases: (1) 60 s recording time before the application of AzCA4; (2) 60 s recording after application of AzCA4; (3) 20 s of recording during photostimulation of AzCA4 with light-emitting diode (LED) light, *λ*=365 nm (Mic-LED-365, Prizmatix); (4) 20 s of recording during photoinhibition of AzCA4 with LED light, *λ*=460 nm (UHP-LED-460, Prizmatix). AzCA4 was kept in the ring for 300 s and a second photostimulation with LED light 360 nm for 20 s was applied to test the reproducibility of the initial responses. One hundred microlitres of CAP (1 μM, Sigma) were administered onto the receptive field of another set of heat-sensitive C-fibres for 20 s before washout. Recordings were obtained for 1 min before and 2 min after CAP administration. Spikes were discriminated offline, with the spike histogram extension of the software. Data were obtained from six skin-nerve preparations (2 *Trpv1*^*−/−*^ and 4 wt C57Bl/6 N mice). All experimental procedures were carried out in accordance with the State of Berlin Animal Welfare requirements and were approved by this authority.

### Compound switching

Compound switching for electrophysiology in HEK293T cells was achieved using a Polychrome V (Till Photonics) monochromator (intensity versus wavelength screen Supplementary Fig. 12) and the light beam was guided via a fibre-optic cable through the microscope objective and operated by the amplifier and PatchMaster software (HEKA Electronik).

Compound switching for electrophysiology in DRG neurons was achieved using a Prizmatix Mic-LED-365 high-power ultraviolet LED light source for illumination at *λ*=365 nm and the Prizmatix UHP-Mic-LED-460 ultra-high-power LED light source for illumination at *λ*=460 nm. The light beam was guided by a fibre-optic cable and pointed directly towards the cells from above at an angle of about 45° from the side. The distance between the end of the cable and the cells was no greater than 2 cm. Ultraviolet illumination during intracellular calcium imaging was also performed using this light source.

### Compound synthesis and characterization

All reagents and solvents were purchased from commercial sources (Sigma-Aldrich, TCI Europe N.V., Strem Chemicals and so on) and were used without further purification unless otherwise noted. Tetrahydrofuran was distilled under a N_2_ atmosphere from Na/benzophenone before use. Triethylamine was distilled under a N_2_ atmosphere from CaH_2_ before use. Further dry solvents such as ethyl acetate, benzene, dichloromethane, toluene, ethanol and methanol were purchased from Acros Organics as ‘extra dry' reagents and used as received. Solvents were degassed by sparging the freshly distilled solvent with argon gas in a Schlenk flask under ultrasonication using a Bandelin Sonorex RK510H ultrasonic bath for 20 min before use. Reactions were monitored by thin-layer chromatography on pre-coated, Merck Silica gel 60 F_254_ glass-backed plates and the chromatograms were first visualized by ultraviolet irradiation at 254 nm, followed by staining with aqueous ninhydrin, anisaldehyde or ceric ammonium molybdate solution and finally gentle heating with a heat gun. Flash silica gel chromatography was performed using silica gel (SiO_2_, particle size 40-63 μm) purchased from Merck.

Ultraviolet visible spectra were recorded using a Varian Cary 50 Bio UV-Visible Spectrophotometer with Helma SUPRASIL precision cuvettes (10 mm light path). All compounds were dissolved at a concentration of 25 μM in DMSO. Switching was achieved using a Polychrome V (Till Photonics) monochromator (intensity versus wavelength screen Supplementary Fig. 12). The illumination was controlled using PolyCon3.1 software and the light was guided through a fibre optic cable with the tip pointed directly into the top of the sample cuvette.

All NMR spectra were measured on a BRUKER Avance III HD 400 (equipped with a CryoProbe). Multiplicities in the following experimental procedures are abbreviated as follows: s, singlet; d, doublet; t, triplet; q, quartet; quint, quintet; sext, sextet; hept, heptet; br, broad; m, multiplet. Proton chemical shifts are expressed in parts per million (p.p.m., *δ* scale) and are referenced to the residual protium in the NMR solvent (CDCl_3_: *δ*=7.26, D_6_-DMSO: *δ*_H_=2.50). Carbon chemical shifts are expressed in p.p.m. (*δ* scale) and are referenced to the carbon resonance of the NMR solvent (CDCl_3_: *δ*=77.16, D_6_-DMSO: *δ*=39.52). Note: owing to the *trans/cis* isomerization of some compounds containing an azobenzene functionality, more signals were observed in the ^1^H and ^13^C spectra than would be expected for the pure *trans*-isomer. Only signals for the major *trans*-isomer are reported; however, the identities of the remaining peaks were verified by two-dimensional correlation spectroscopy, heteronuclear single quantum coherence and heteronuclear multiple bond correlation experiments. The atom-numbering system is defined as depicted in Supplementary Fig. 13. All relevant synthetic details and NMR spectra can be found in Supplementary Figs 14–46 and the Supplementary Methods.

Infrared spectra were recorded as neat materials on a PERKIN ELMER Spectrum BX-59343 instrument. For detection, a SMITHS DETECTION DuraSam-plIR II Diamond ATR sensor was used. The measured wave numbers are reported in cm^−1^.

Low- and high-resolution electron ionization mass spectra were obtained on a MAT CH7A mass spectrometer. Low- and high-resolution electrospray ionization mass spectra were obtained on a Varian MAT 711 MS instrument operating in either positive or negative ionization modes.

## Additional information

**How to cite this article:** Frank, J. A. *et al*. Photoswitchable fatty acids enable optical control of TRPV1. *Nat. Commun.* 6:7118 doi: 10.1038/ncomms8118 (2015).

## Supplementary Material

Supplementary Information Supplementary Figures 1-46, Supplementary Methods and Supplementary References
