# Supplementary material for: Photoswitchable fatty acids enable optical control of TRPV1
Source: Nat Commun. 2015 May 22;6:7118. doi: 10.1038/ncomms8118 (PMC4455067; doi:10.1038/ncomms8118)
Supplement: Supplementary Information — Supplementary Figures 1-46, Supplementary Methods and Supplementary References [file ncomms8118-s1.pdf]

Supplementary Figures

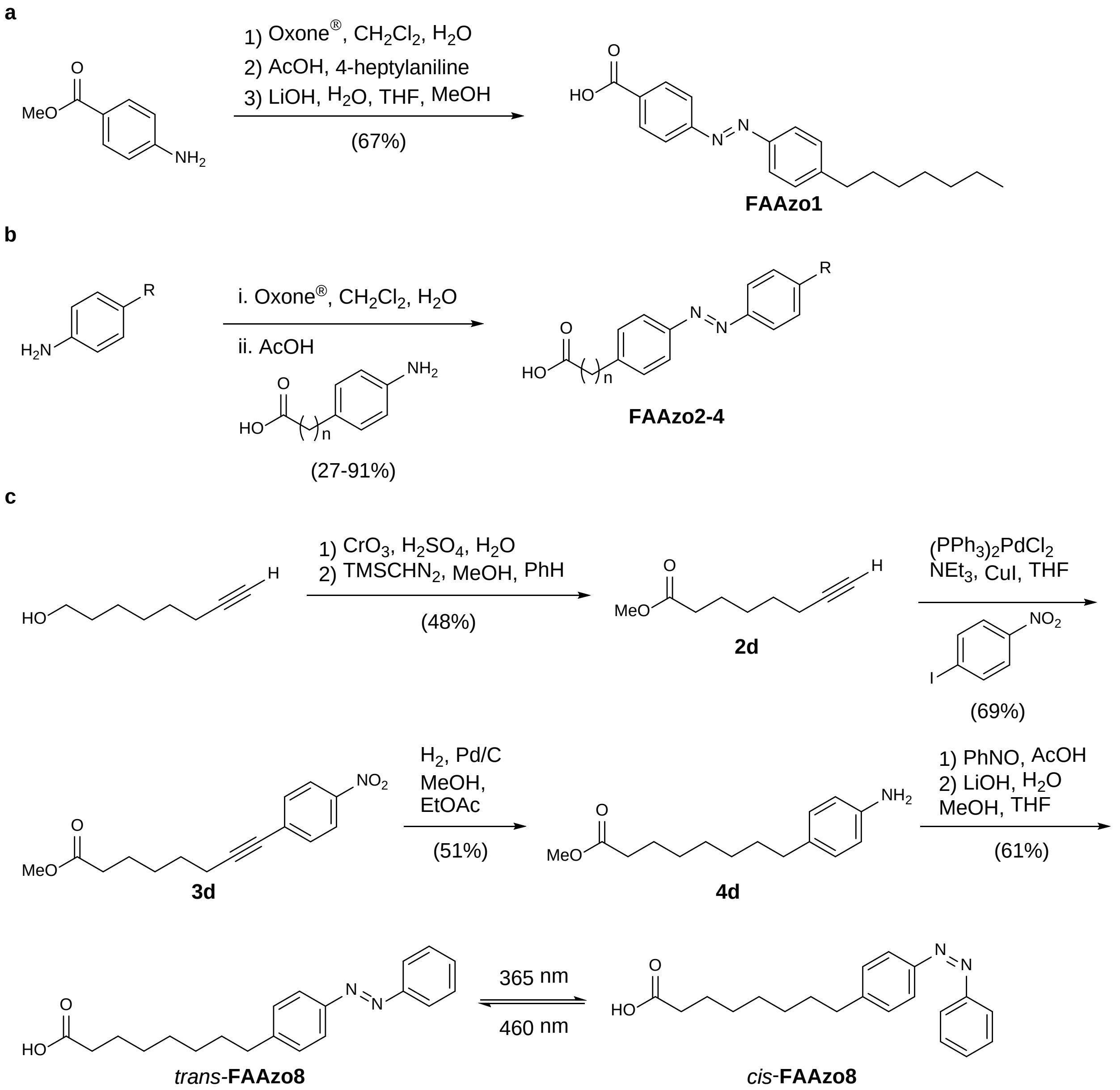

Supplementary Figure 1 | The syntheses of photoswitchable fatty acids, FAAzo1-8. (**a**) The synthesis of FAAzo1. (**b**) FAAzo2-4 were synthesized via the Mills reaction. FAAzo2: n = 1, R = hexyl (27%). FAAzo3: n = 2, R = pentyl (27%). FAAzo4: n = 3, R = butyl (91%). (**c**) FAAzo8 was synthesized in 6 steps from 7-octyn-1-ol in 10% overall yield. FAAzo5-7 were synthesized in an analogous fashion.

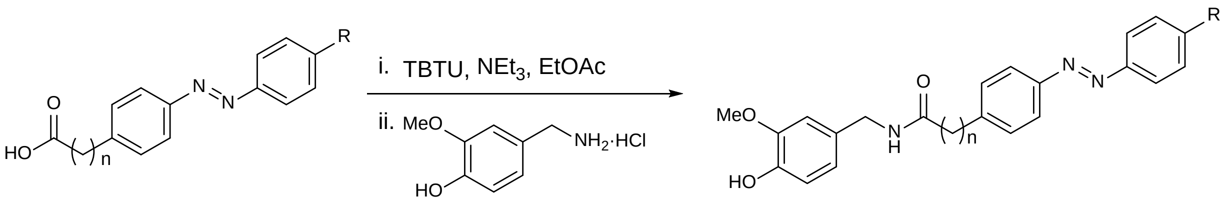

FAAzo1-8 (48-90%) **AzCA1-8**

Supplementary Figure 2 | The syntheses of photoswitchable vanilloids, AzCA1-8. Photoswitchable vanilloids, AzCA1-8, were prepared from the corresponding FAAzo via peptide coupling with vanillamine. AzCA1: n = 0, R = heptyl (89%). AzCA2: n = 1, R = hexyl (48%). AzCA3: n = 2, R = pentyl (89%). AzCA4: n = 3, R = butyl (87%). AzCA5: n = 4, R = propyl (90%). AzCA6: n = 5, R = ethyl (86%). AzCA7: n = 6, R = methyl (81%). AzCA8: n = 7, R = H (88%).

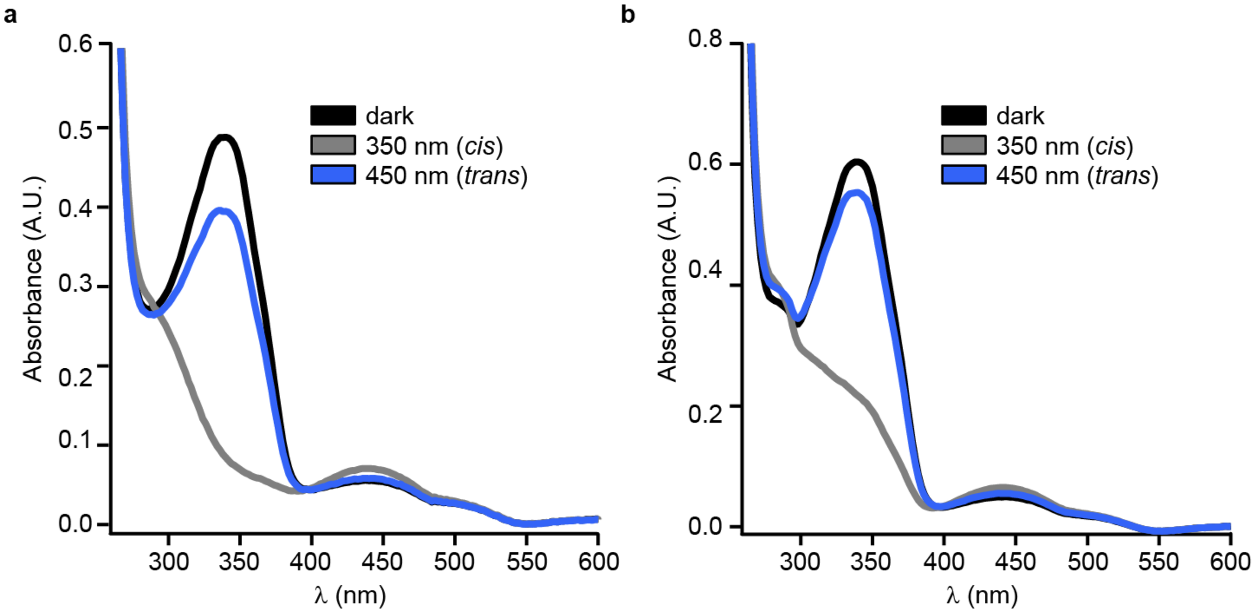

Supplementary Figure 3 | UV-Vis spectra of FAAzo- and AzCA-derivatives. Photoswitching of FAAzo4 (**a**) and AzCA4 (**b**) could be visualized with UV-Vis spectroscopy. The spectra for all FAAzo- and AzCA-derivatives showed nearly identical profiles among the group. An initial spectrum was recorded (*dark* adapted state, black) and then again following illumination at the λ_{max} for the π-π* transition (350 nm) for 3 min (*cis*-adapted state, grey). A third spectrum was recorded after irradiation at the λ_{max} for the n-π* transition (450 nm) for 3 min (*trans*-adapted state, blue).

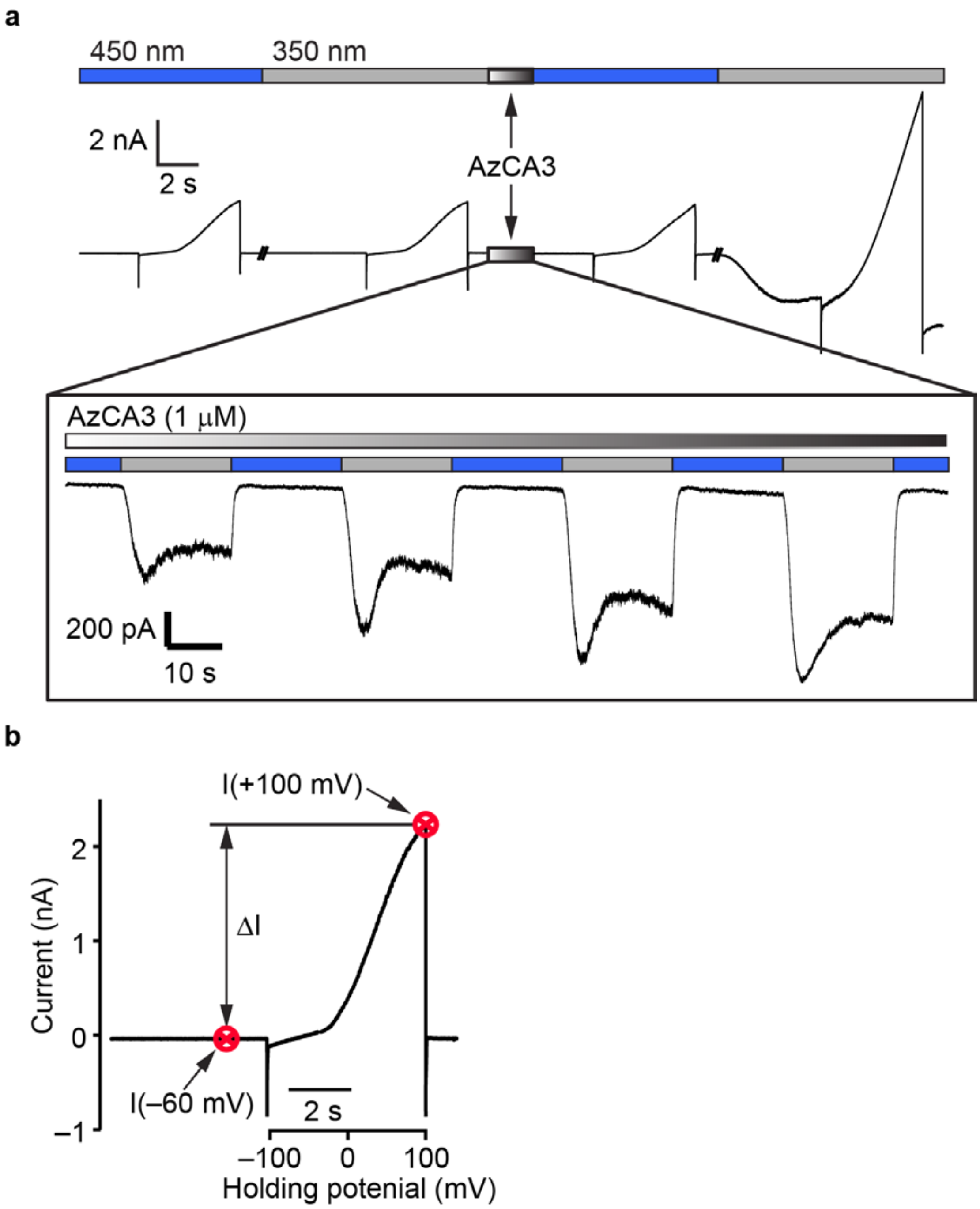

Supplementary Figure 4 | AzCA photoswitching characterization. A representative electrophysiological recording for AzCA3 (1 μM) is displayed here. (**a**) The typical procedure which was used to apply and assess each AzCA-derivative. (**b**) ΔI determination by voltage ramp analysis.

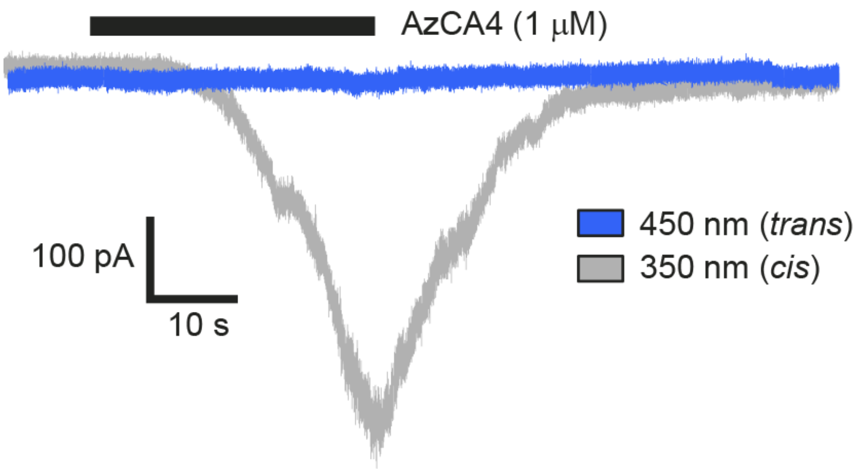

Supplementary Figure 5 | AzCA4 possesses a higher efficacy towards TRPV1 in its *cis*-configuration. AzCA4 was applied (1 μM) to HEK293T cells transiently expressing TRPV1-YFP under both λ = 350 nm (grey) and λ = 450 nm (blue) irradiation. When the membrane potential was clamped at –60 mV, an inward current was only observed upon irradiation with λ = 350 nm (*cis*), but not under λ = 450 nm (*trans*) upon application of AzCA4. This showed that AzCA4 was a more potent TRPV1 agonist in its *cis*-configuration. Both traces are sequential recordings from the same cell, and the first AzCA4 application was under λ = 350 nm irradiation.

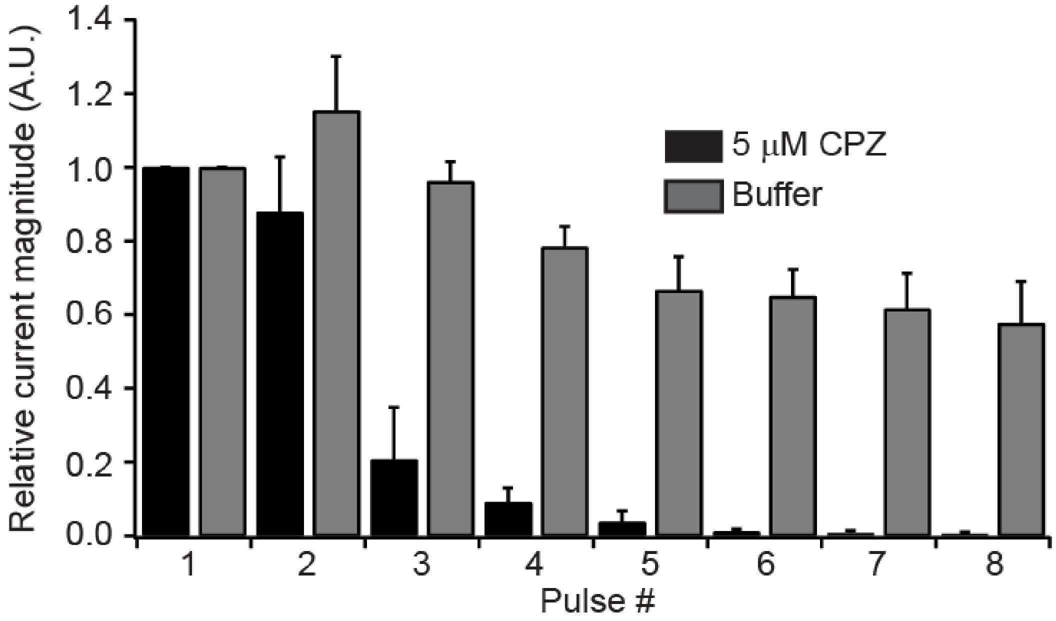

Supplementary Figure 6 | Capsazepine blocked AzCA4 photoswitching activity. AzCA4 (1 μM, puff application) was continuously applied upon photoswitching between λ = 350 nm and λ = 450 nm (10 s alternating pulses). After a steady state of activity/desensitization had been reached, the cells were washed with either capsazepine (CPZ, 5 μM in extracellular buffer, n = 4, black) or extracellular buffer (control, n = 4, grey). The maximum current amplitude for each UV pulse was calculated relative to the steady-state inward current and plotted as a function of pulse number after the washing was started. With buffer only, AzCA4 washout was slow and photoswitching persisted for >8 pulses. Addition of CPZ rapidly blocked TRPV1 currents upon photoswitching. Error bars were calculated as SEM.

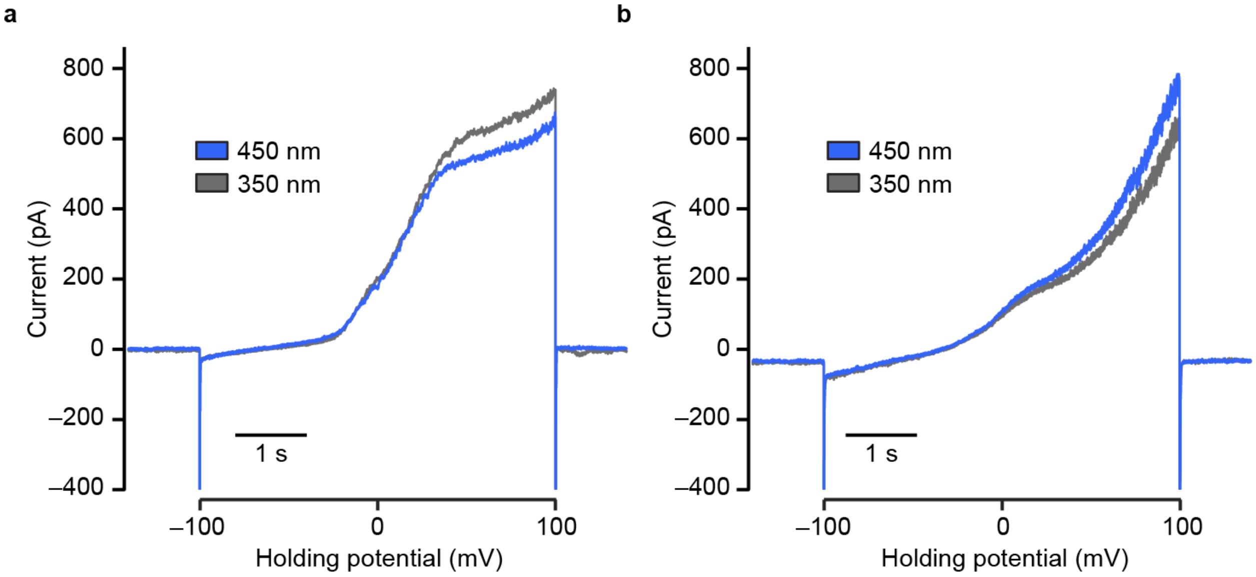

Supplementary Figure 7 | Capsaicin did not permit the optical control of TRPV1. Control experiments using whole-cell voltage clamp electrophysiology showed that capsaicin (CAP) did not endow optical control over TRPV1-YFP in HEK293T cells. Voltage ramps (from –100 mV to +100 mV over 5 s) were applied before (**a**) and after (**b**) the application of CAP (1 μM). These control experiments were performed using the same procedure detailed in **Supplementary Fig. 4**. The ramp profile changed between the two measurements due to residual TRPV1 activation, but no significant light-dependent currents were observed.

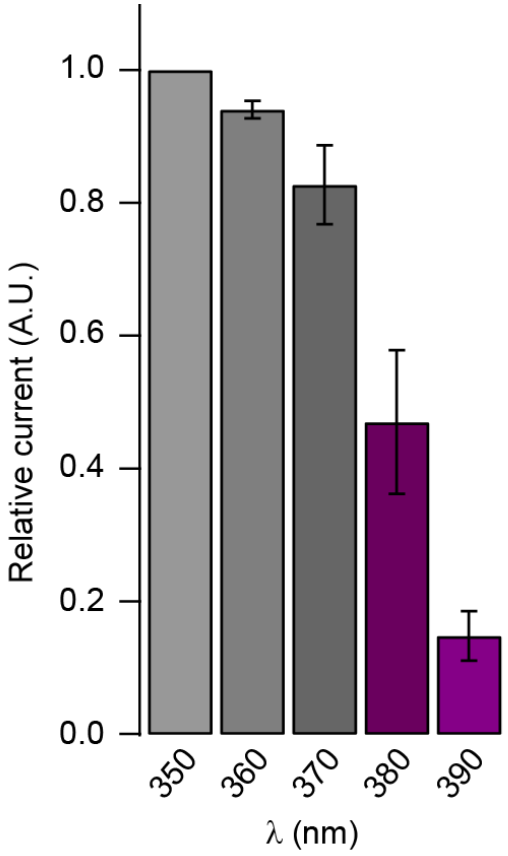

Supplementary Figure 8 | The magnitude of cellular currents was a function of the ON wavelength. When voltage clamped at –60 mV, the magnitude of the cellular current could be precisely controlled by adjusting the ON wavelength in HEK293T cells expressing TRPV1-YFP after the application of AzCA3 (1 μM). The values were calculated as the current change from the baseline while irradiating with λ = 450 nm, and the maximum inward current observed upon irradiation at the ON wavelength (λ = 350-390 nm) for 5 s. The resulting currents were normalized to the largest current value (with λ = 350 nm) and averaged over multiple cells (n = 3) to yield a relative current.

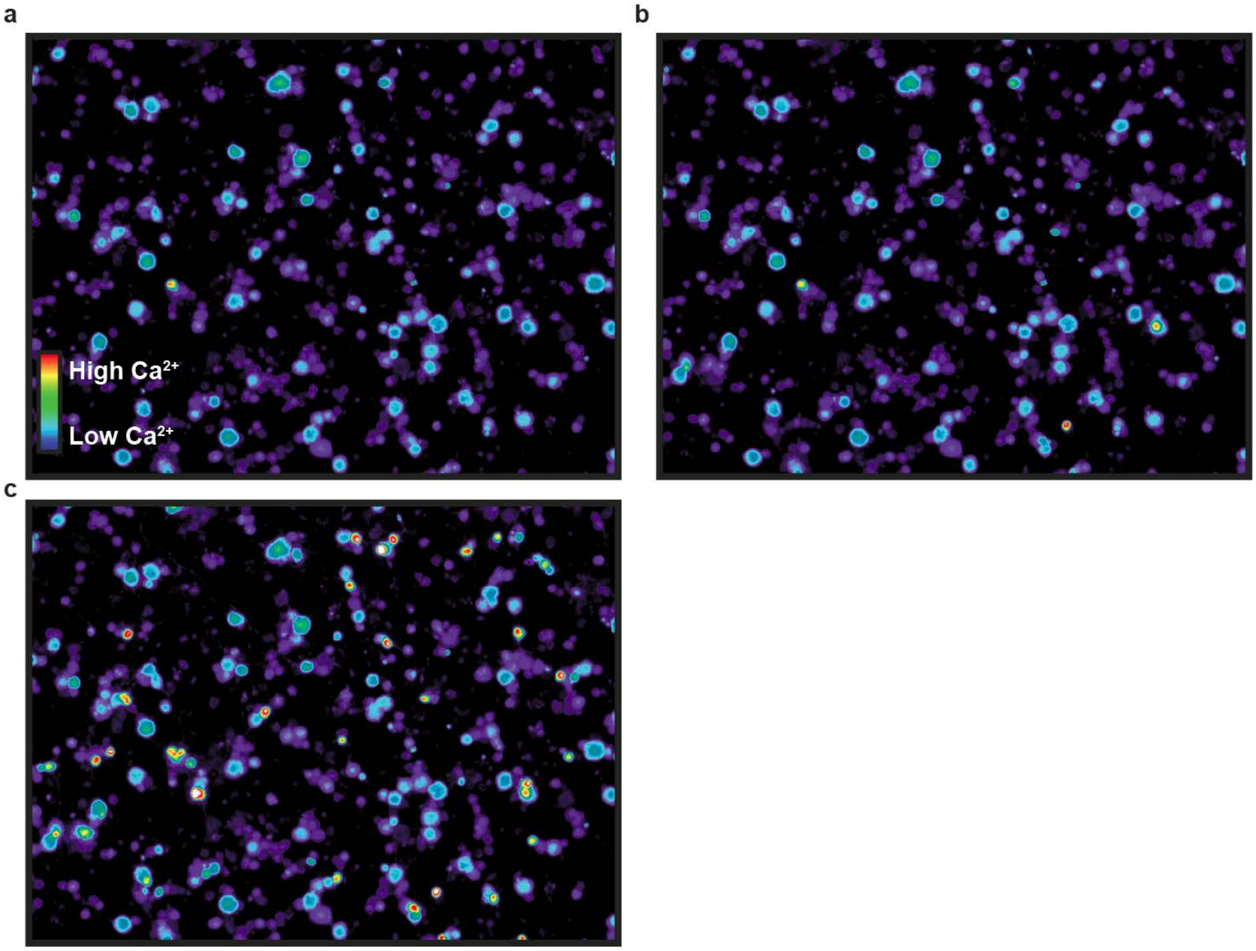

Supplementary Figure 9 | AzCA4 caused a significant increase in intracellular Ca^{2+} after a UV pulse in DRG neurons. (**a**) A baseline fluorescence image was recorded before the application of any compound. (**b**) A fluorescence image was then recorded 45 s after the application of AzCA4 (100 nM, bath application) and directly before UV irradiation. (**c**) A fluorescence image was then recorded immediately after a 5 s flash of λ = 365 nm irradiation. A marked increase in fluorescence was observed after UV irradiation in about 30% of the cultured neurons.

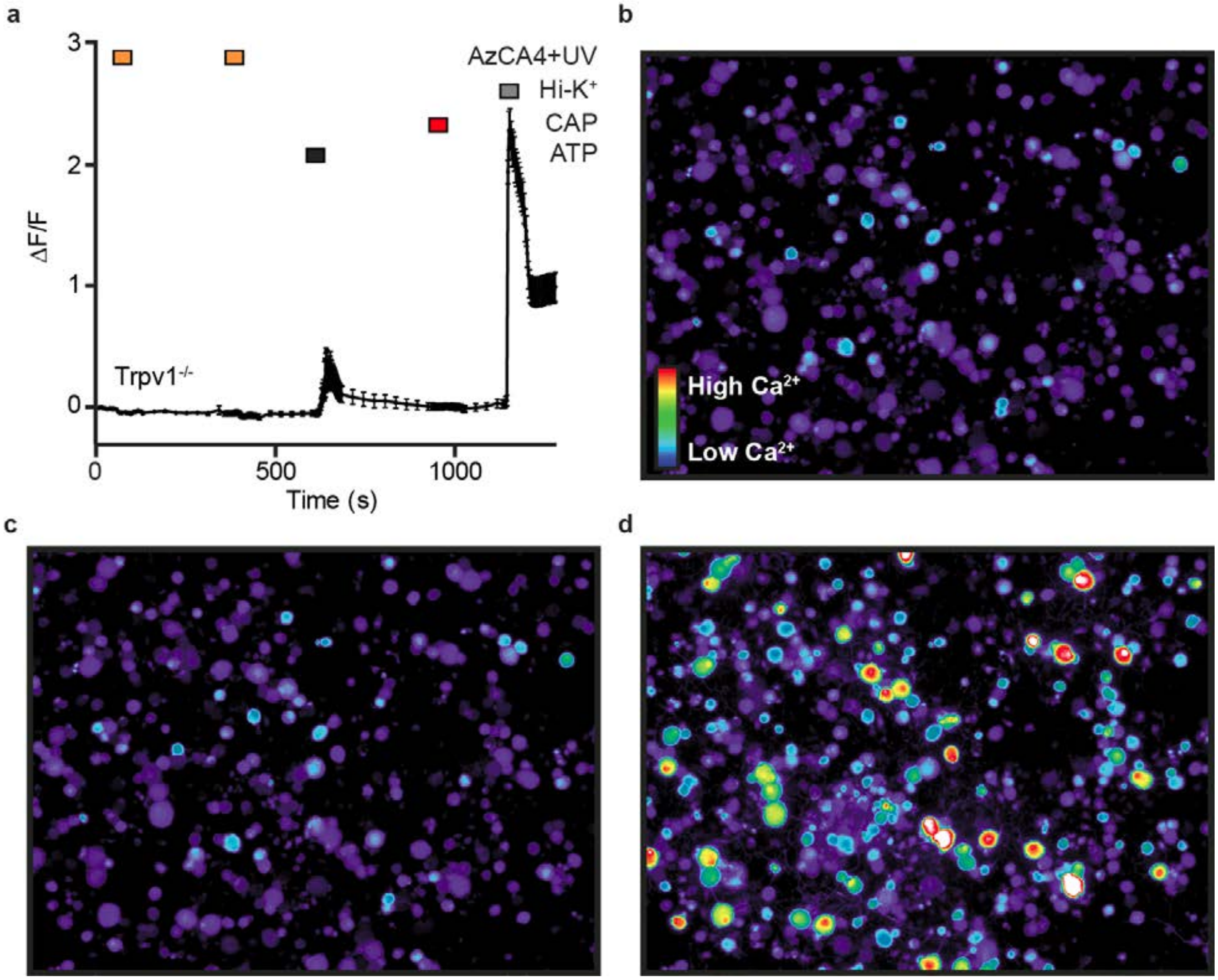

Supplementary Figure 10 | AzCA4 was selective for TRPV1-positive DRG neurons. Intracellular Ca^{2+} imaging revealed that AzCA4 had no action in cultured mouse Trpv1 knockout mutant (*Trpv1*$^{-/-}$) DRG neurons. (**a**) No fluorescence increase was observed upon application of AzCA4 (300 nM) and a UV pulse (λ = 365 nm, 5 s) or CAP (300 nM). The neurons were still responsive to ATP (20 μM) and a high potassium (Hi-K^+) (100 mM) solution (n = 11). (**b**) A baseline fluorescence image was recorded before the application of any compound. (**c**) Another image was then recorded after the application of AzCA4 (300 nM, bath application) and a UV pulse (λ = 365 nm, 5 s). No response was observed in any of the DRG neurons. (**d**) After application of a Hi-K^+ solution (100 mM), a large increase in intracellular Ca^{2+} was observed.

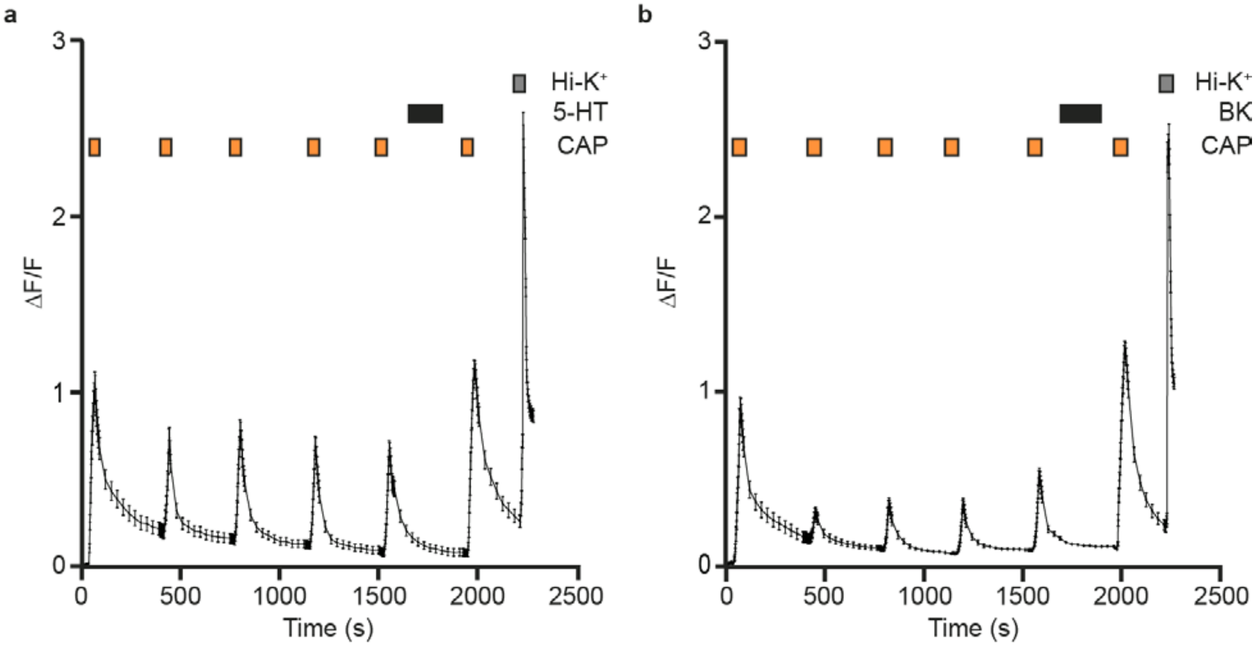

Supplementary Figure 11 | Bradykinin and serotonin sensitized TRPV1 to capsaicin. Intracellular Ca^{2+} imaging showed that cultured wild type mouse DRG neurons were sensitized towards CAP (100 nM) by both serotonin (5-HT, 100 μM, n = 175) (**a**) and bradykinin (BK, 200 nM, n = 210) (**b**). The cells were pulsed 5 times with CAP, followed by a 5 min wash with the sensitizing agent. A final CAP pulse showed a marked increase in both intensity and duration of the fluorescence increase in CAP-responding DRG neurons when compared with the previous pulse. The neurons still responded to a Hi-K^+ solution (100 mM).

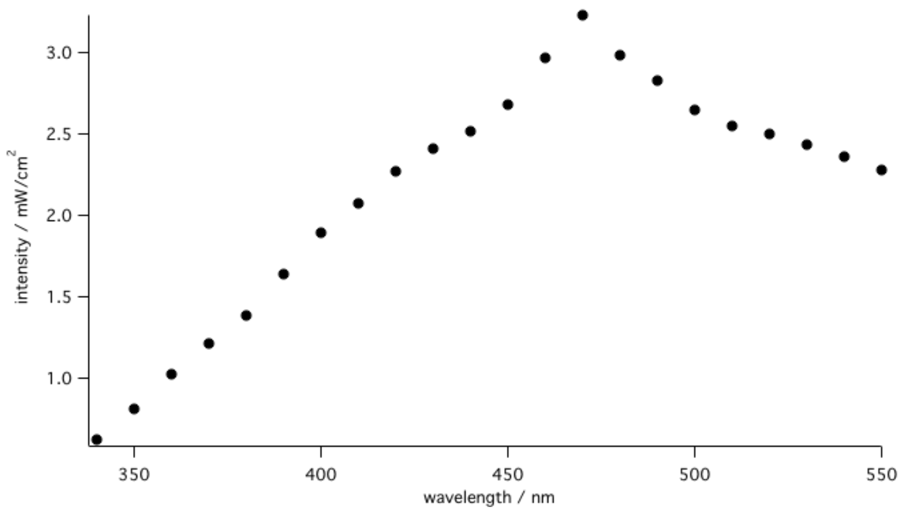

Supplementary Figure 12 | Wavelength vs. intensity screen of the Till Photonics Polychrome V.

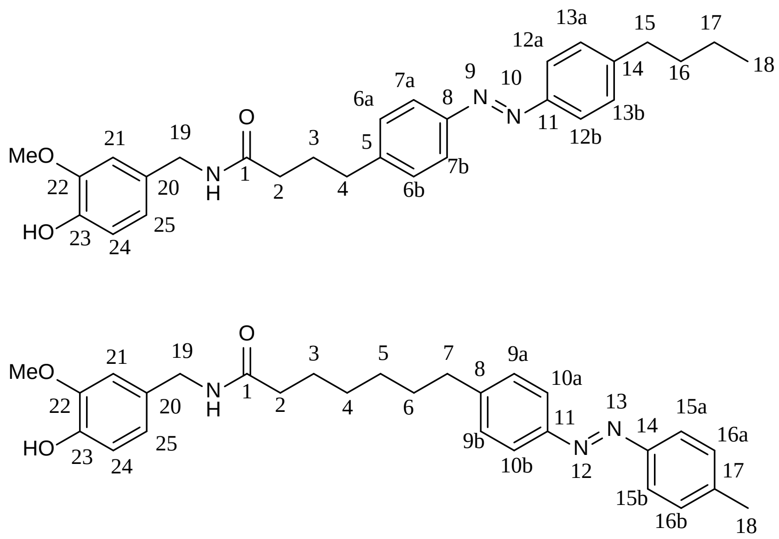

Supplementary Figure 13 | Photolipid numbering system. Numbering of the atoms in the lipid chains was set in relation to the carboxylic acid, which was labelled atom #1, and then increasing towards the distal end of the carbon chain.

NMR SPECTRA

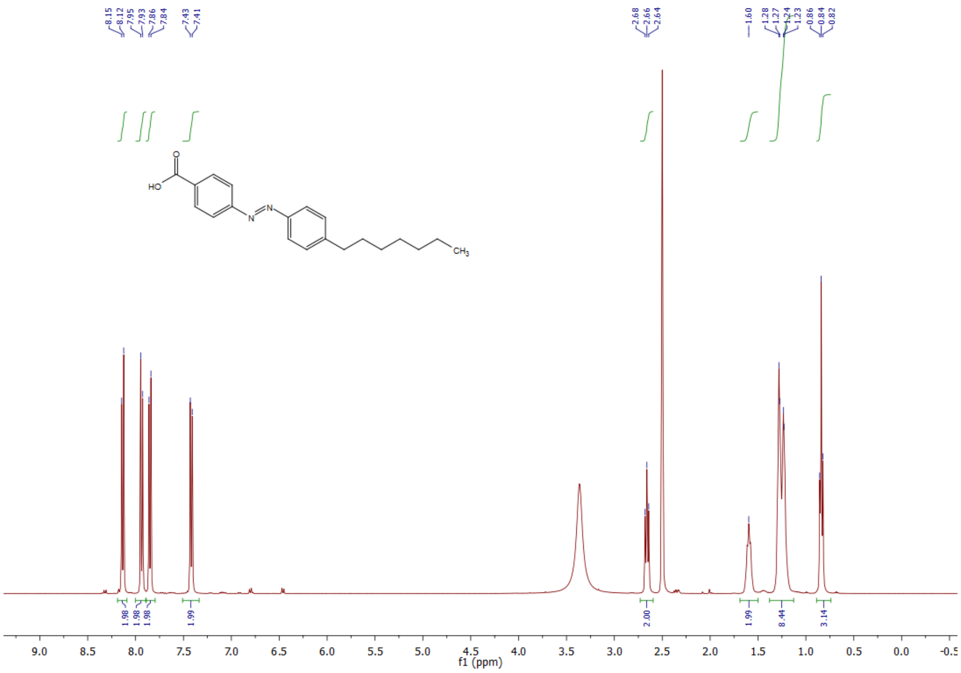

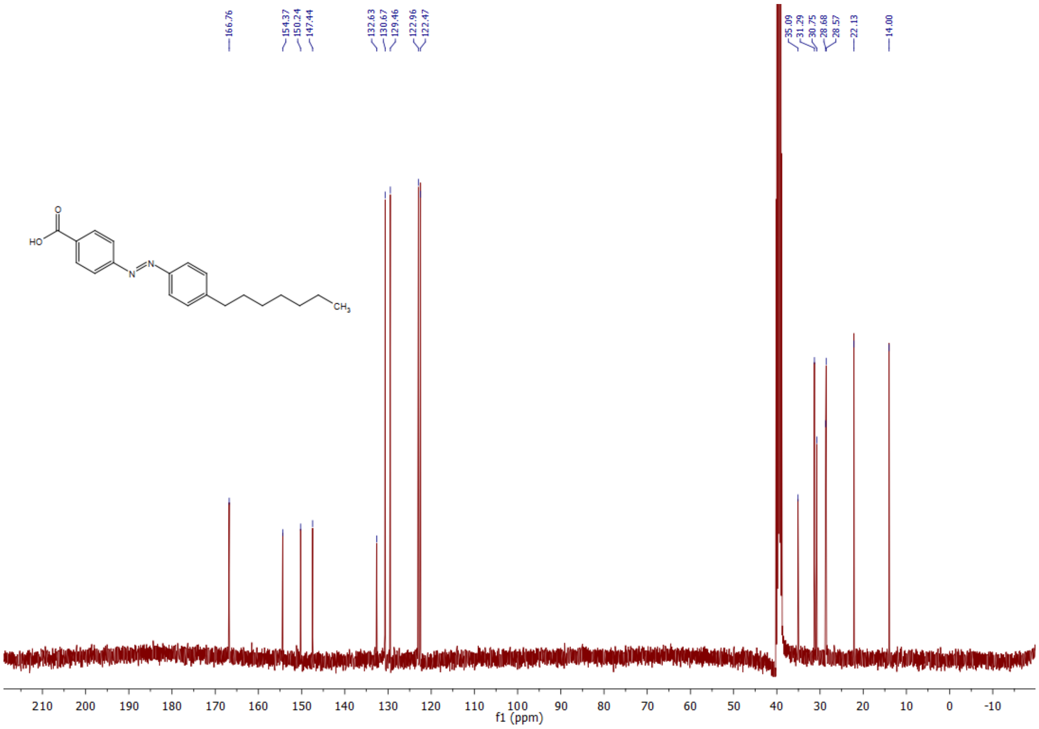

Supplementary Figure 14 | ^{1}H- and ^{13}C-NMR spectra of 4-((4-heptylphenyl)diazenyl)benzoic acid (**FAAzo1**).

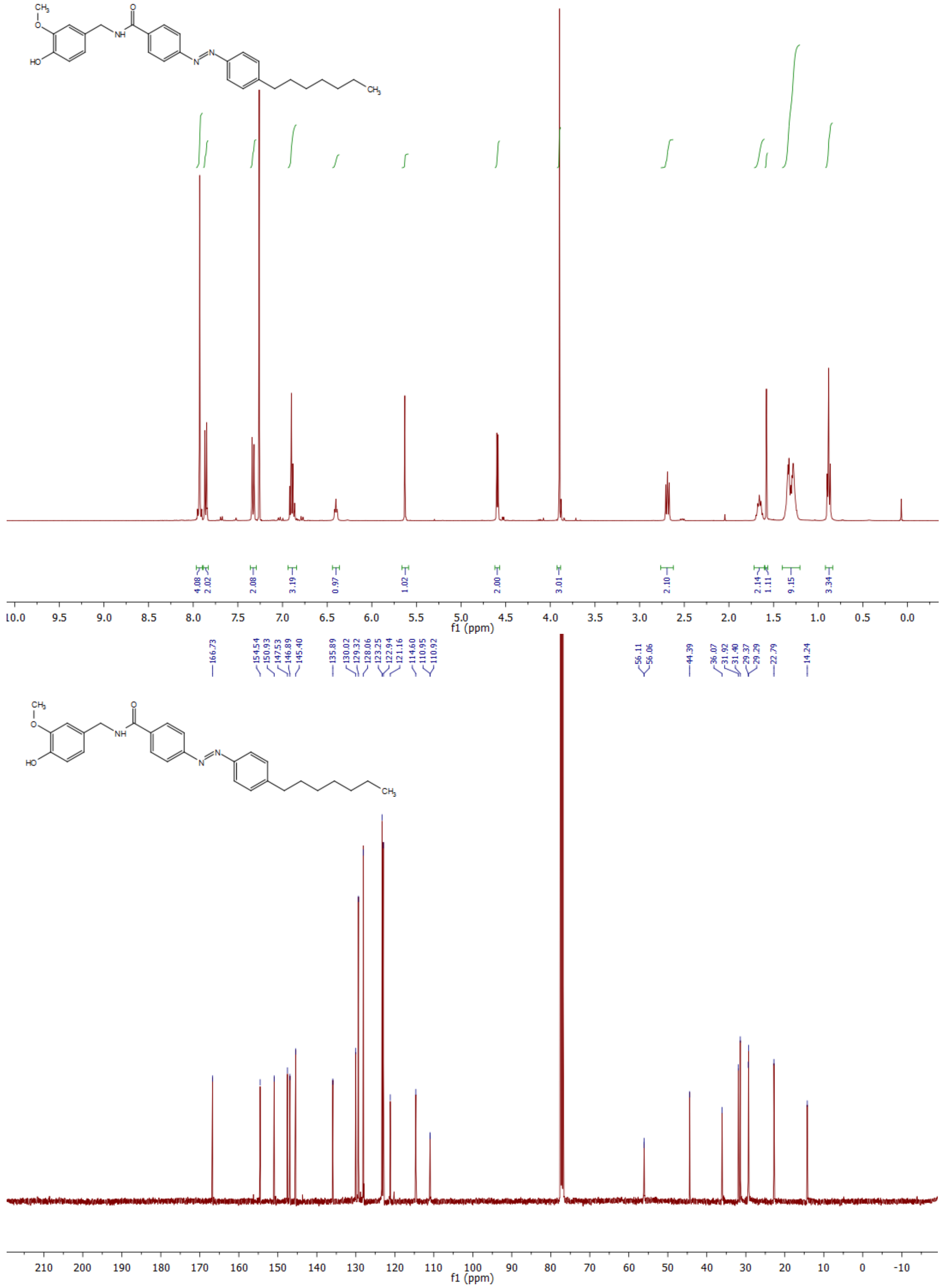

Supplementary Figure 15 | ^{1}H- and ^{13}C-NMR spectra of *N*-(4-hydroxy-3-methoxybenzyl)-4-((4-heptylphenyl)diazenyl)benzamide (**AzCA1**).

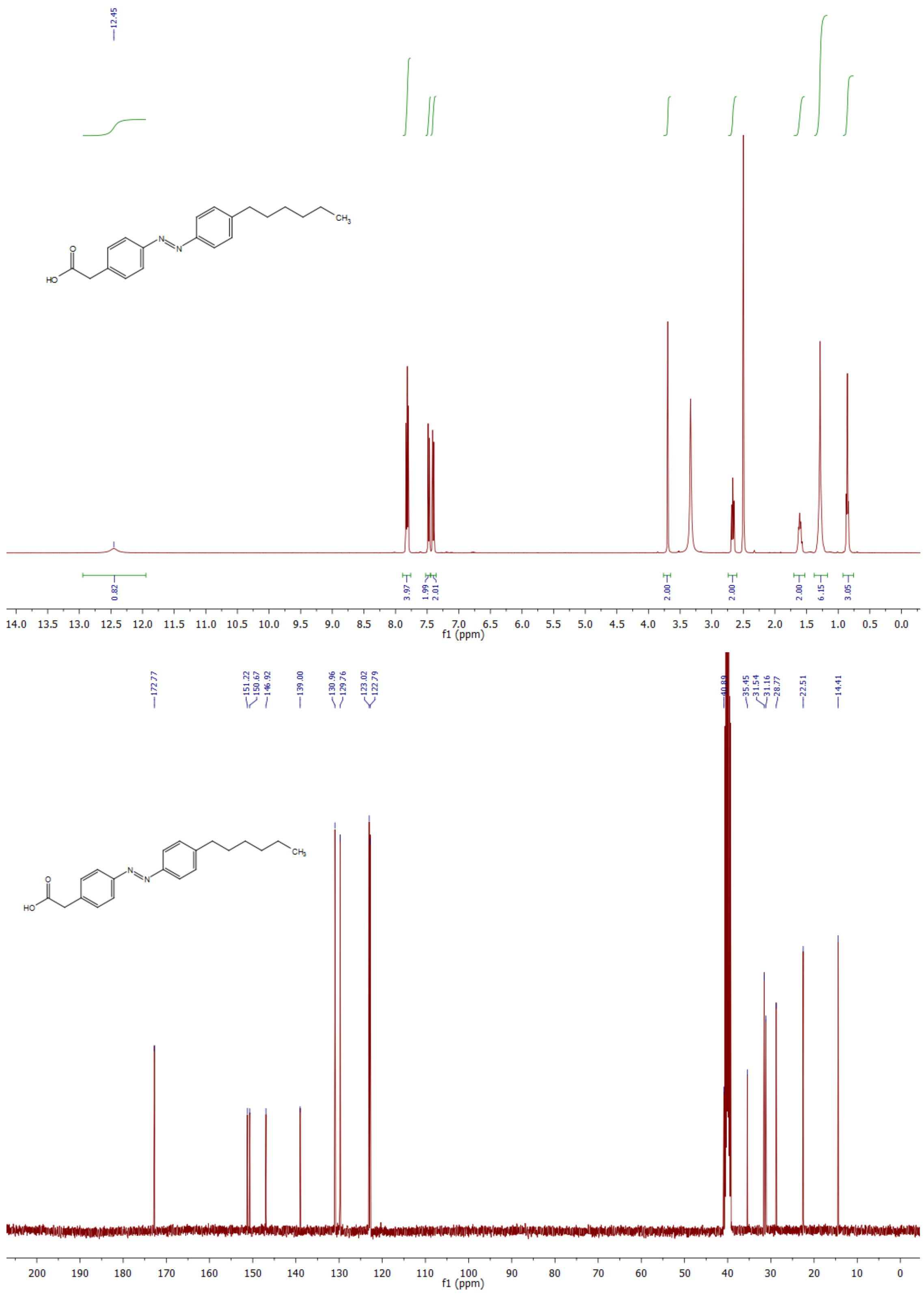

Supplementary Figure 16 | ^{1}H- and ^{13}C-NMR spectra of 2-(4-((4-hexylphenyl)diazenyl)phenyl)acetic acid (**FAAzo2**).

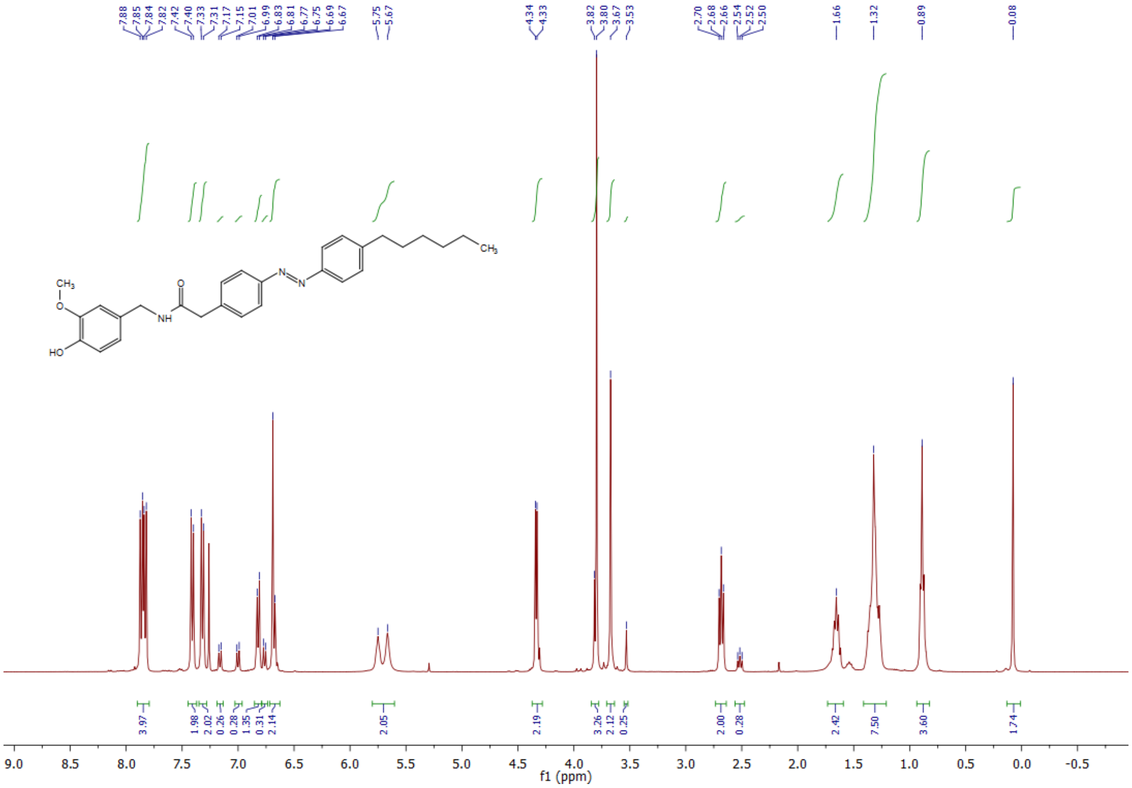

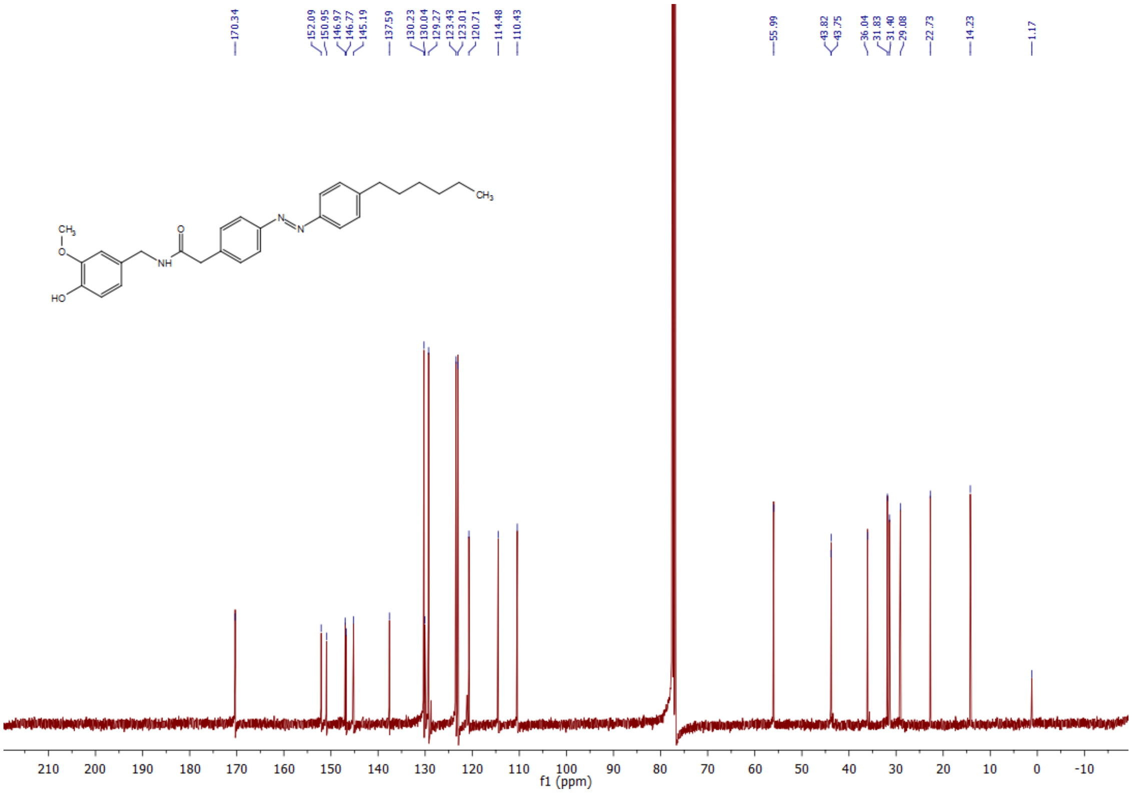

Supplementary Figure 17 | ^{1}H- and ^{13}C-NMR spectra of *N*-(4-hydroxy-3-methoxybenzyl)-2-(4-((4-hexylphenyl)diazenyl)phenyl)acetamide (**AzCA2**).

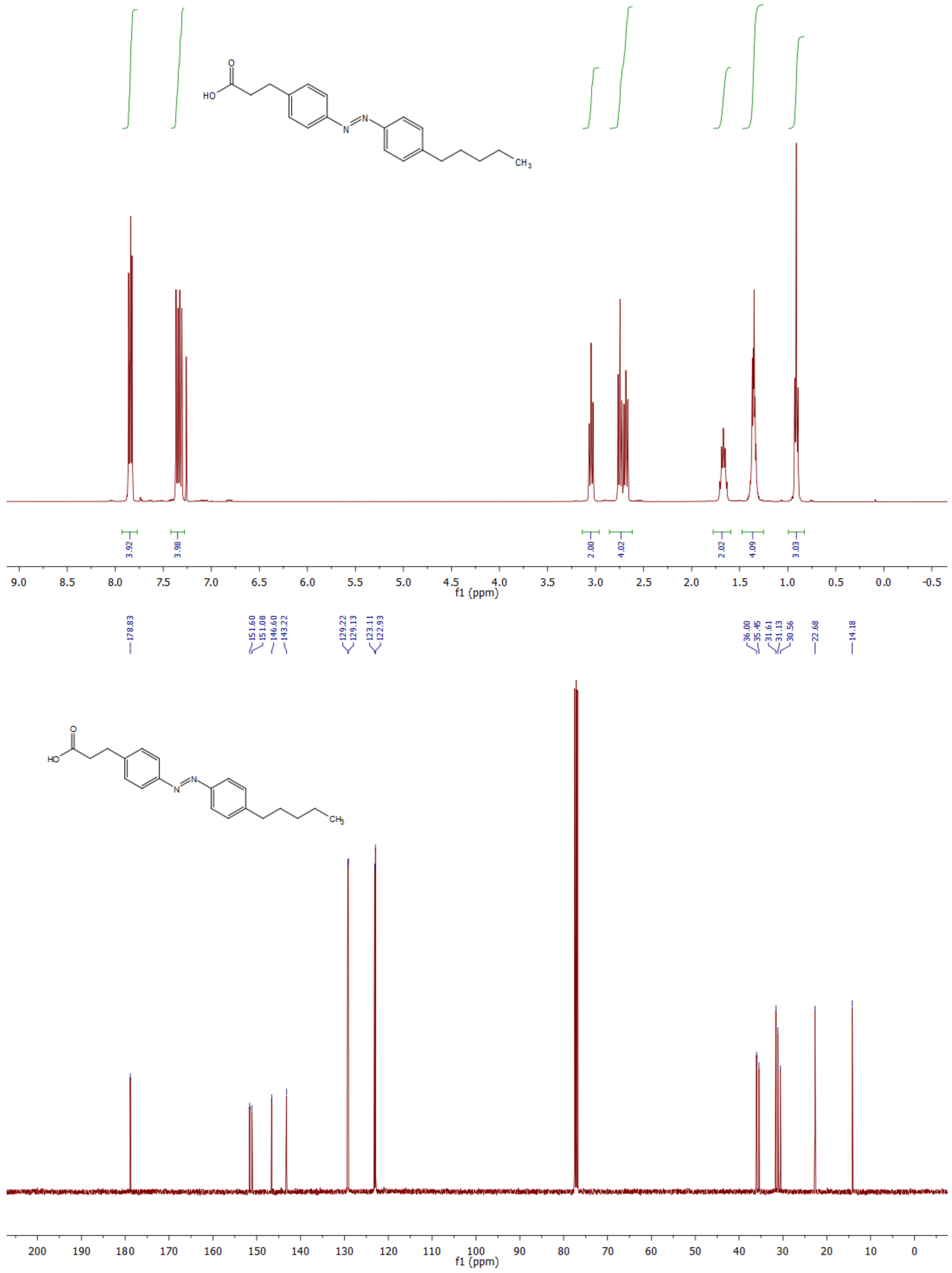

Supplementary Figure 18 | ^{1}H- and ^{13}C-NMR spectra of 3-(4-((4-pentylphenyl)diazenyl)phenyl) propanoic acid (**FAAzo3**).

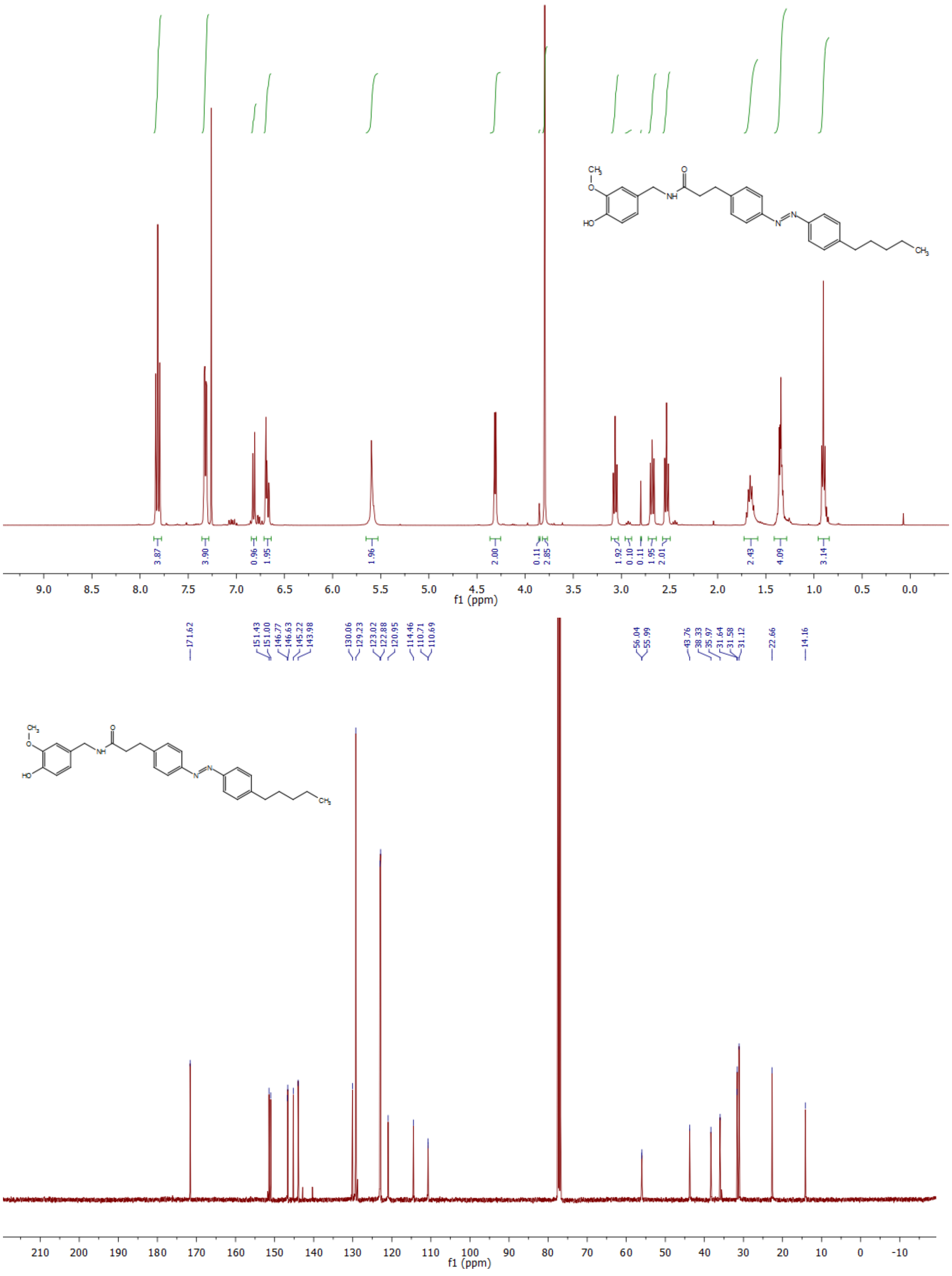

Supplementary Figure 19 | ^{1}H- and ^{13}C-NMR spectra of *N*-(4-hydroxy-3-methoxybenzyl)-3-(4-((4-pentylphenyl)diazenyl)phenyl)propanamide (**AzCA3**)

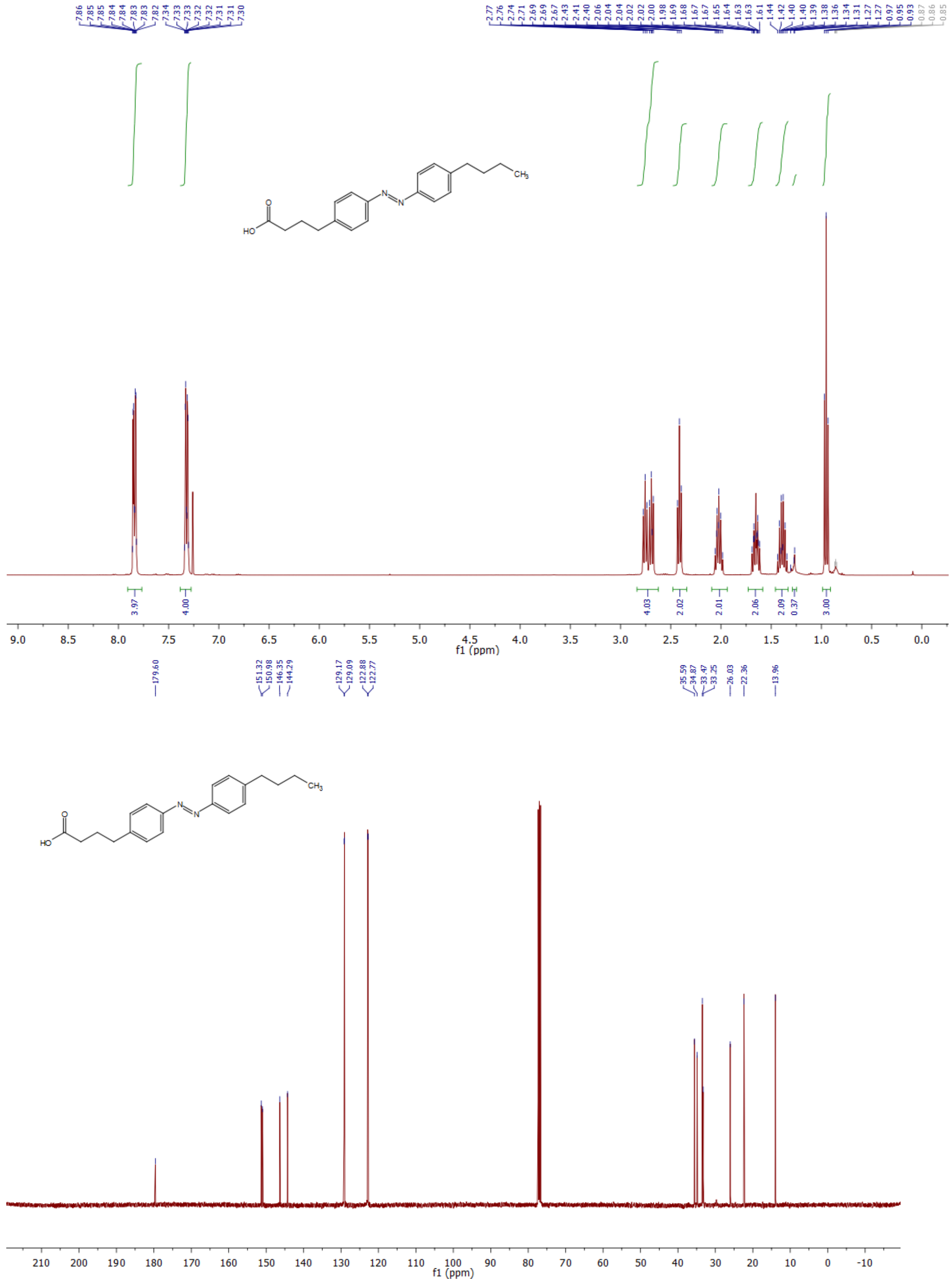

Supplementary Figure 20 | ^{1}H- and ^{13}C-NMR spectra of 4-(4-((4-butylphenyl)diazenyl)phenyl)butanoic acid (**FAAzo4**).

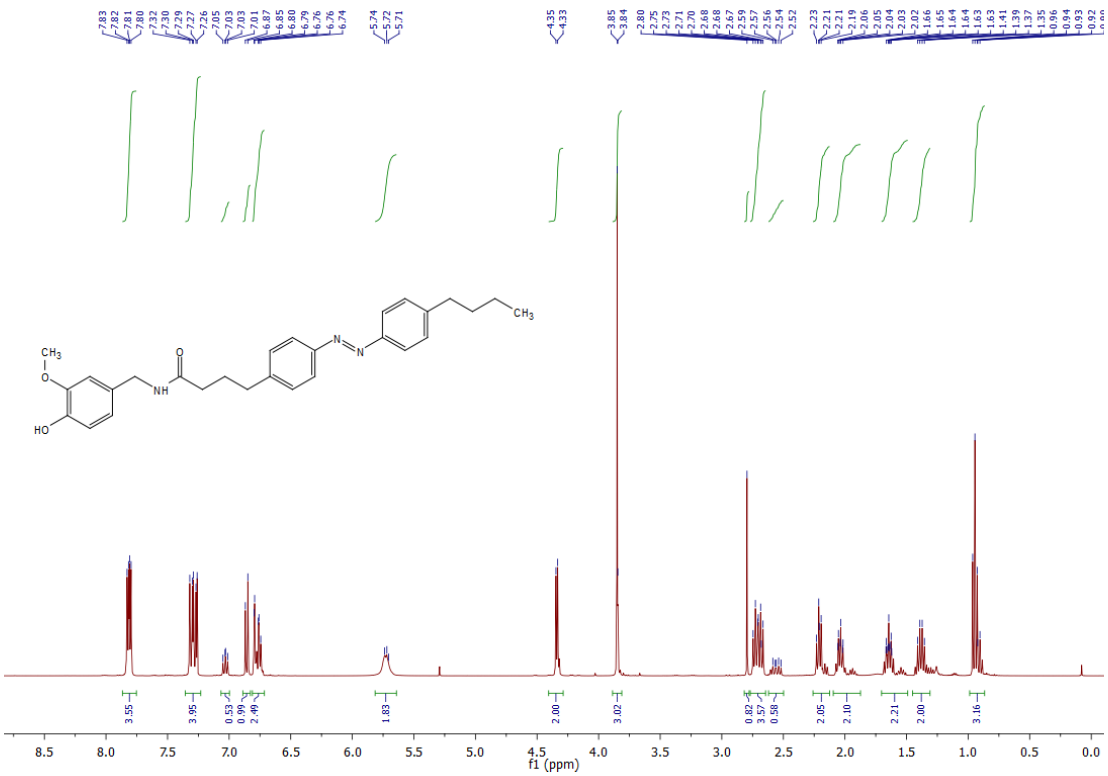

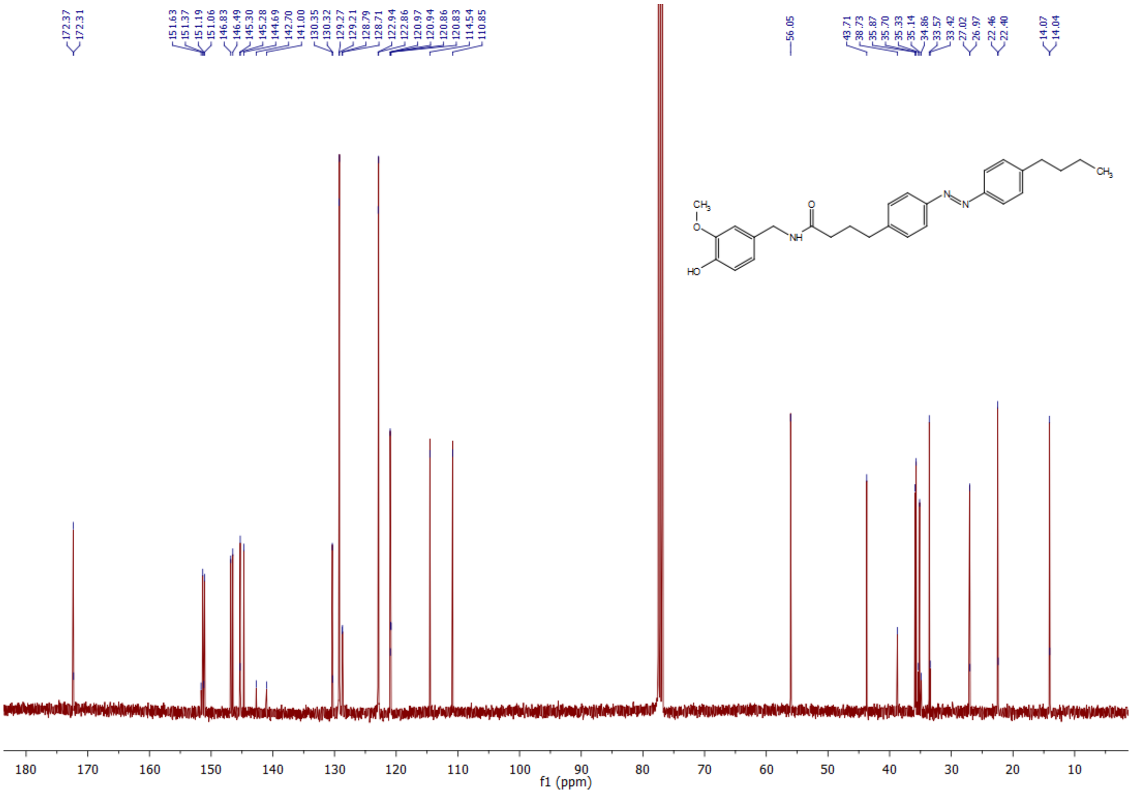

Supplementary Figure 21 | ^{1}H- and ^{13}C-NMR spectra of *N*-(4-hydroxy-3-methoxybenzyl)-4-(4-((4-butylphenyl)diazenyl)phenyl)butanamide (**AzCA4**).

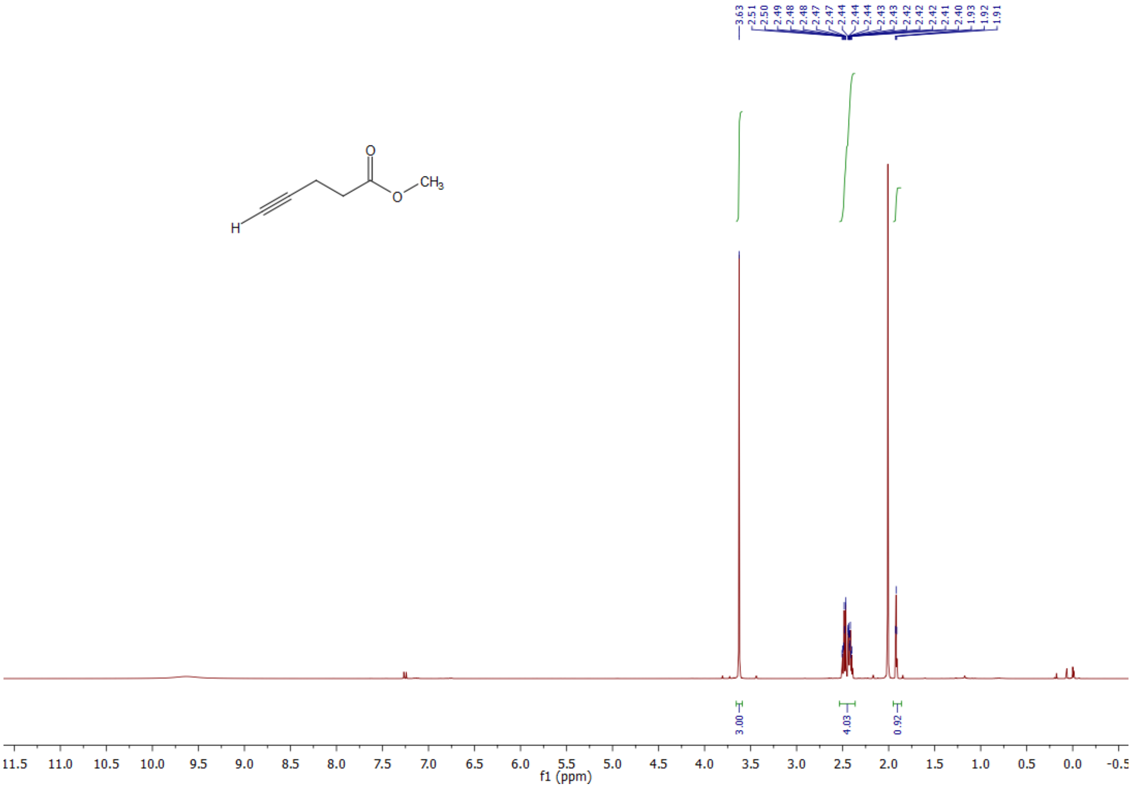

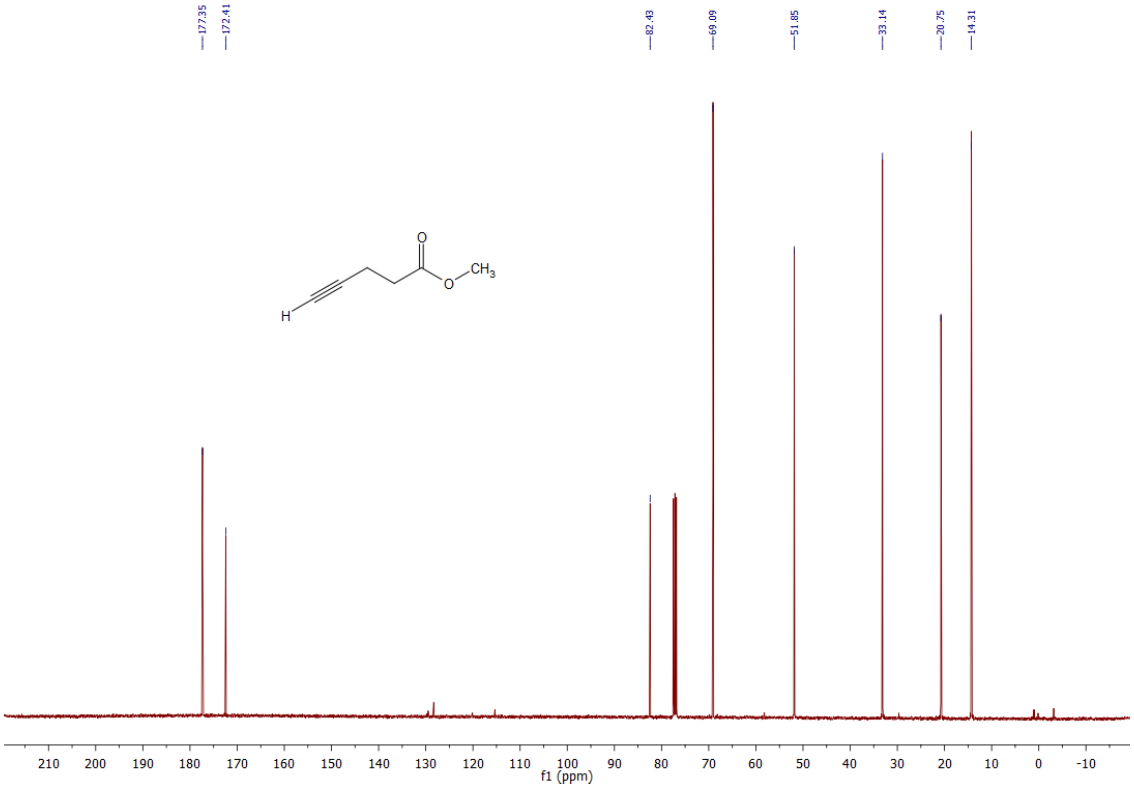

Supplementary Figure 22 | ^{1}H- and ^{13}C-NMR spectra of methyl pent-4-ynoate (**1a**).

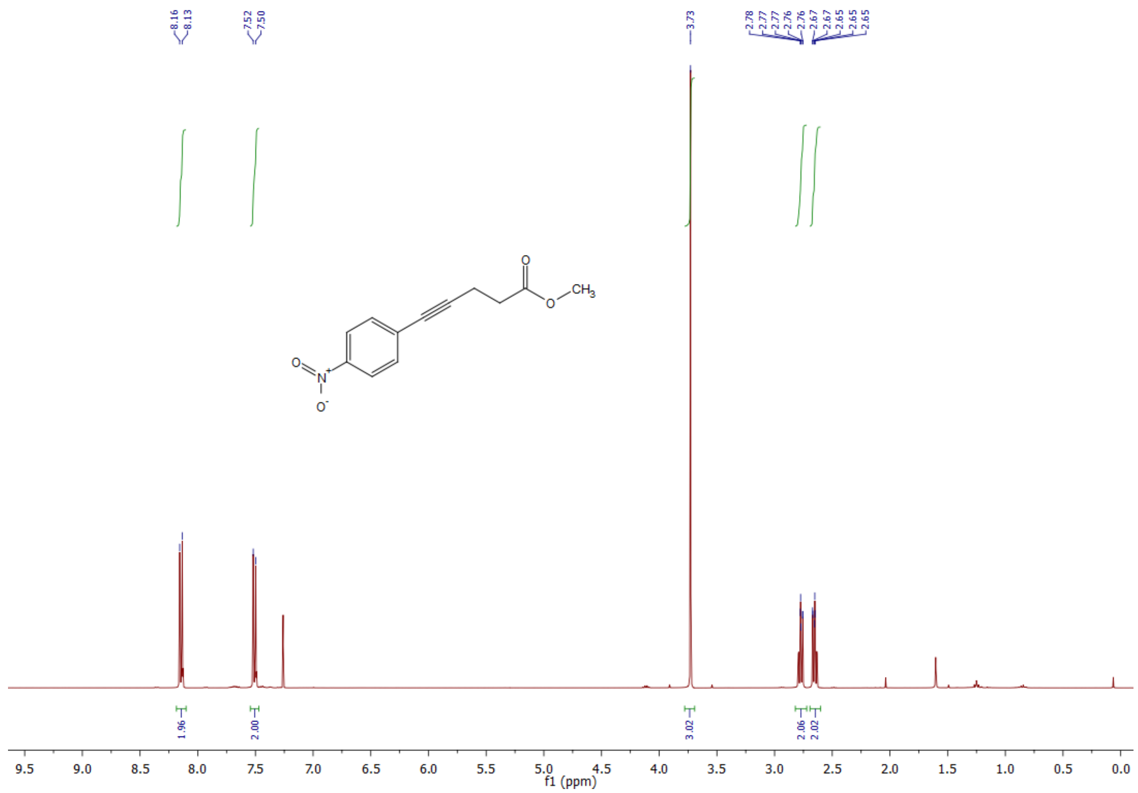

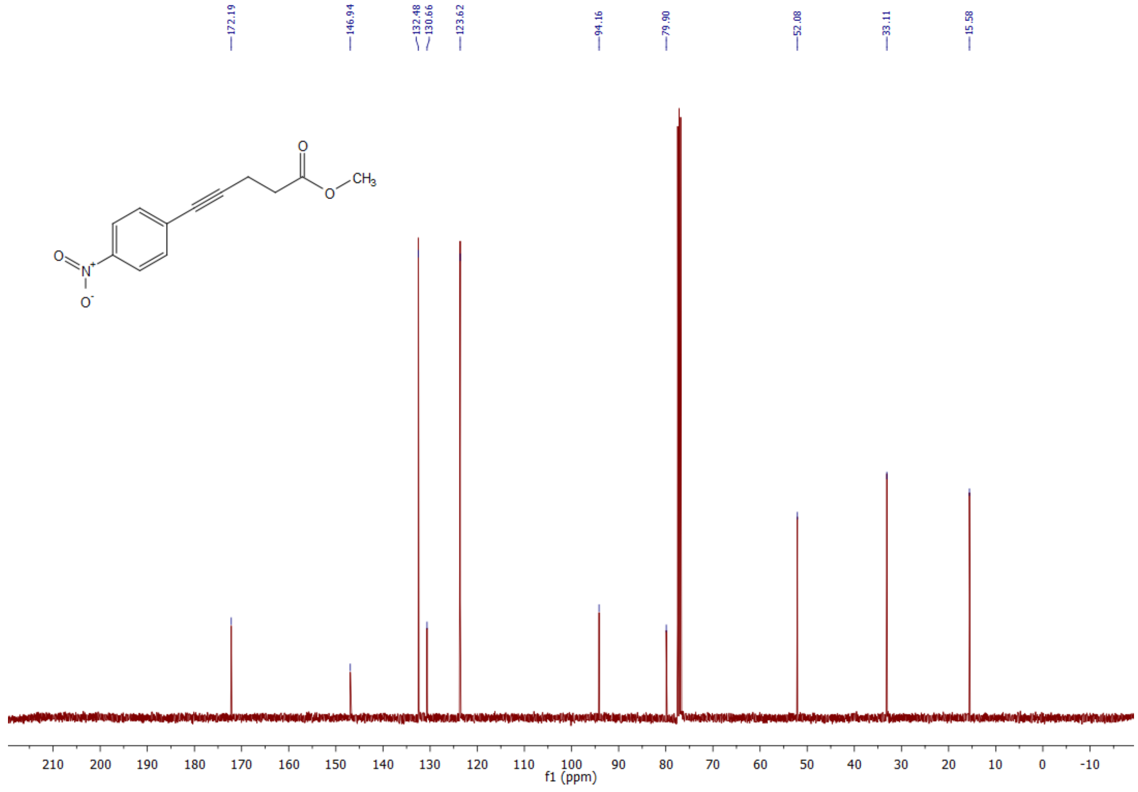

Supplementary Figure 23 | ^{1}H- and ^{13}C-NMR spectra of methyl 5-(4-nitrophenyl)pent-4-ynoate (**2a**).

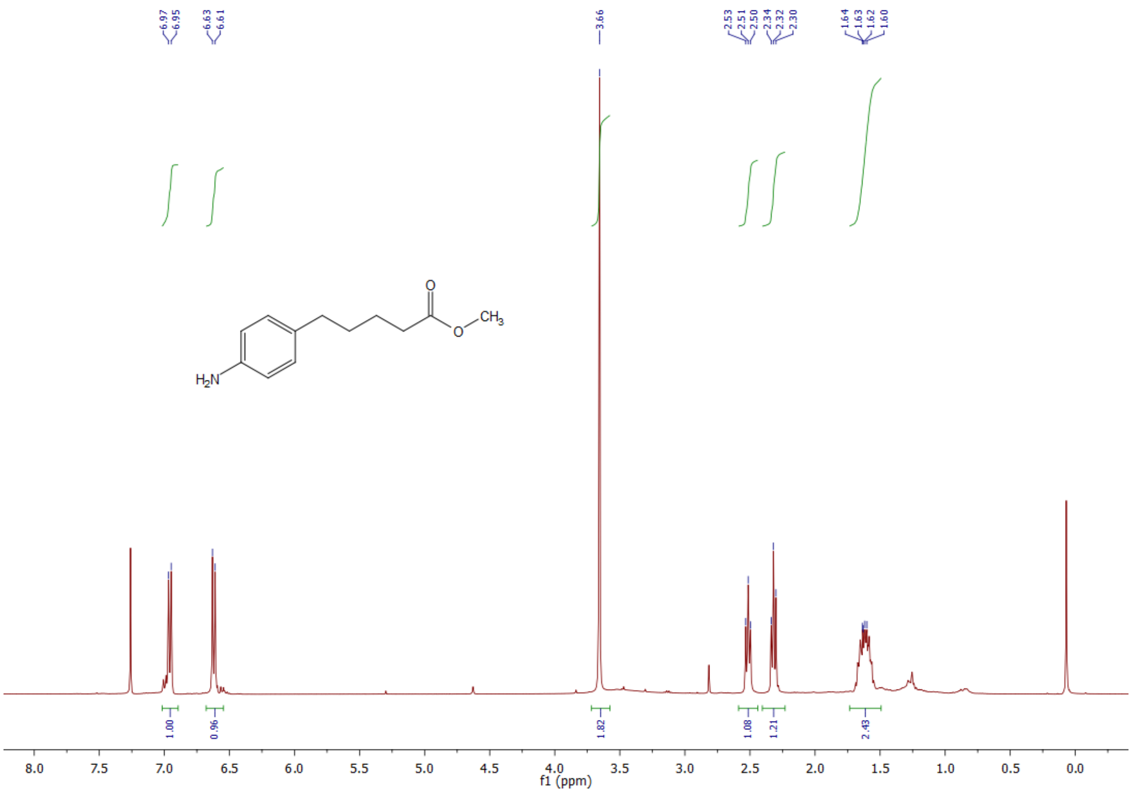

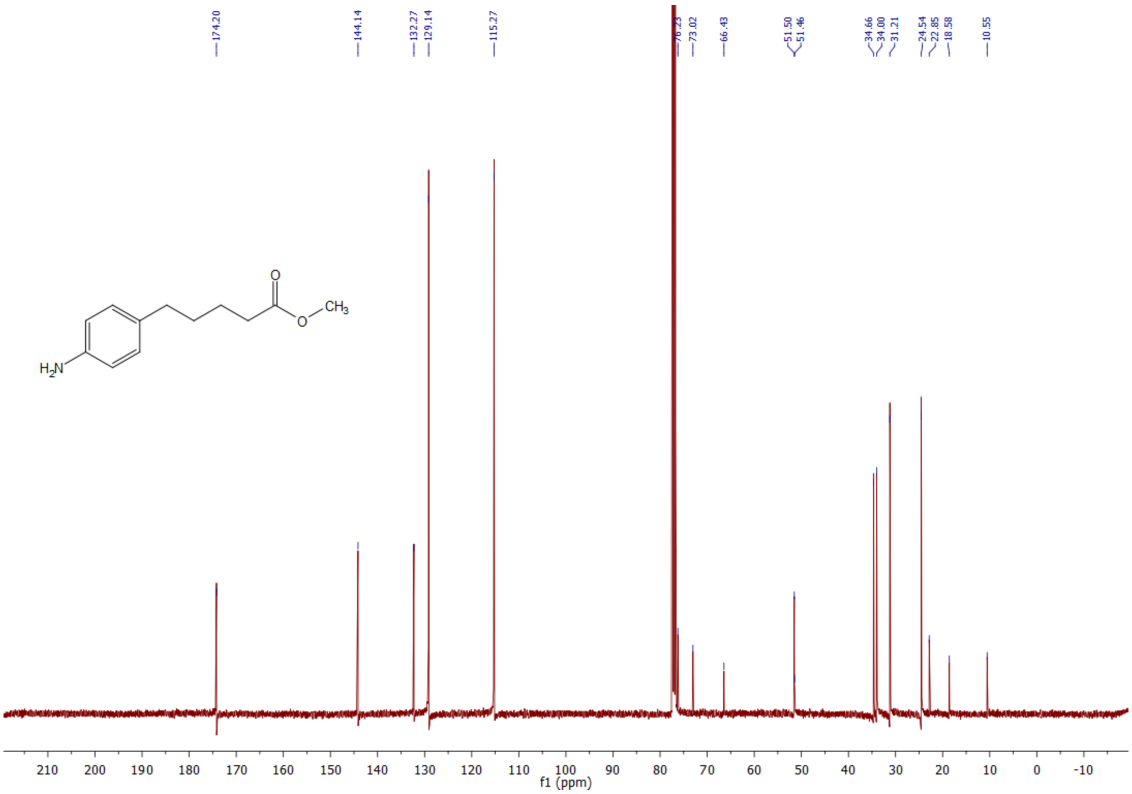

Supplementary Figure 24 | ^{1}H- and ^{13}C-NMR spectra of methyl 5-(4-aminophenyl)pentanoate (**3a**).

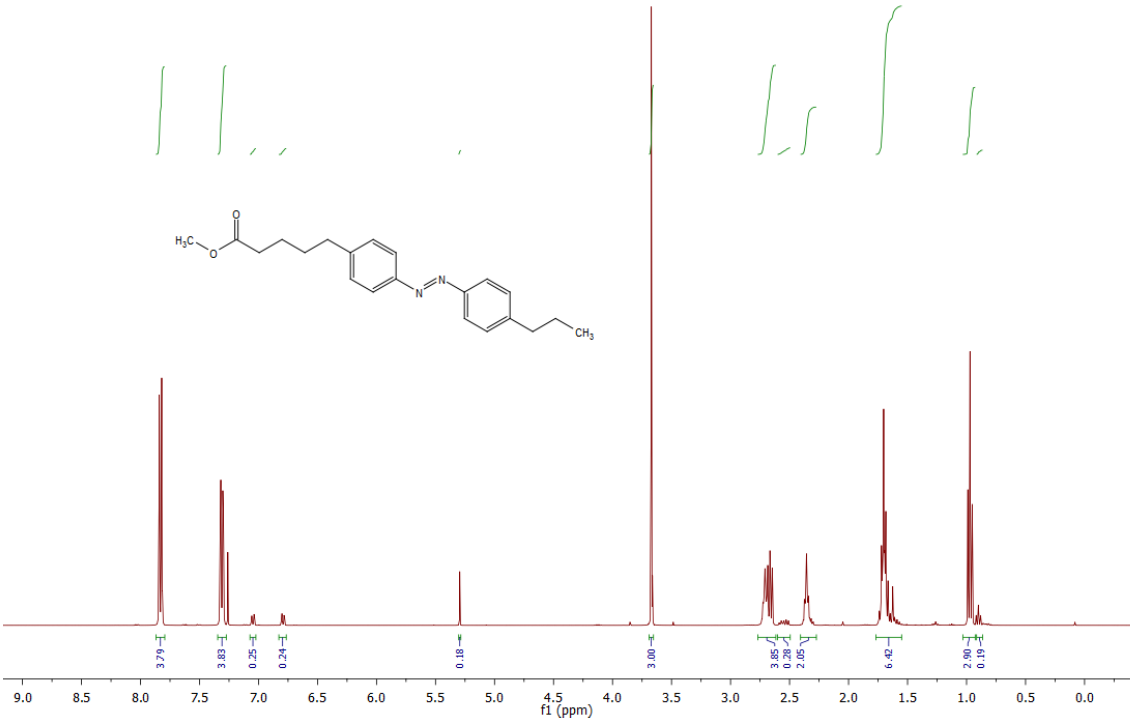

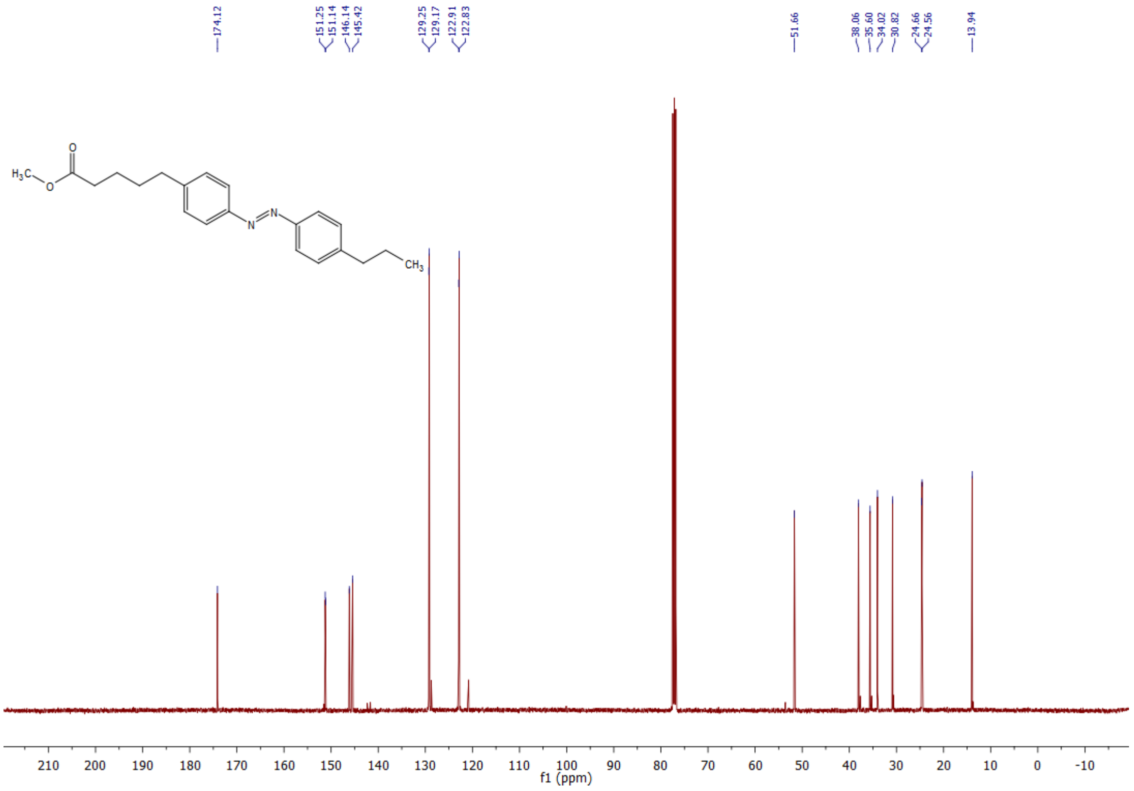

Supplementary Figure 25 | ^{1}H- and ^{13}C-NMR spectra of methyl 5-(4-((4-propylphenyl)diazenyl)phenyl) pentanoate (**4a**).

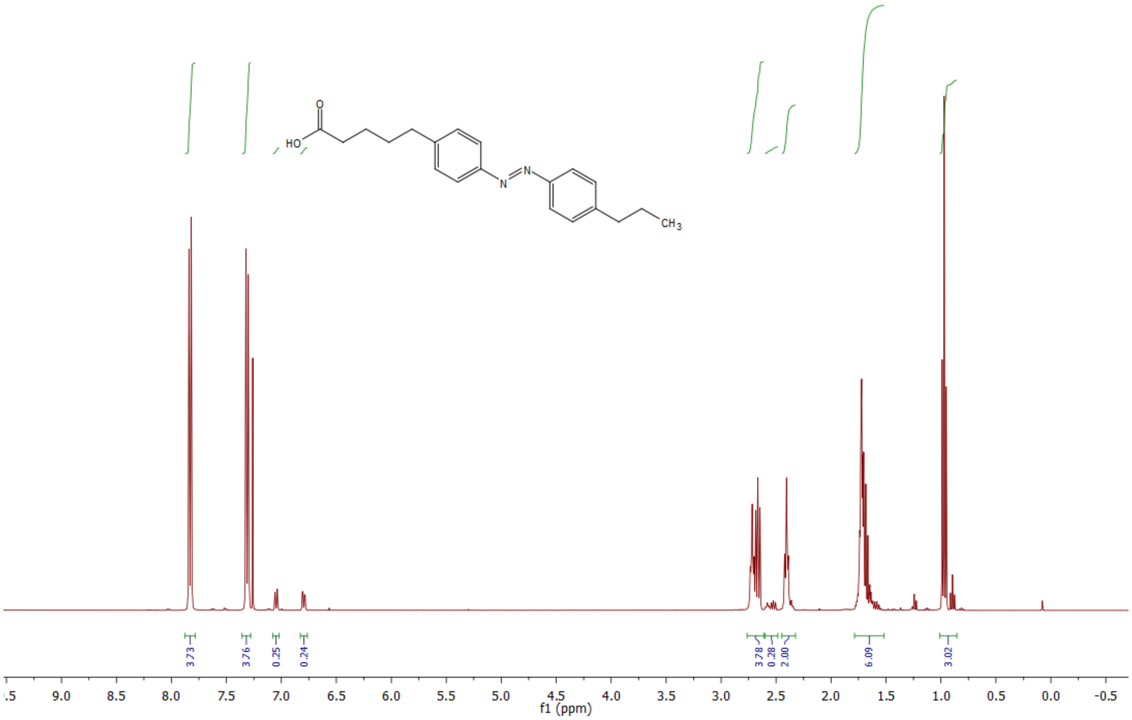

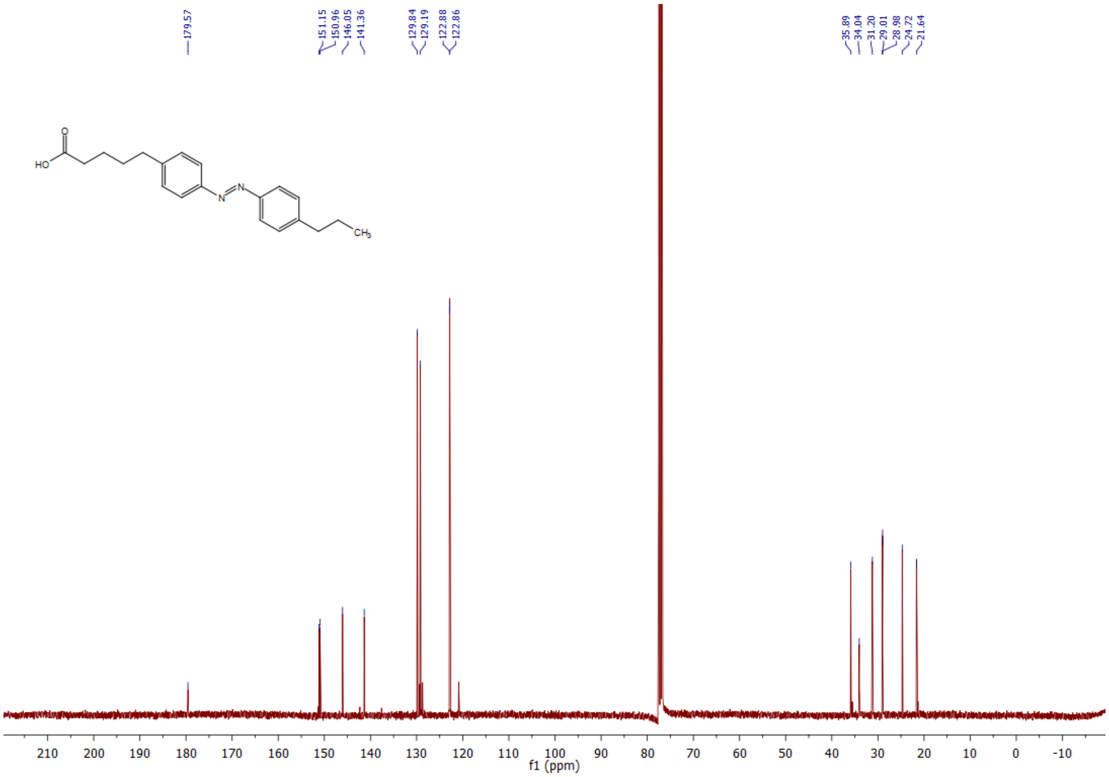

Supplementary Figure 26 | ^{1}H- and ^{13}C-NMR spectra of 5-(4-((4-propylphenyl)diazenyl)phenyl) pentanoic acid (**FAAzo5**).

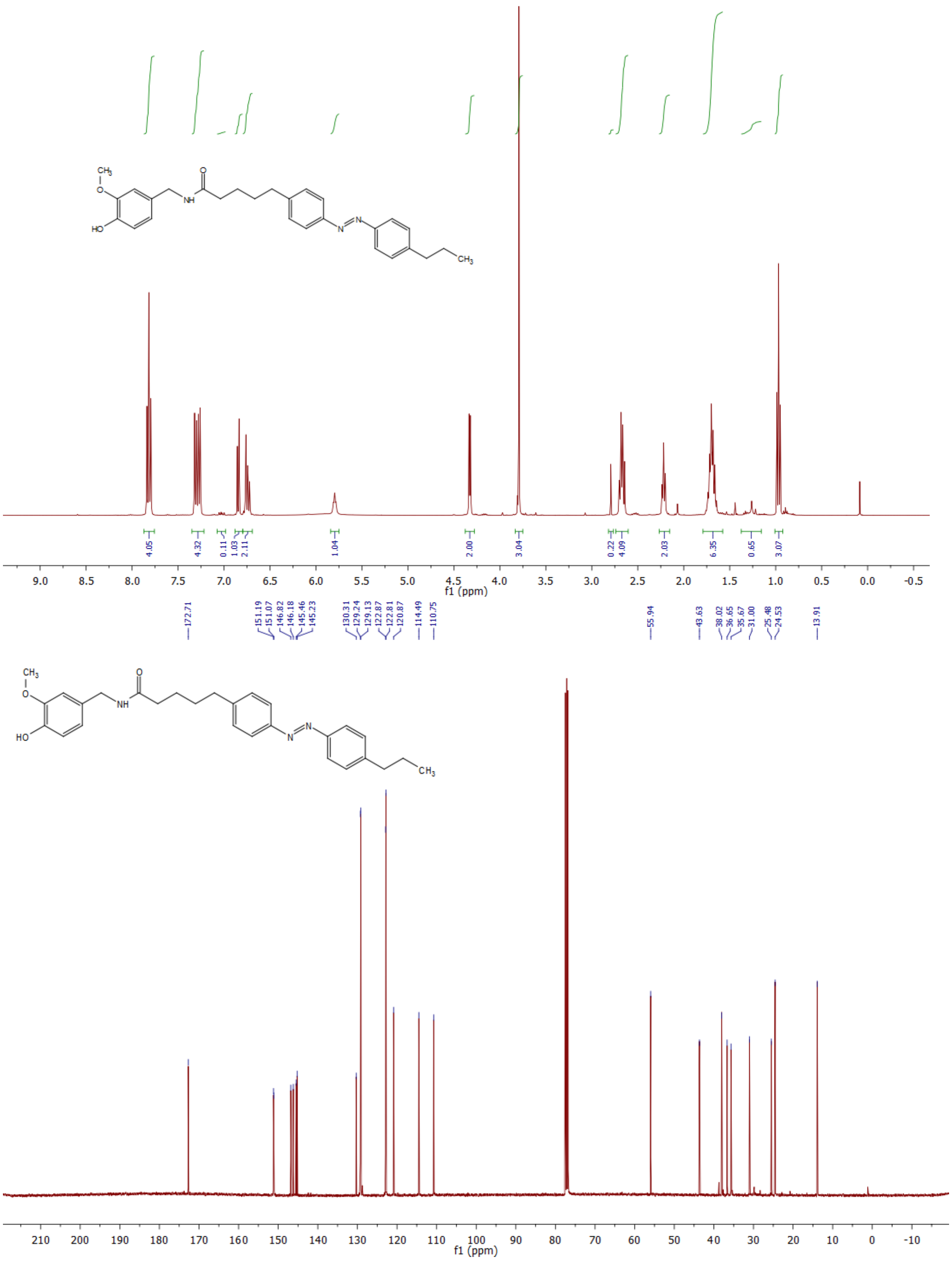

Supplementary Figure 27 | ^{1}H- and ^{13}C-NMR spectra of *N*-(4-hydroxy-3-methoxybenzyl)-5-(4-((4-propylphenyl)diazenyl)phenyl)pentanamide (**AzCA5**).

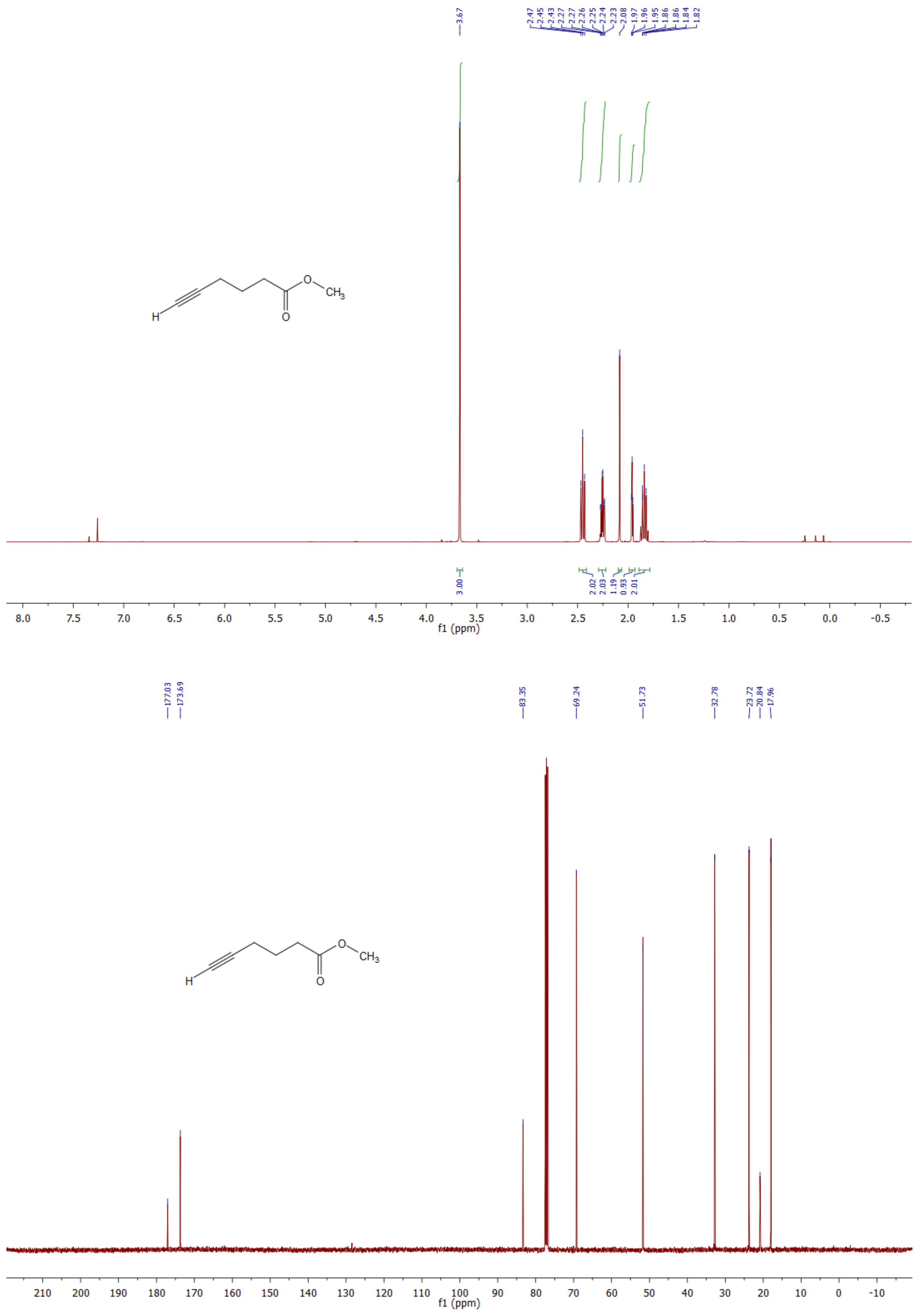

Supplementary Figure 28 | ^{1}H- and ^{13}C-NMR spectra of methyl hex-5-ynoate (**1b**).

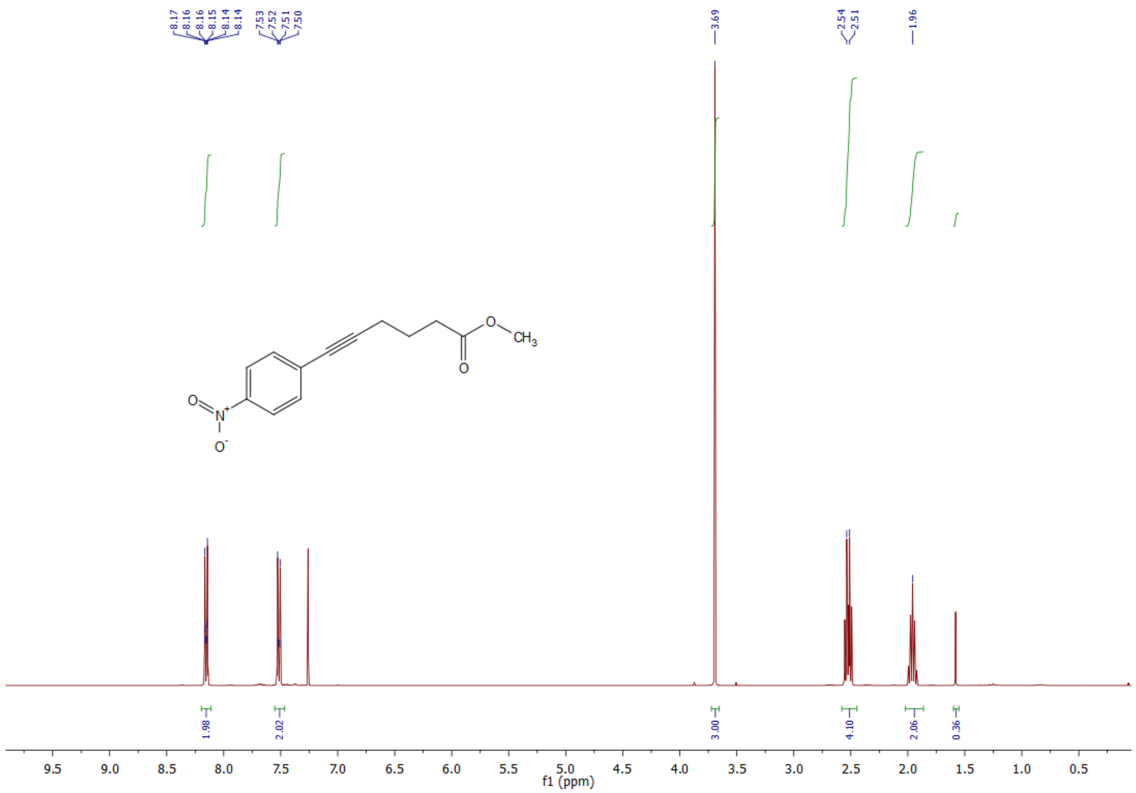

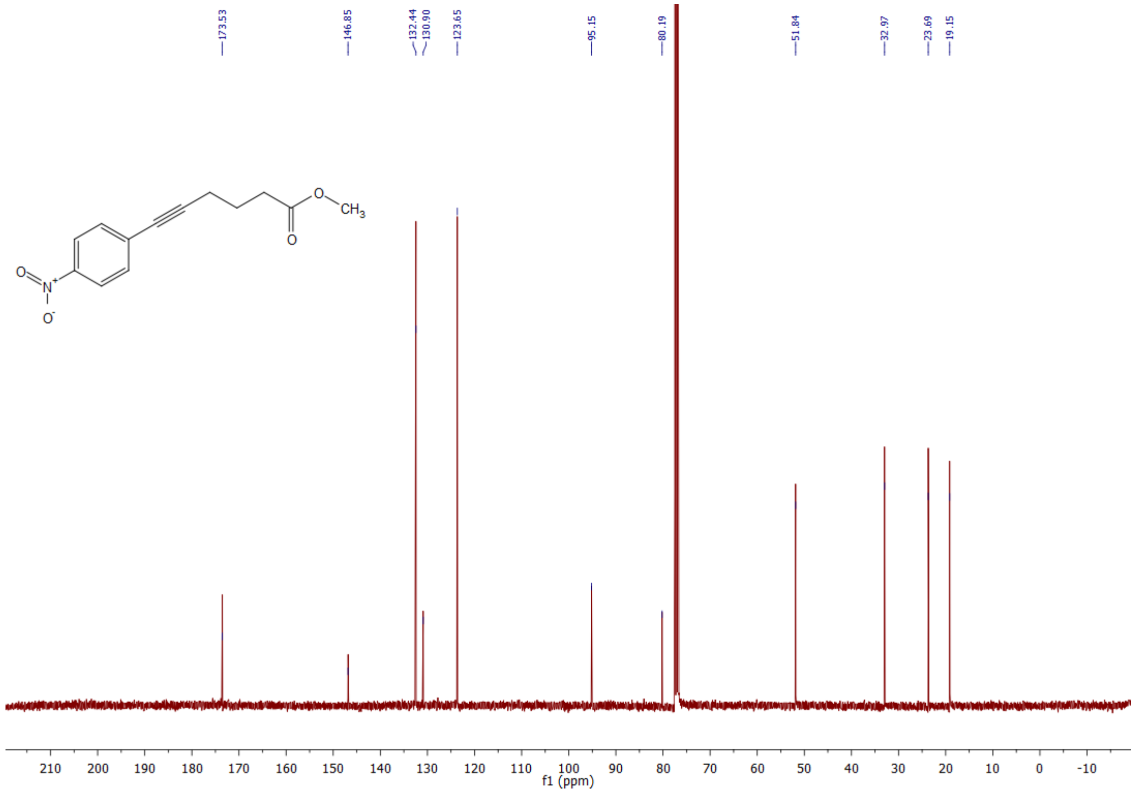

Supplementary Figure 29 | ^{1}H- and ^{13}C-NMR spectra of methyl 6-(4-nitrophenyl)hex-5-ynoate (**2b**).

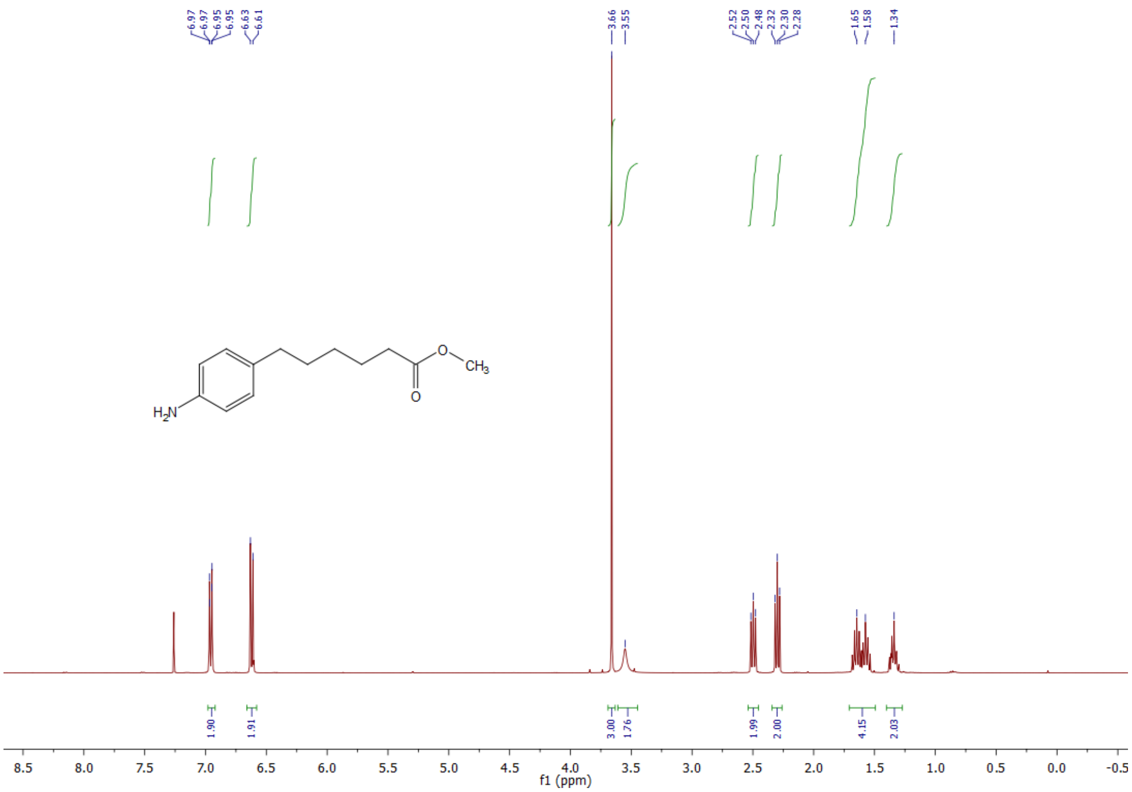

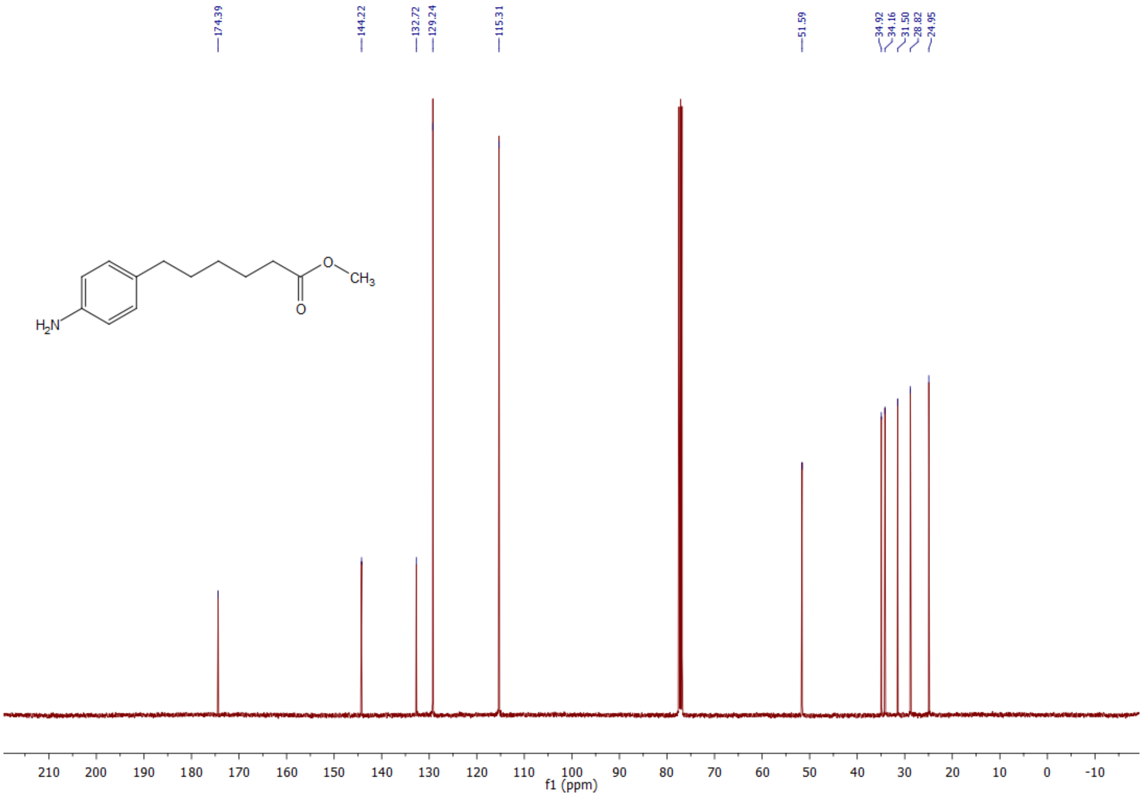

Supplementary Figure 30 | ^{1}H- and ^{13}C-NMR spectra of methyl 6-(4-aminophenyl)hexanoate (**3b**).

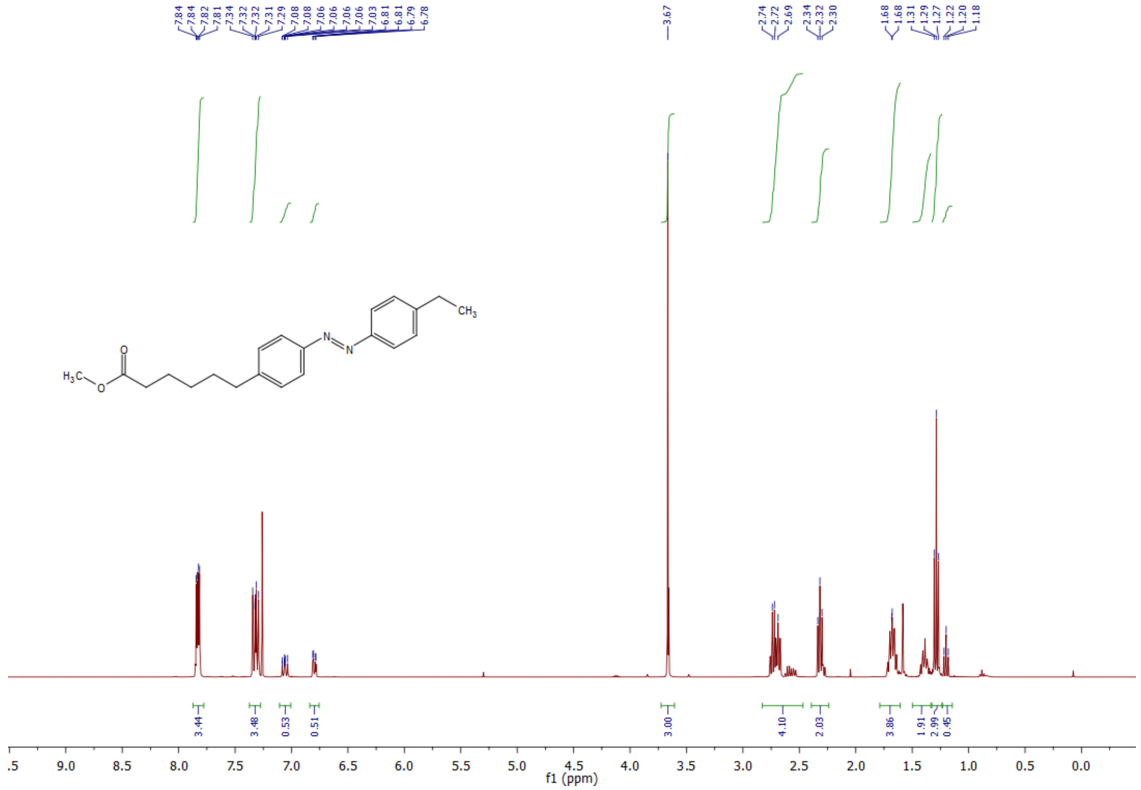

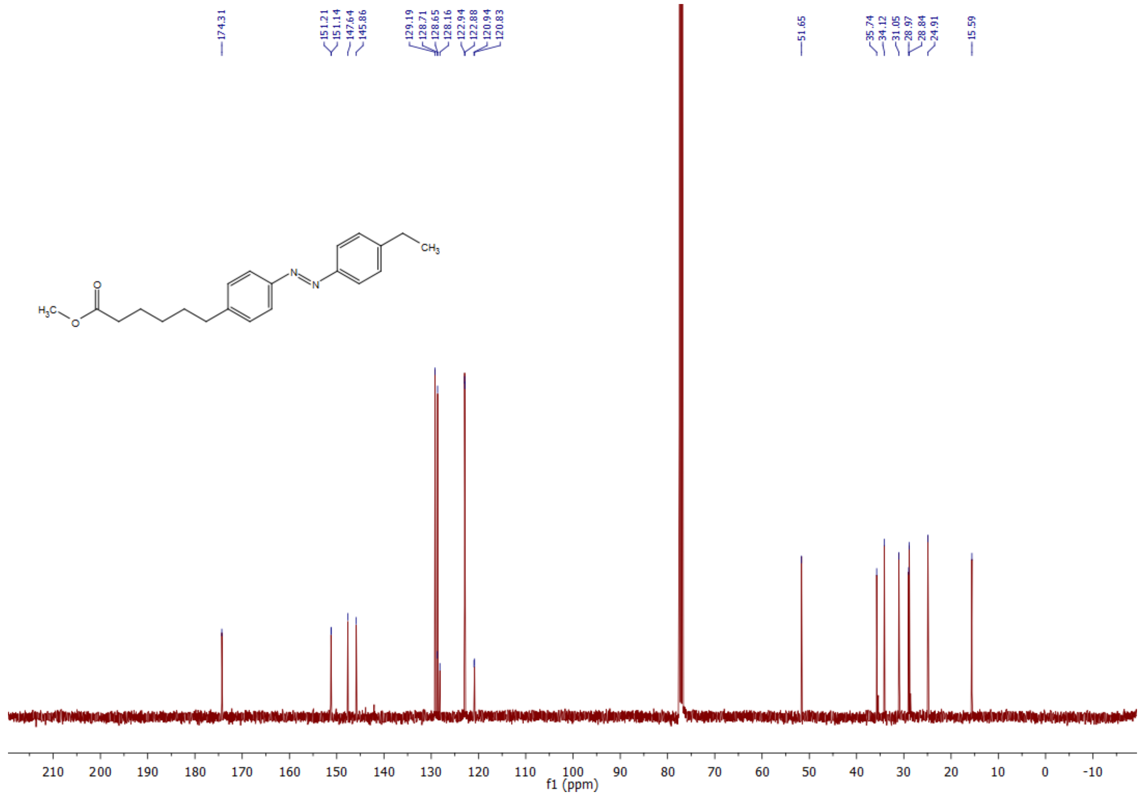

Supplementary Figure 31 | ^{1}H- and ^{13}C-NMR spectra of methyl 6-(4-((4-ethylphenyl)diazenyl)phenyl) hexanoate (**4b**).

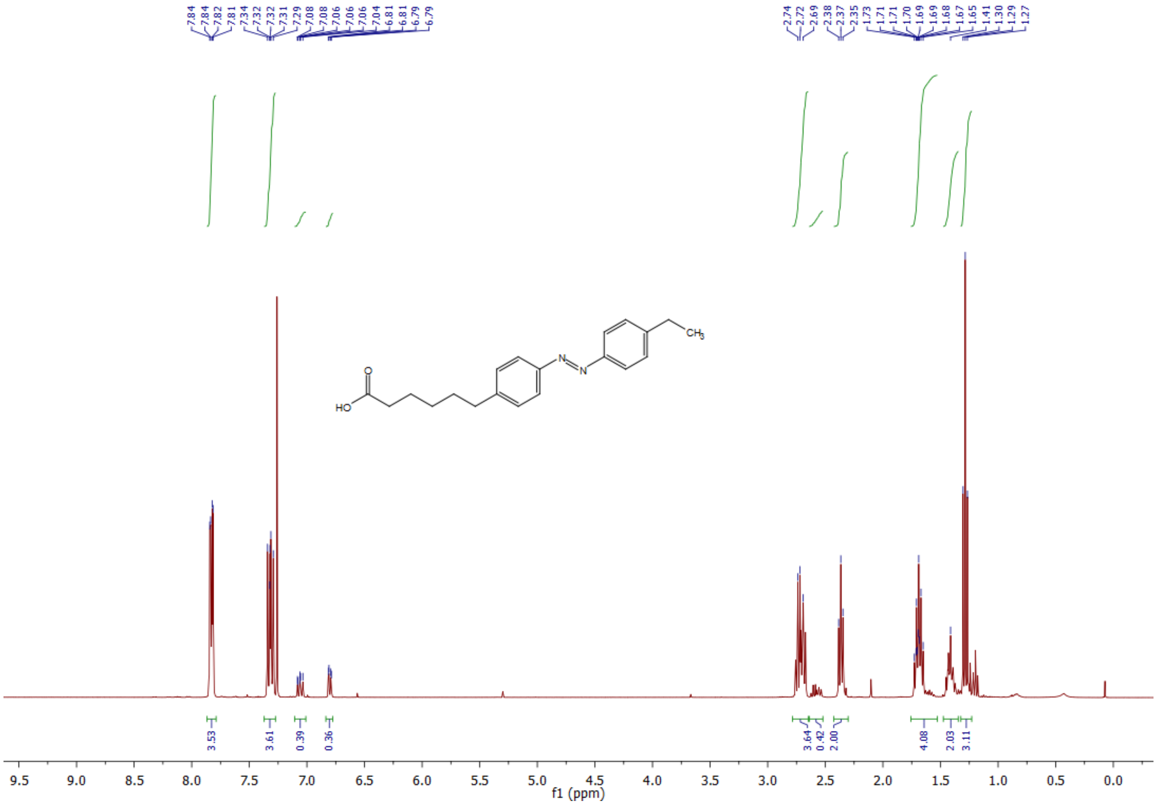

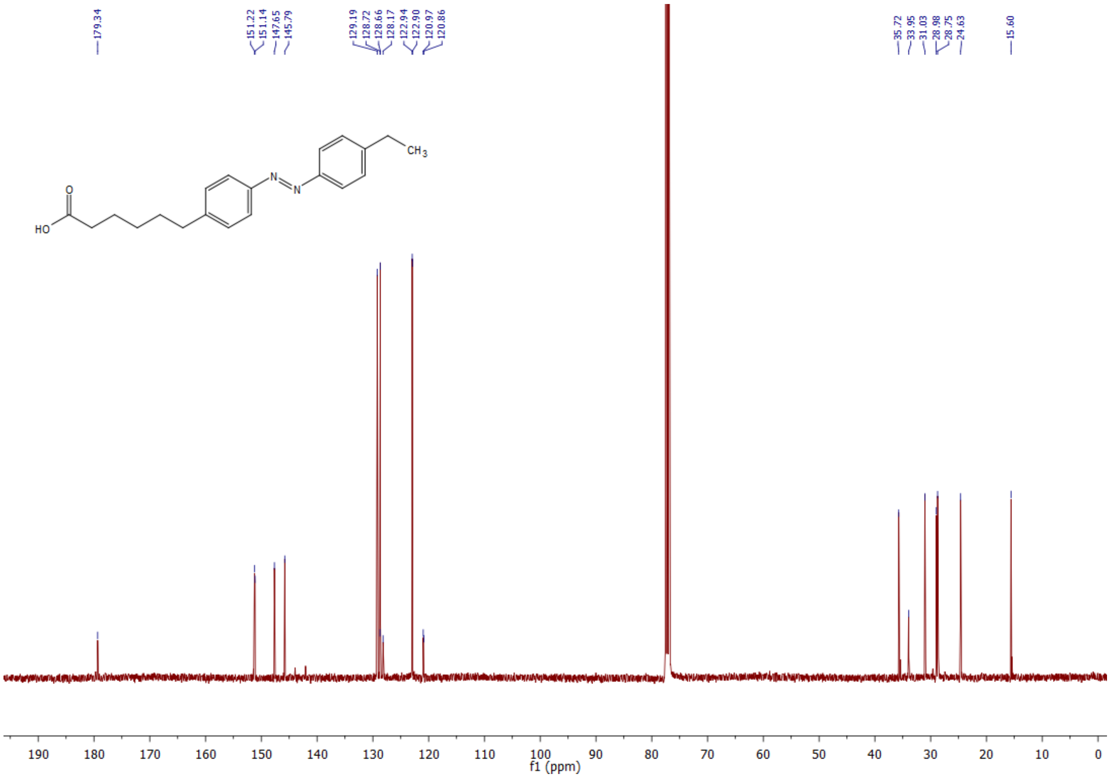

Supplementary Figure 32 | ^{1}H- and ^{13}C-NMR spectra of 6-(4-((4-ethylphenyl)diazenyl)phenyl) hexanoic acid (**FAAzo6**).

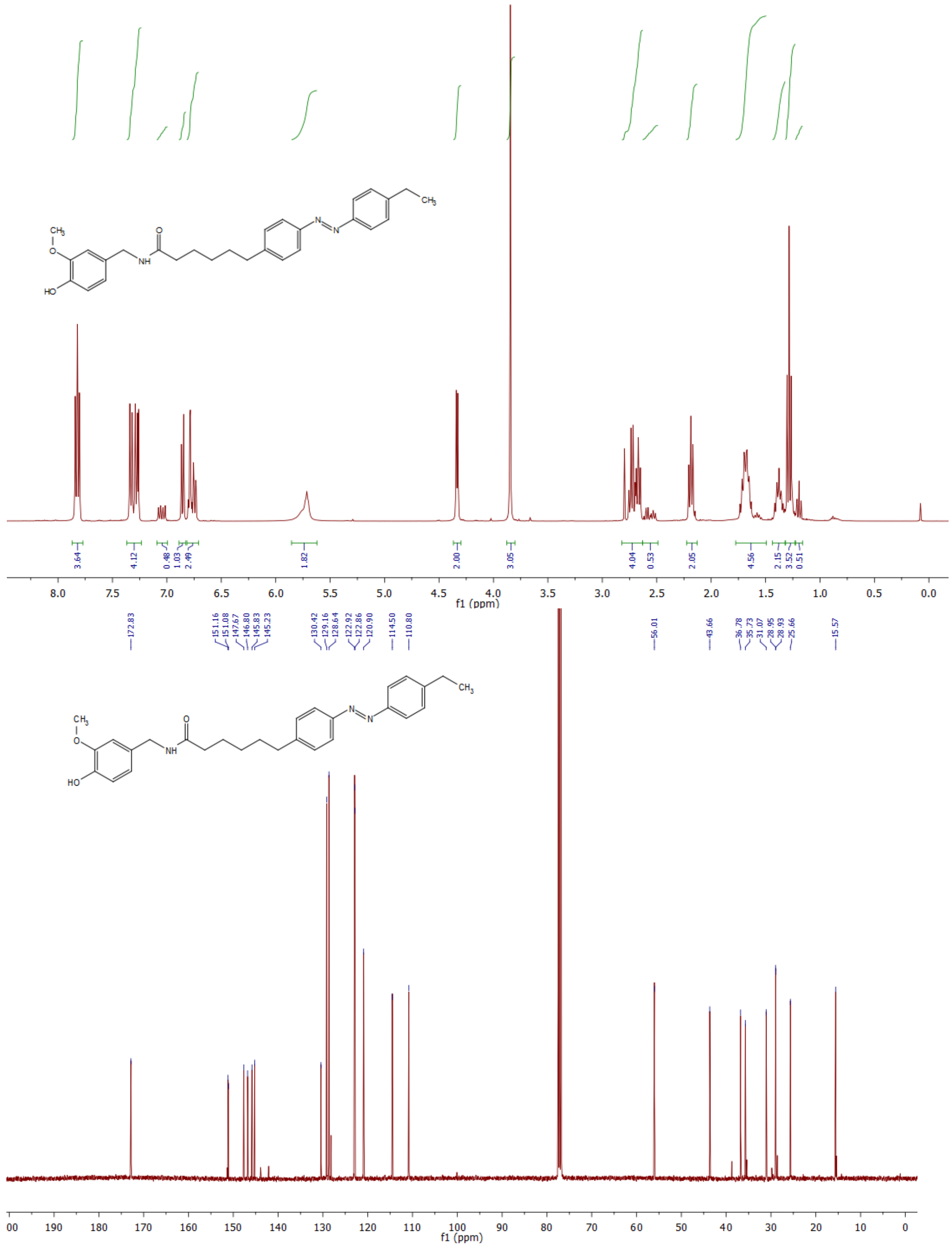

Supplementary Figure 33 | ^{1}H- and ^{13}C-NMR spectra of *N*-(4-hydroxy-3-methoxybenzyl)-6-(4-((4-ethylphenyl)diazenyl)phenyl)hexanamide (**AzCA6**).

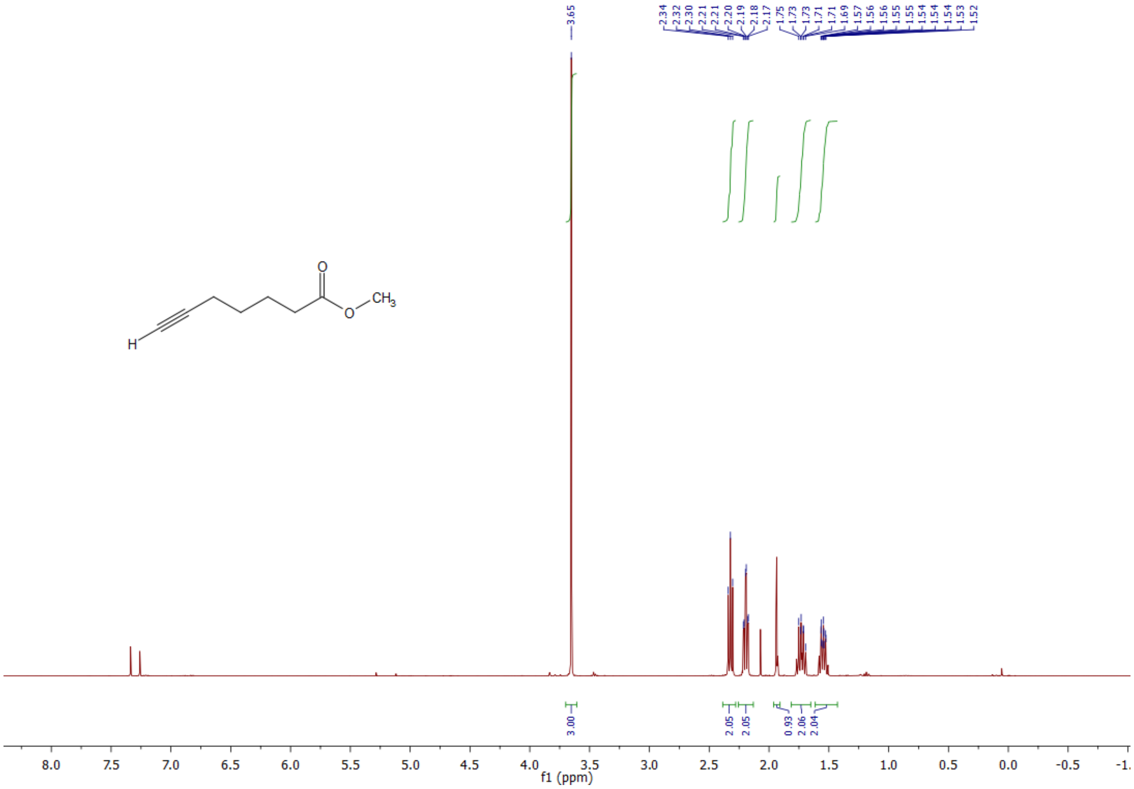

Supplementary Figure 34 | ^{1}H-NMR spectra of methyl hept-6-ynoate (**1c**).

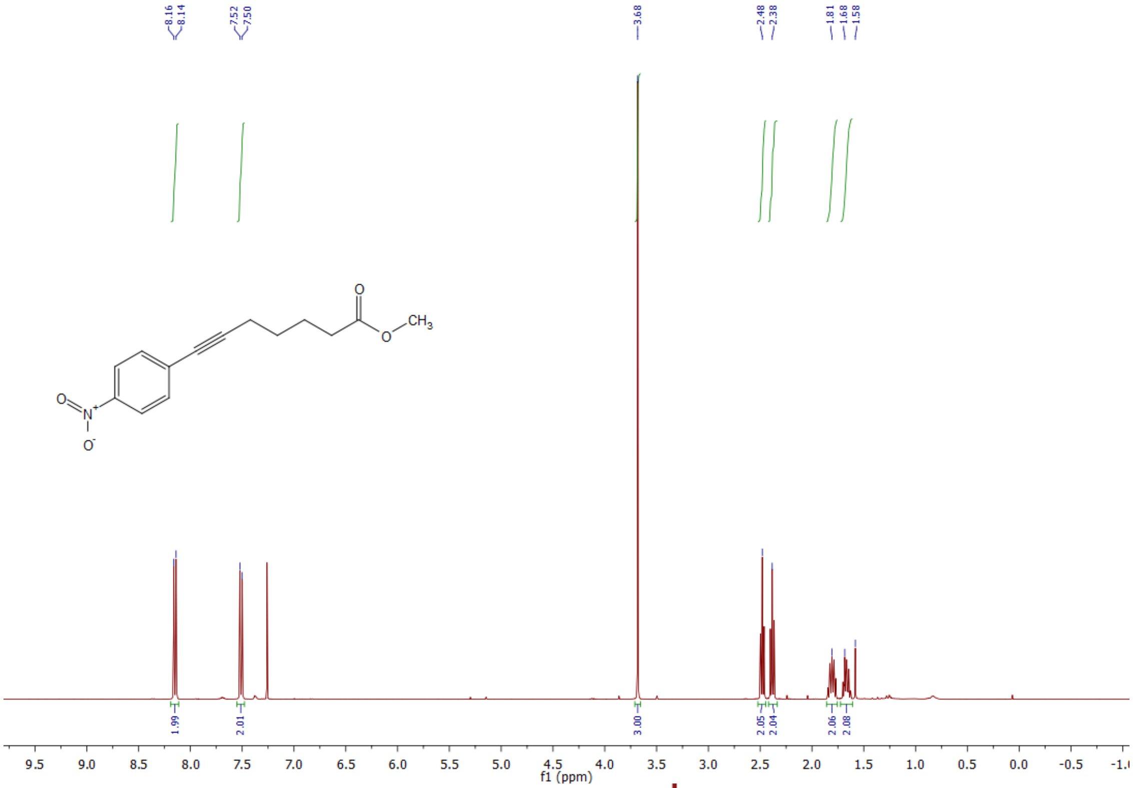

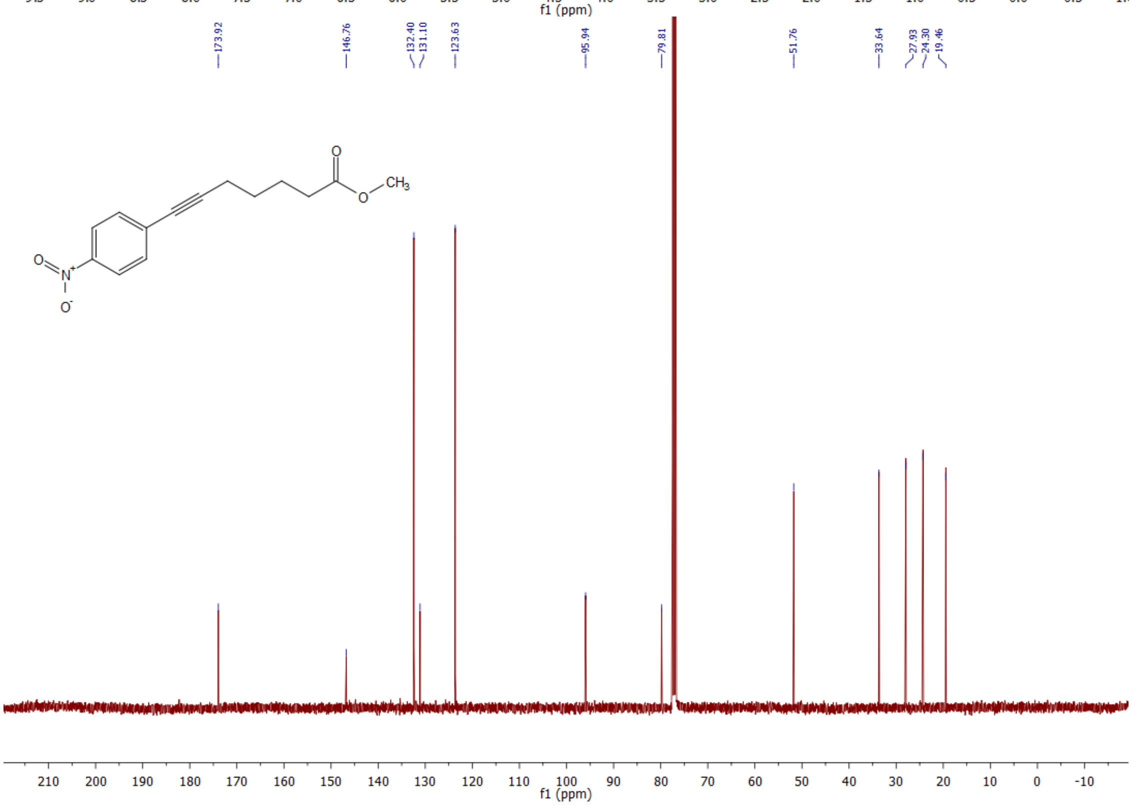

Supplementary Figure 35 | ^{1}H- and ^{13}C-NMR spectra of methyl 7-(4-nitrophenyl)hept-6-ynoate (**2c**).

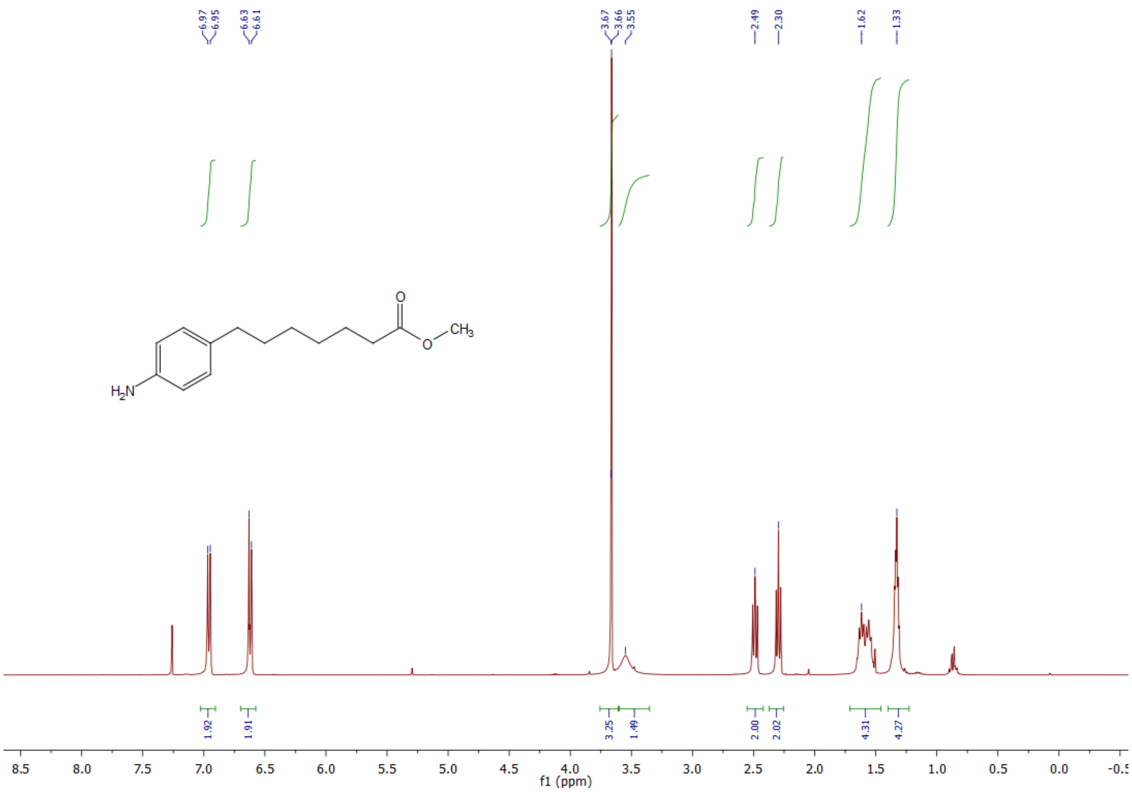

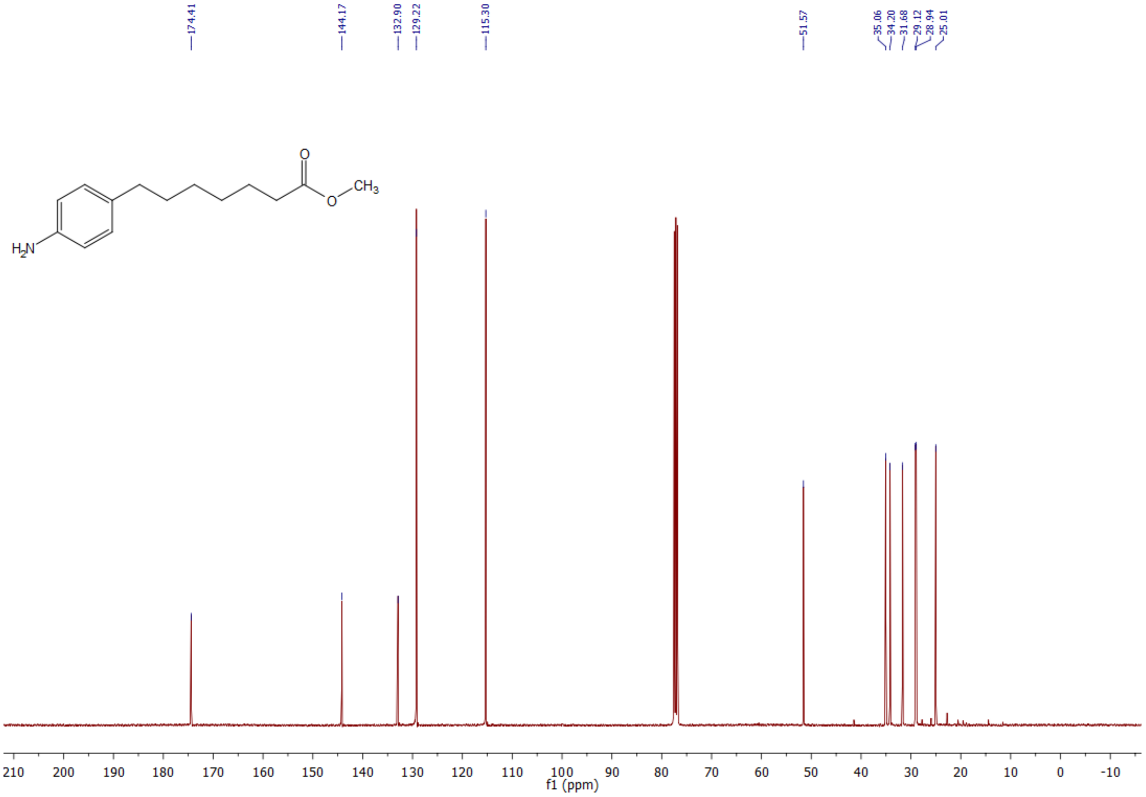

Supplementary Figure 36 | ^{1}H- and ^{13}C-NMR spectra of methyl 7-(4-aminophenyl)heptanoate (**3c**).

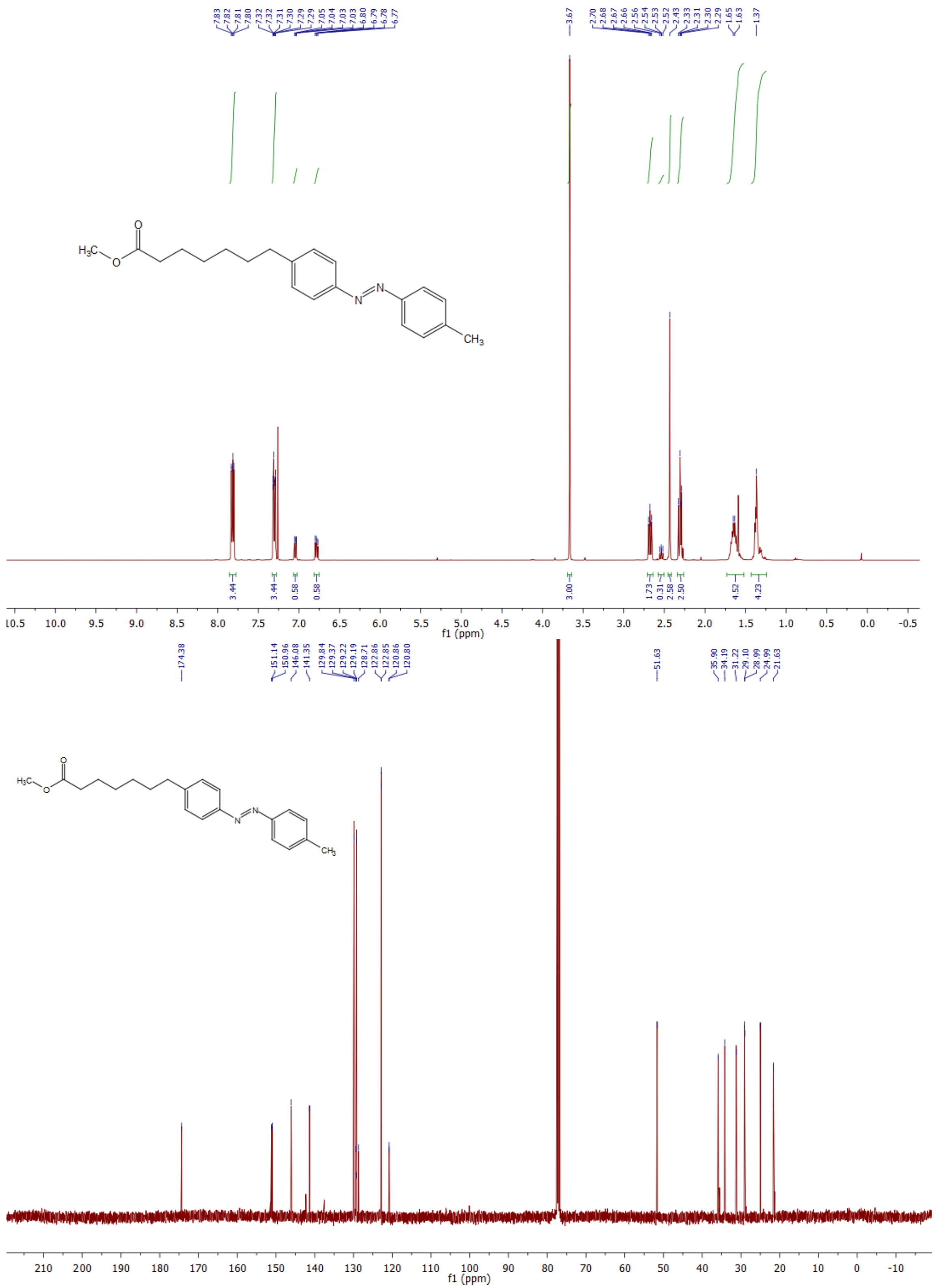

Supplementary Figure 37 | ^{1}H- and ^{13}C-NMR spectra of Methyl 7-(4-(*p*-tolyldiazenyl)phenyl)heptanoate (**4c**).

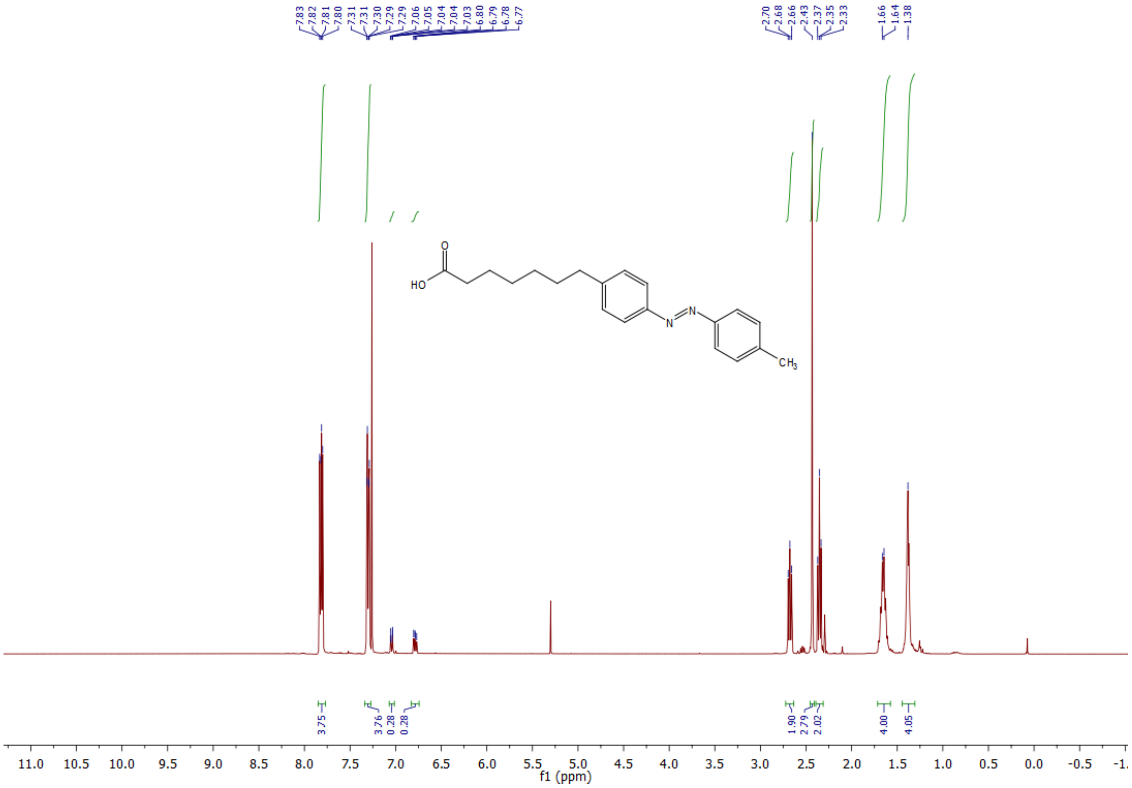

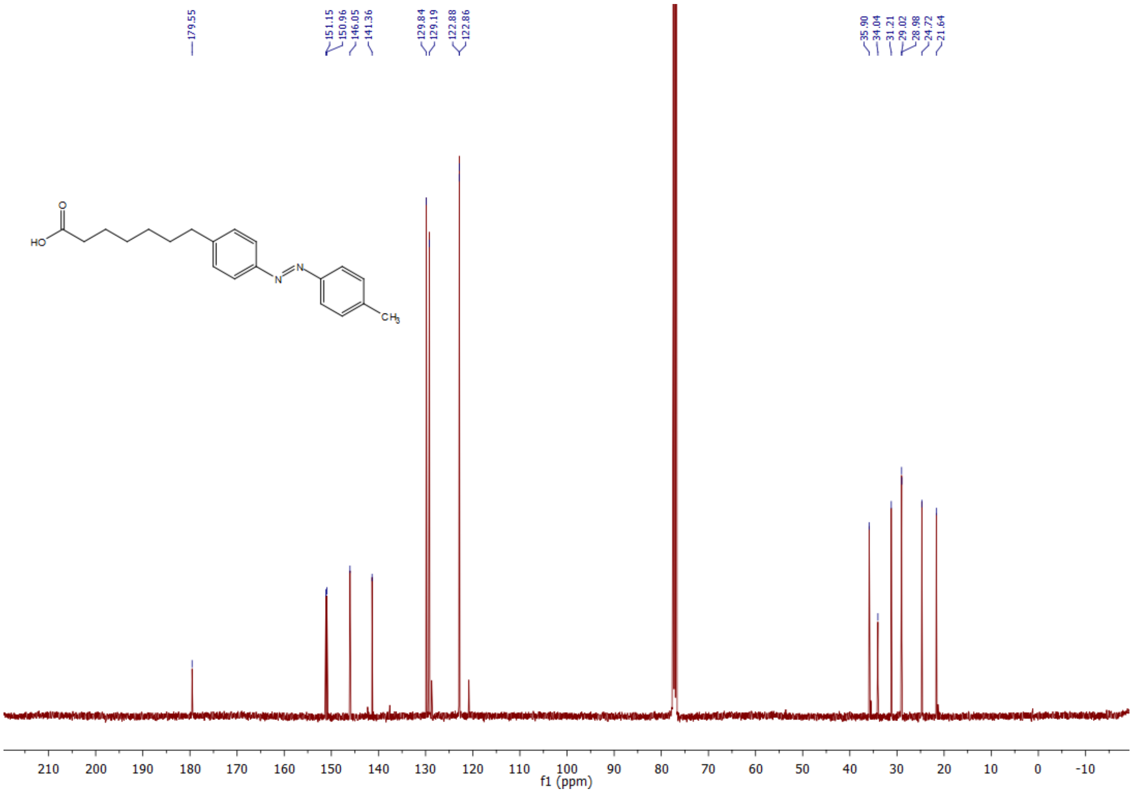

Supplementary Figure 38 | ^{1}H- and ^{13}C-NMR spectra of 7-(4-(*p*-tolyldiazenyl)phenyl)heptanoic acid (**FAAzo7**).

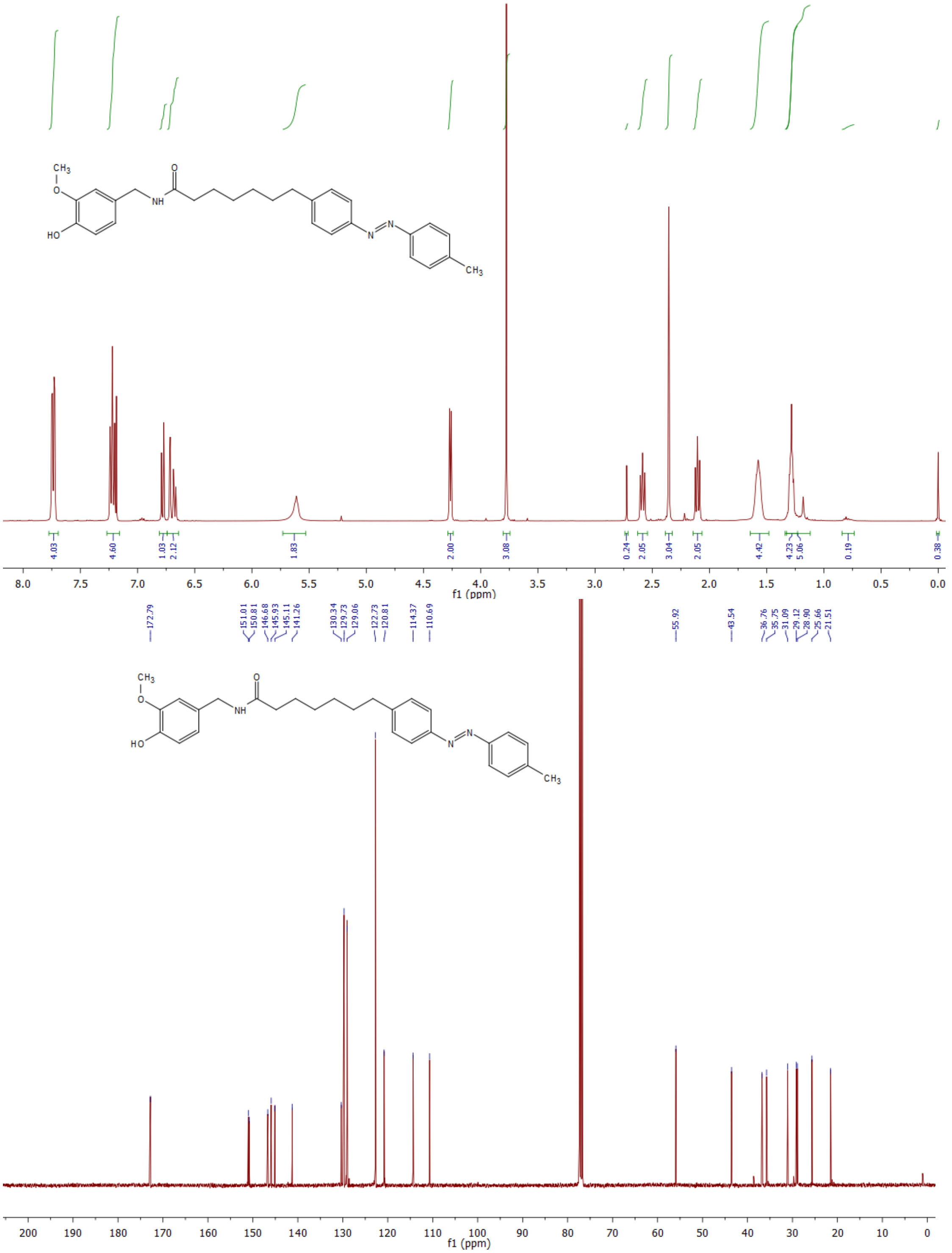

Supplementary Figure 39 | ^{1}H- and ^{13}C-NMR spectra of *N*-(4-hydroxy-3-methoxybenzyl)-7-(4-(*p*-tolyldiazenyl)phenyl)heptanamide (**AzCA7**).

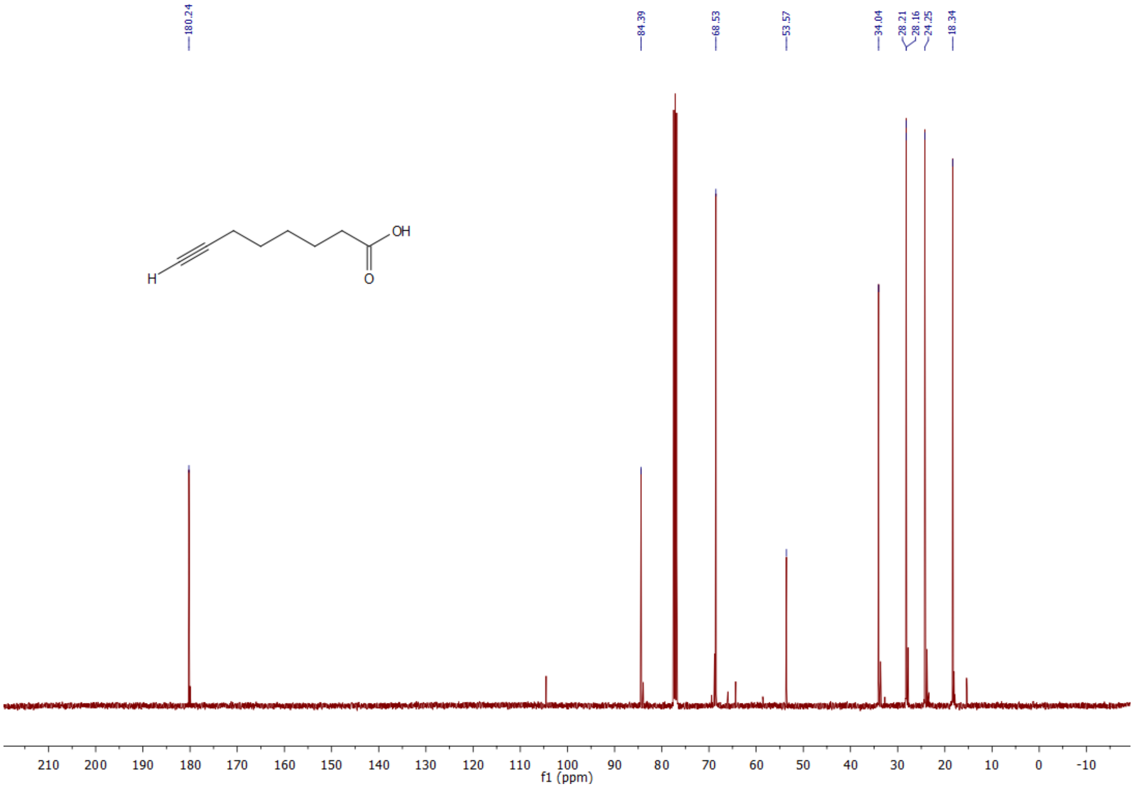

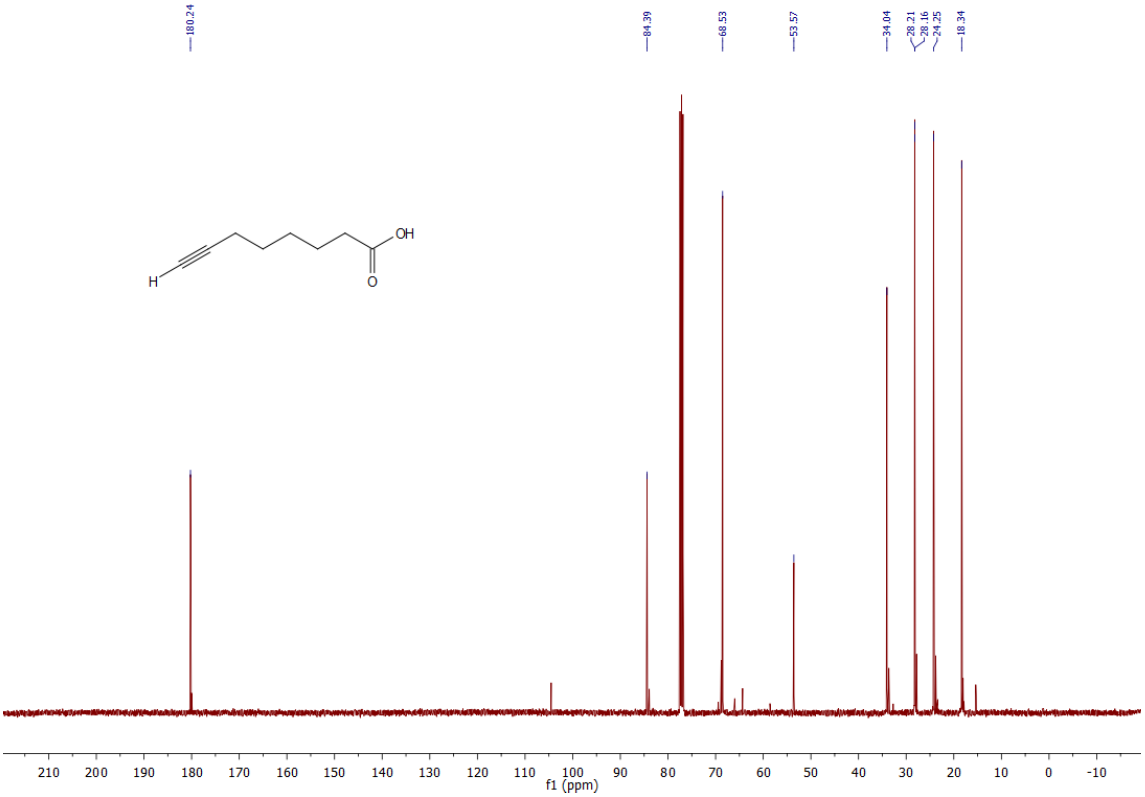

Supplementary Figure 40 | ^{1}H- and ^{13}C-NMR spectra of oct-7-ynoic acid (**1d**).

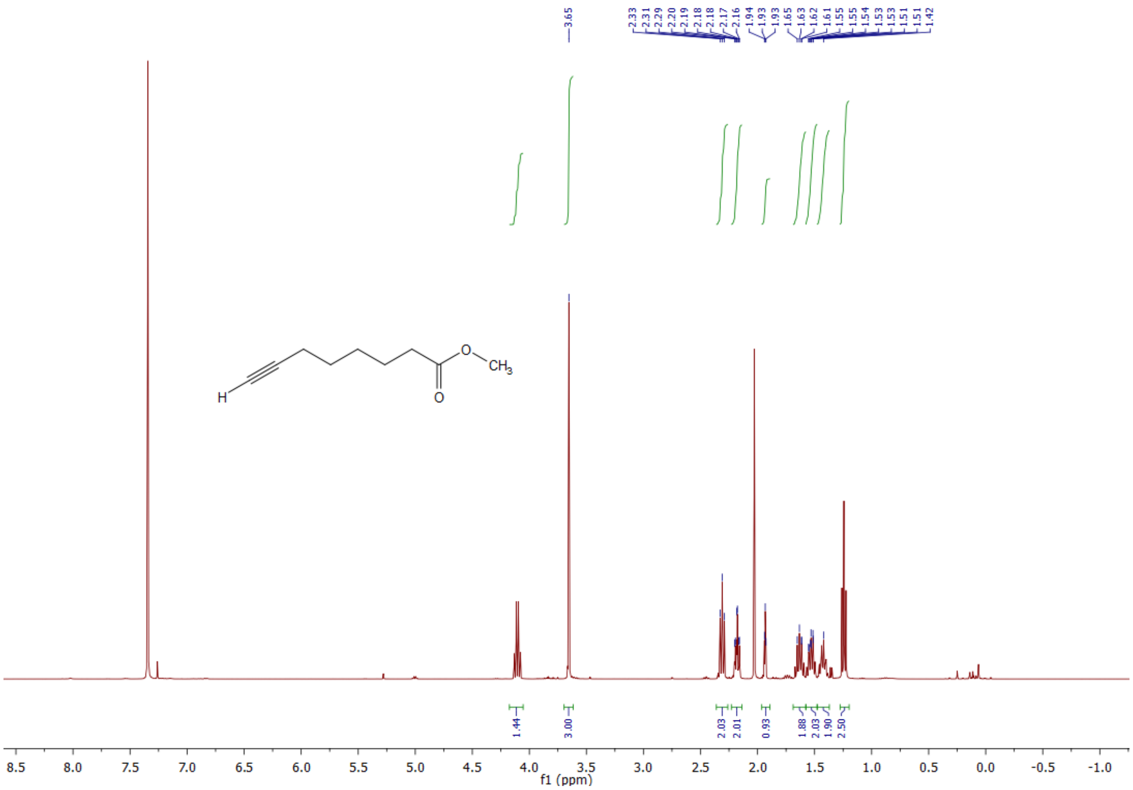

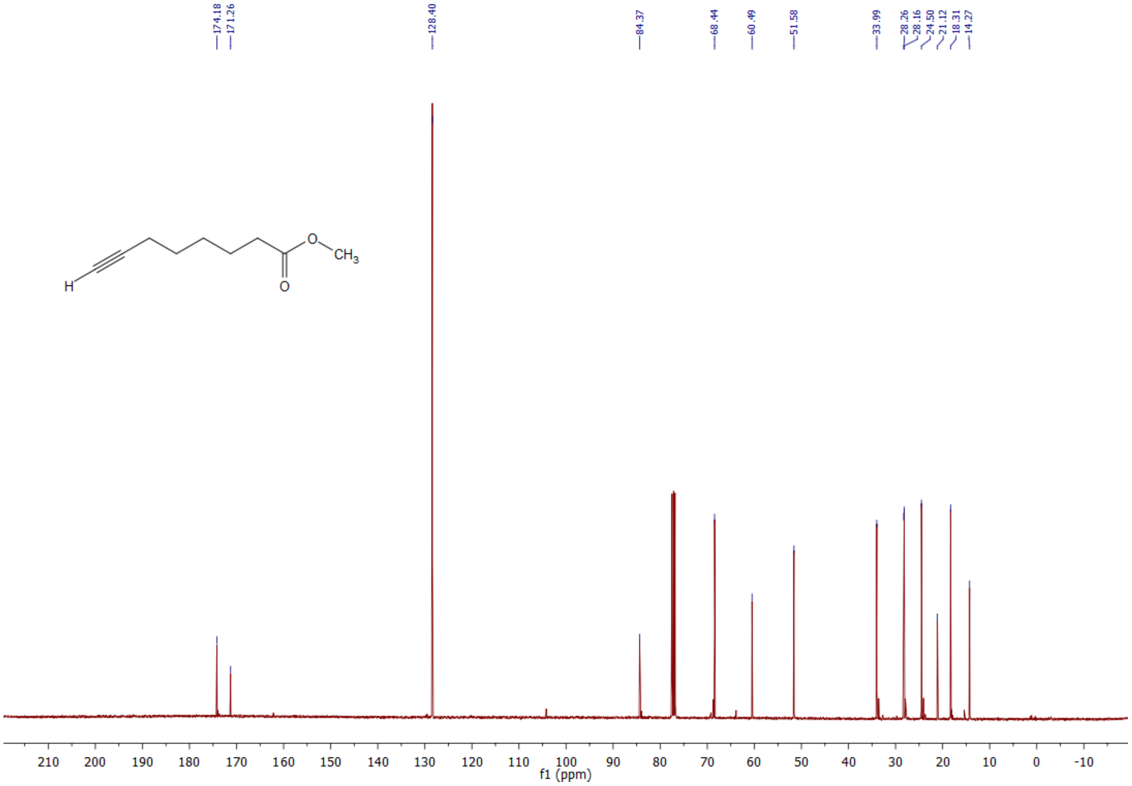

Supplementary Figure 41 | ^{1}H- and ^{13}C-NMR spectra of methyl oct-7-ynoate (**2d**).

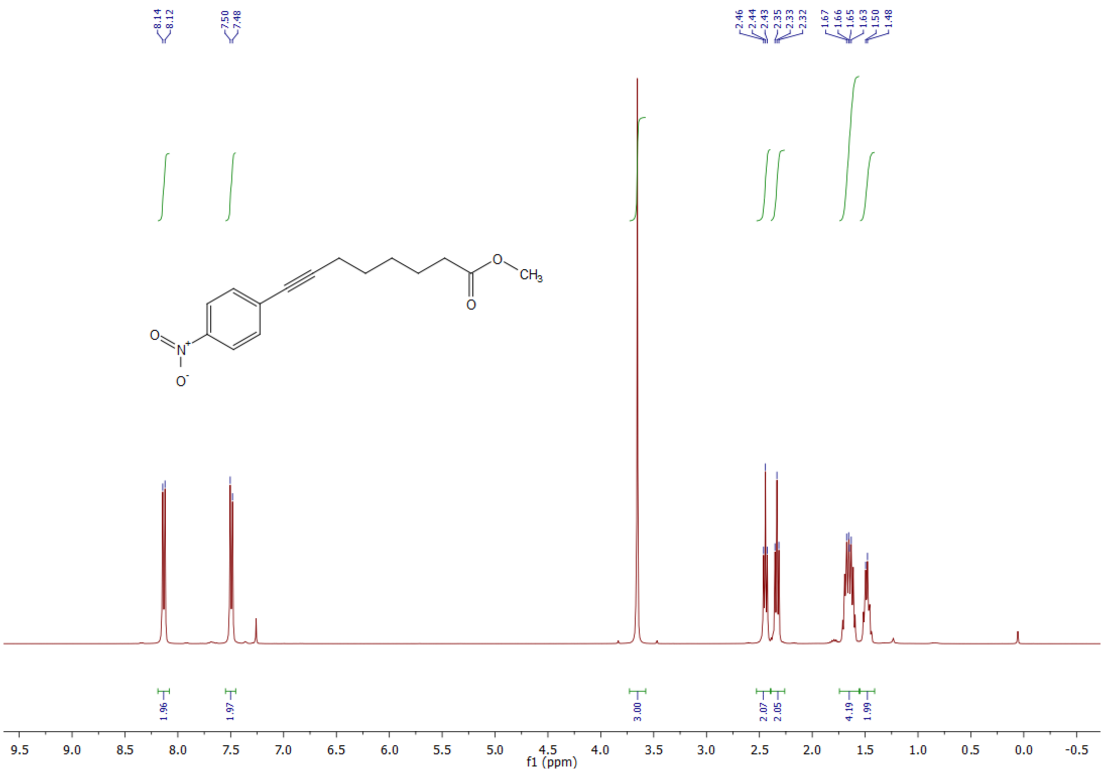

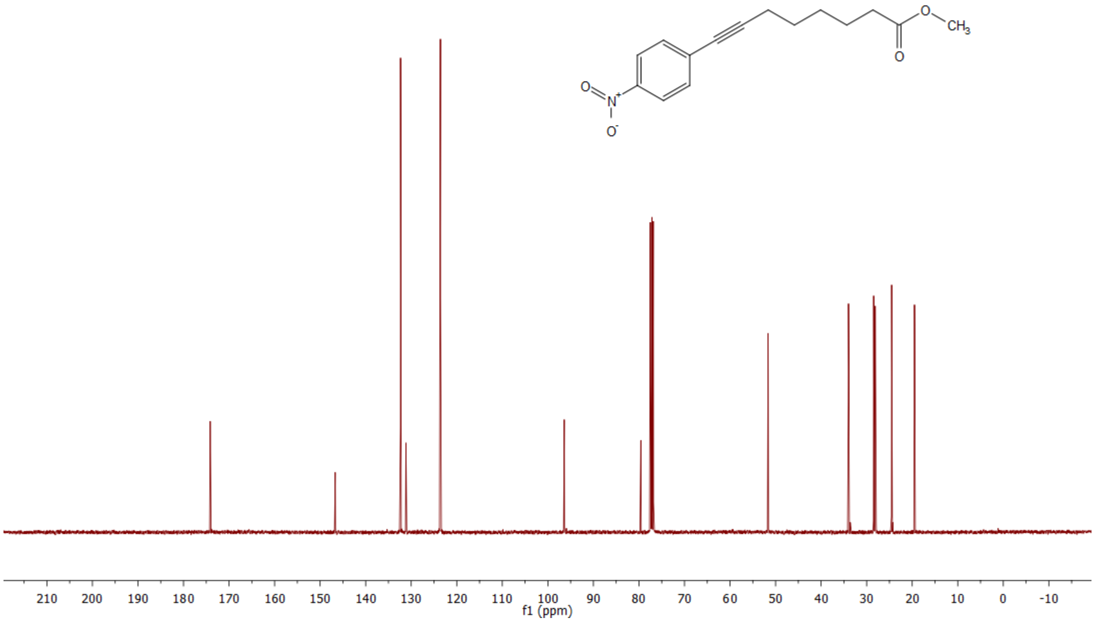

Supplementary Figure 42 | ^{1}H- and ^{13}C-NMR spectra of methyl 8-(4-nitrophenyl)oct-7-ynoate (**3d**).

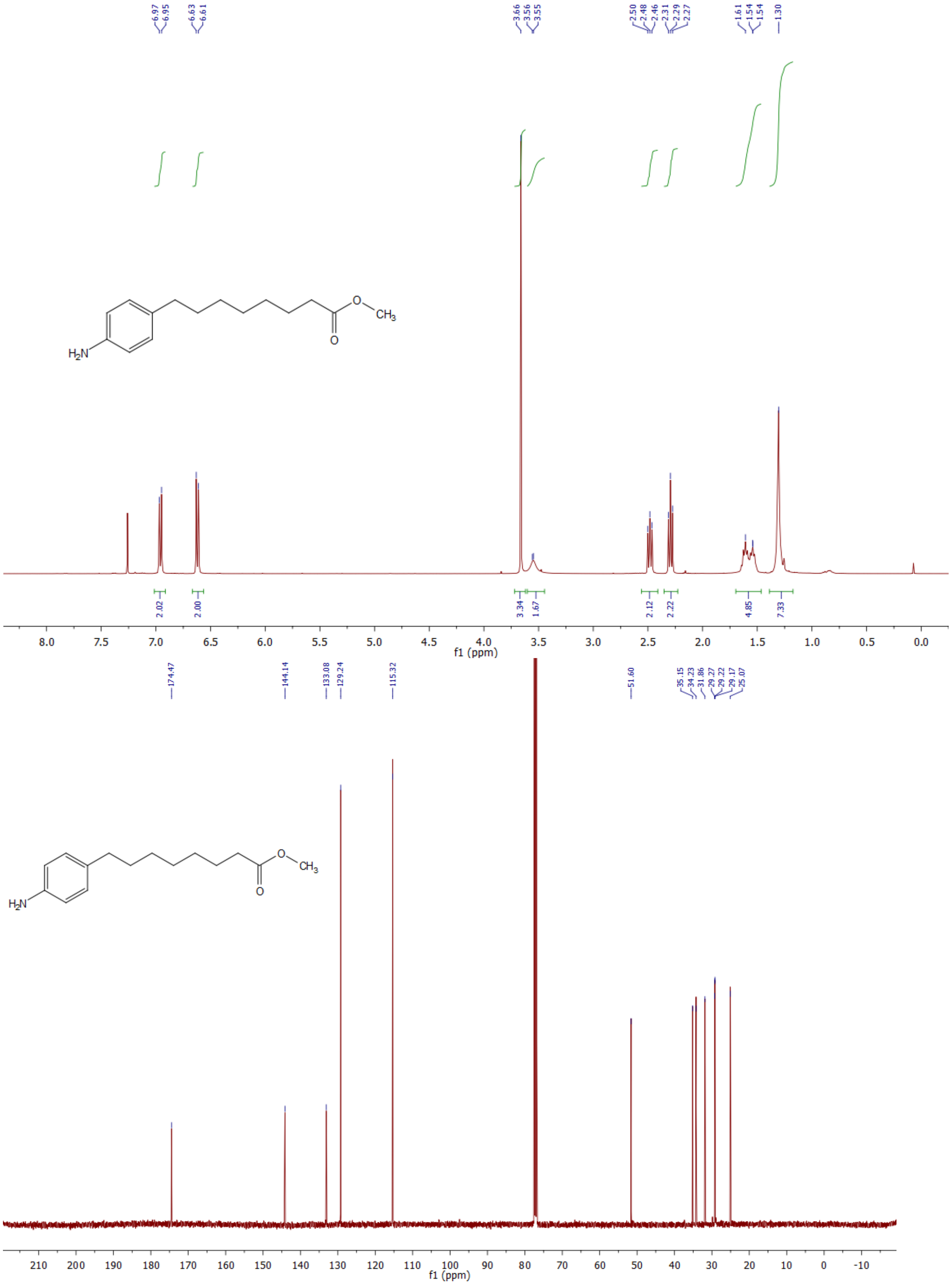

Supplementary Figure 43 | ^{1}H- and ^{13}C-NMR spectra of methyl 8-(4-aminophenyl)octanoate (**4d**).

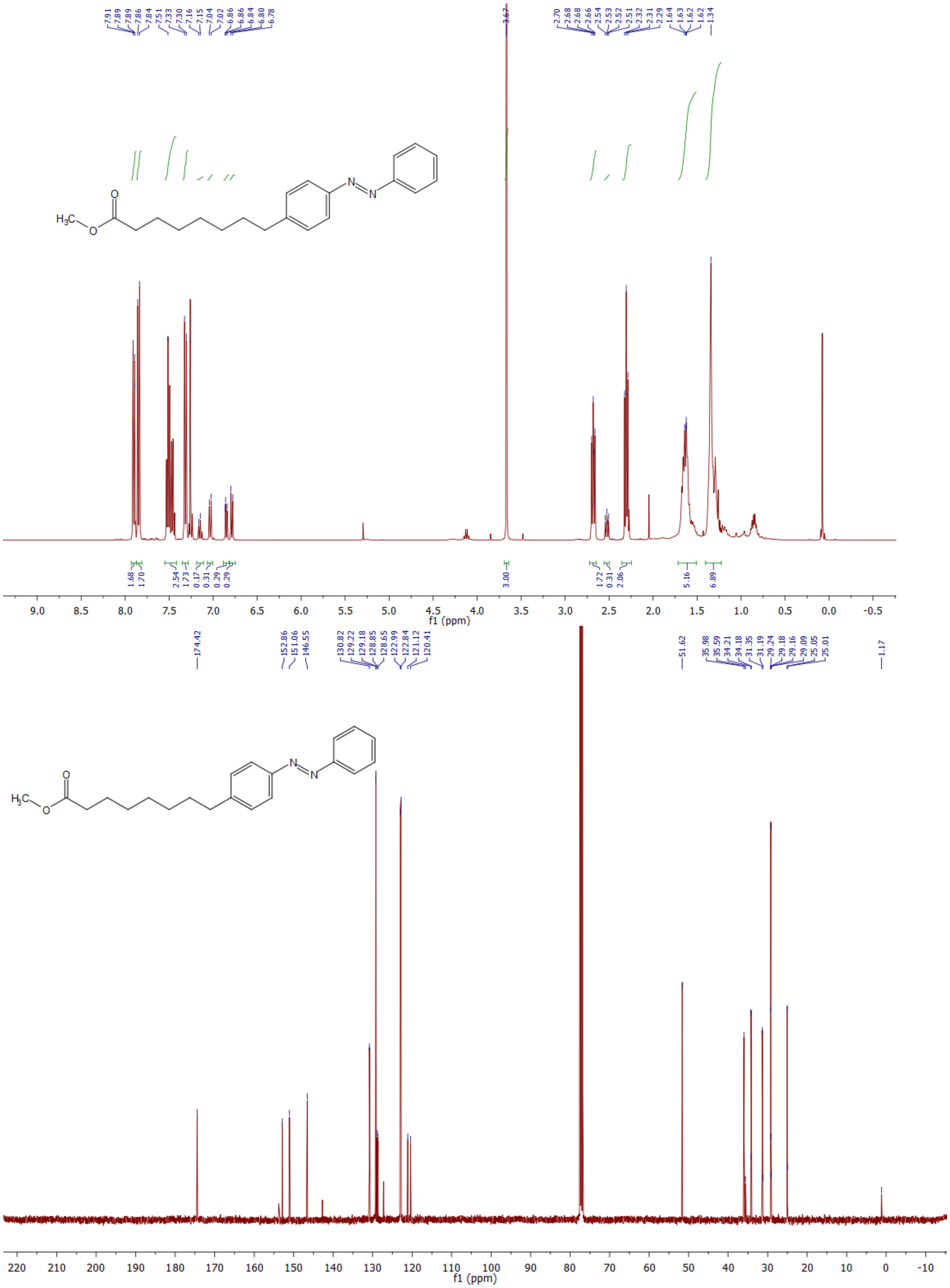

Supplementary Figure 44 | ^{1}H- and ^{13}C-NMR spectra of methyl 8-(4-(phenyldiazenyl)phenyl)octanoate (**5d**).

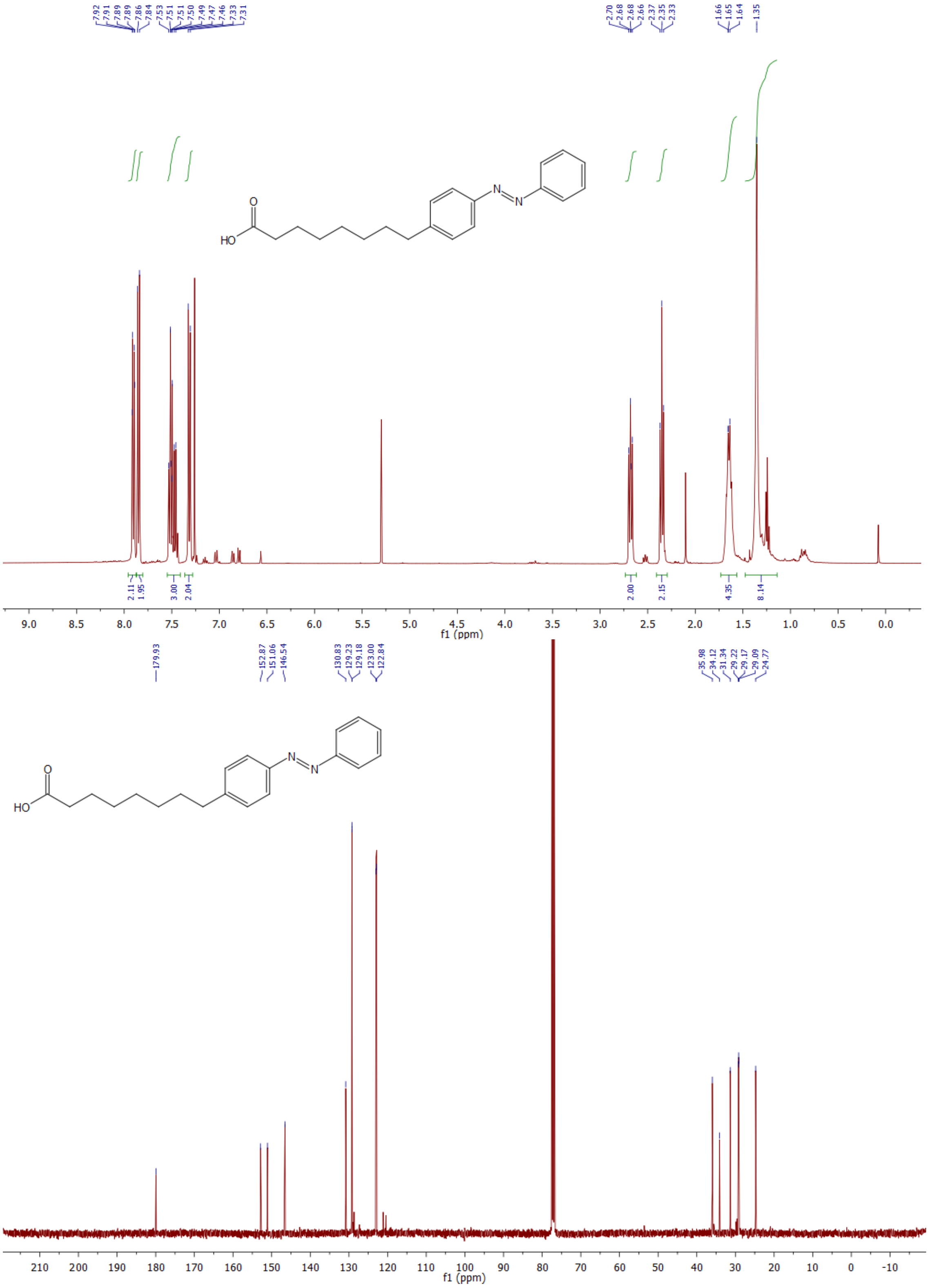

Supplementary Figure 45 | ^{1}H- and ^{13}C-NMR spectra of 8-(4-(phenyldiazenyl)phenyl)octanoic acid (**FAAzo8**).

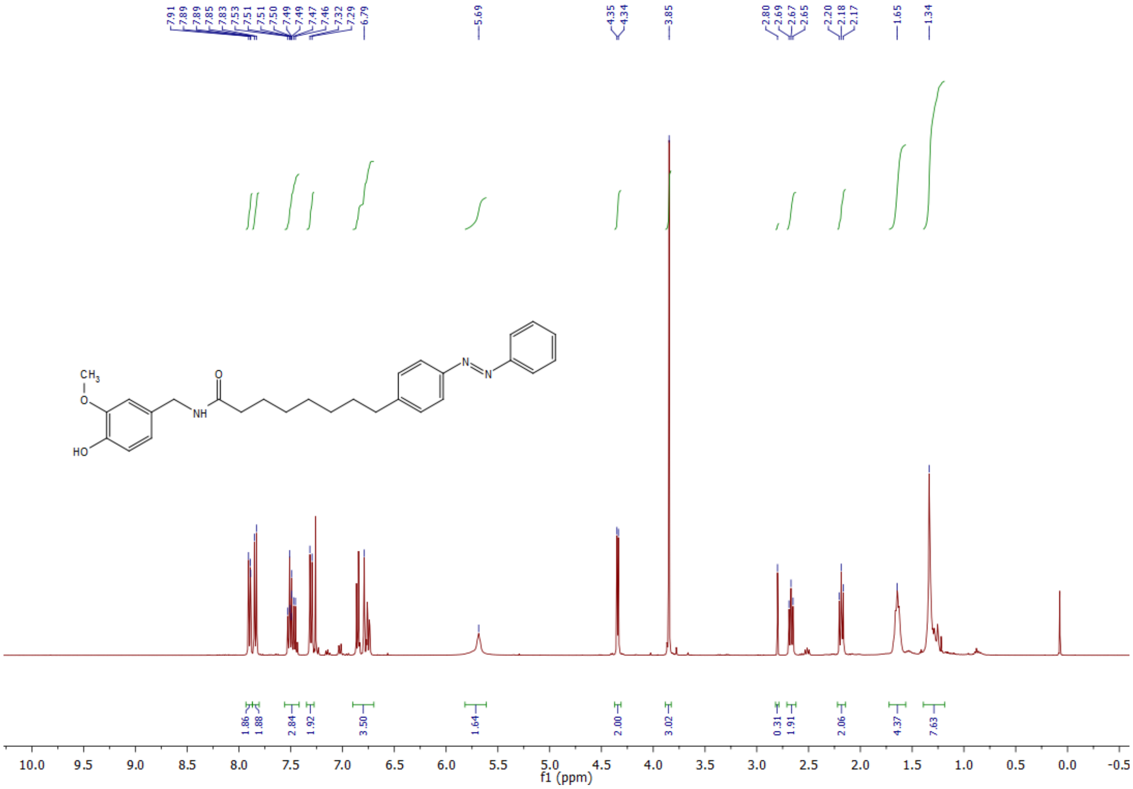

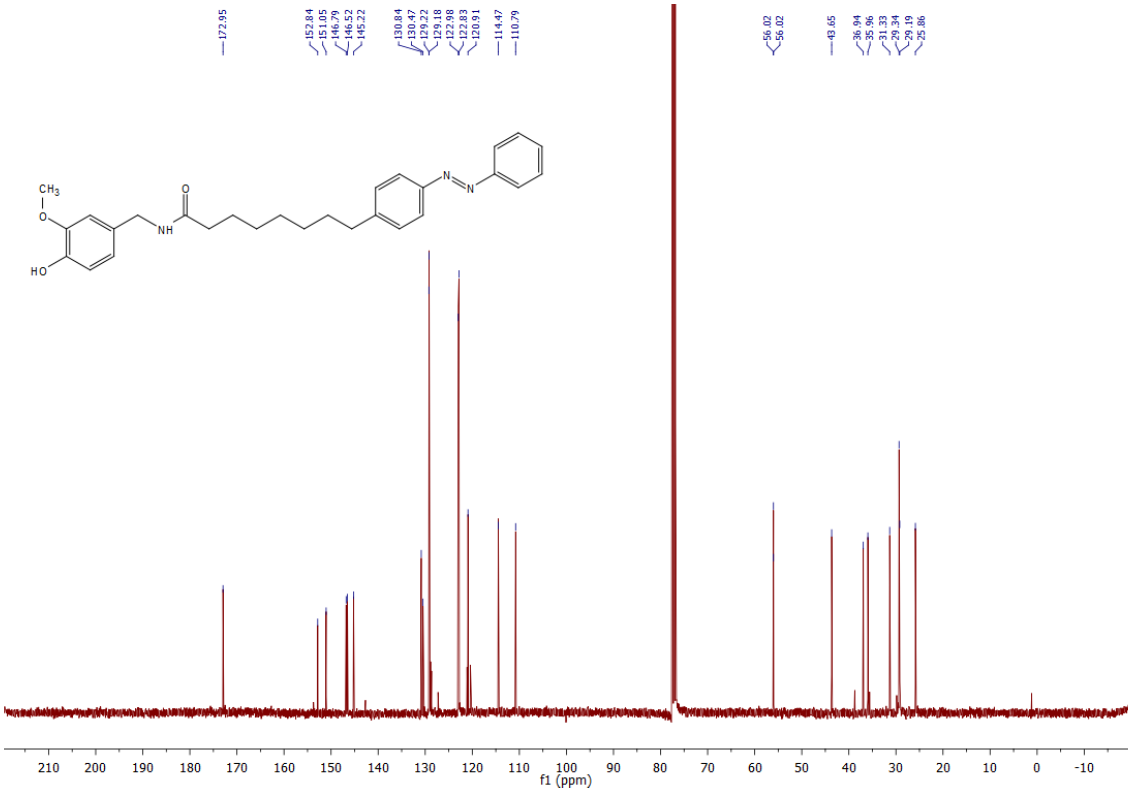

Supplementary Figure 46 | ^{1}H- and ^{13}C-NMR spectra of *N*-(4-hydroxy-3-methoxybenzyl)-8-(4-(phenyldiazenyl)phenyl)octanamide (**AzCA8**).

Supplementary Methods

Preparation of *N*-(4-hydroxy-3-methoxybenzyl)-4-((4-heptylphenyl)diazenyl)benzamide (AzCA1)

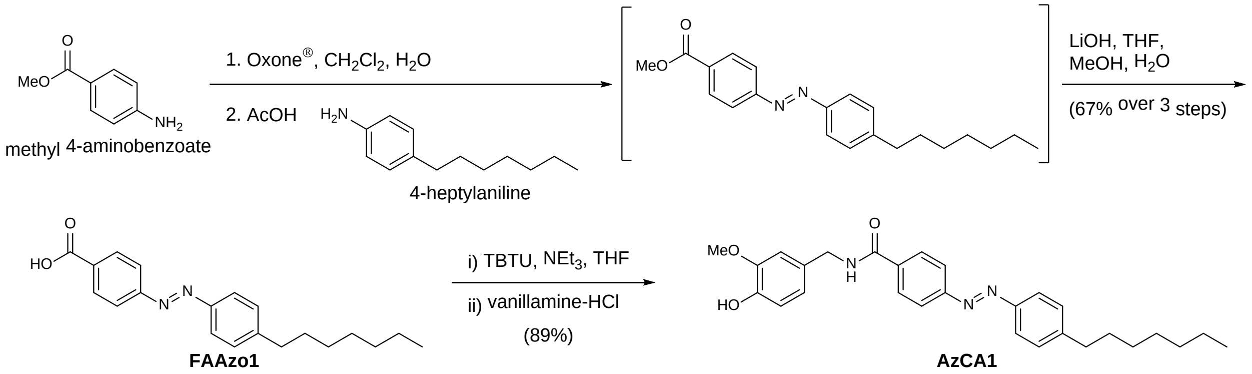

4-((4-Heptylphenyl)diazenyl)benzoic acid (FAAzo1)

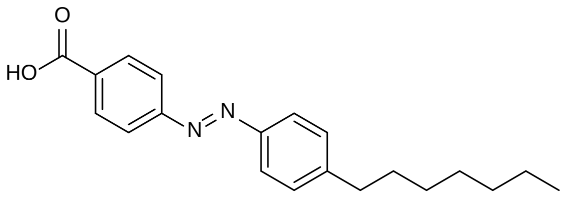

FAAzo1

A solution of methyl 4-aminobenzoate (0.30 g, 2.0 mmol, 1.0 equiv.) in CH_2Cl_2 (20 mL) was treated with a solution of Oxone® (1.52 g, 5 mmol, 2.5 equiv.) in H_2O (20 mL) at 23 °C. The resulting biphasic reaction mixture was stirred vigorously at room temperature overnight or until all the aniline was consumed, as observed by TLC analysis. Subsequently, the phases were separated and the aqueous phase was extracted with CH_2Cl_2 (2x20 mL). The combined organic extracts were washed with H_2O (2x30 mL) and to the washed organic solution was then added 4-heptylaniline (0.38 g, 2.0 mmol, 1.0 equiv.) followed by AcOH (20 mL). The CH_2Cl_2 was removed under reduced pressure and the solution was left to stir overnight. The reaction mixture was then concentrated under reduced pressure and the residue was dissolved in CH_2Cl_2 (200 mL). The organic phase was washed with a saturated aqueous sodium bicarbonate solution (2x50 mL), followed by H_2O (3x75 mL). The washed organic phase was dried over anhydrous sodium sulfate and then filtered. The filtrate was then concentration under reduced pressure. The residue was dissolved in THF (18 mL) and MeOH (18 mL). This solution was

treated with a 1 M aqueous lithium hydroxide solution (9 mL) and this solution was stirred for 2 h. The organic solvent was then removed under reduced pressure. The mixture was then acidified with a 2 M aqueous hydrochloric acid solution, and extracted with EtOAc (2x30 mL). The combined organic phases were washed with H_2O (2x40 mL), the washed solution was dried over anhydrous sodium sulfate and the dried solution was filtered. The filtrate was then concentrated under reduced pressure, and after being pre-absorbed onto SiO_2 (5 g, loaded from THF), the residue was purified by flash silica gel chromatography (50 g SiO_2, 99:1 CH_2Cl_2:H_2O) to yield **4-((4-heptylphenyl)diazenyl)benzoic acid** (**FAAzo1**, 450 mg, 67%) as a red solid.

TLC (CH_2Cl_2): Rf = 0.35 (*cis*), 0.52 (*trans*). **^{1}H NMR (D_6-DMSO, 400 MHz, 25 °C):** δ 8.13 (d, 2H, $H3_{a,b}$, $J_{3,4}$ = 8.6 Hz), 7.94 (d, 2H, $H4_{a,b}$, $J_{4,3}$ = 8.6 Hz), 7.86 (d, 2H, $H9_{a,b}$, $J_{9,10}$ = 8.4 Hz), 7.43 (d, 2H, $H10_{a,b}$, $J_{10,9}$ = 8.4 Hz), 2.66 (m, 2H, $H12_{a,b}$), 1.64-1.54 (m, 2H, $H13_{a,b}$), 1.31-1.19 (m, 8H, $H14_{a,b}$, $H15_{a,b}$, $H16_{a,b}$, $H17_{a,b}$), 0.84 (t, 3H, $H18_{a,b,c}$, $J_{18,17}$ = 6.9 Hz). **^{13}C NMR (D_6-DMSO, 100 MHz, 25 °C):** δ 166.8 (C1), 154.4 (C5), 150.2 (C8), 147.4 (C11), 132.6 (C2), 130.6 ($C3_{a,b}$), 129.5 ($C10_{a,b}$), 123.0 ($C9_{a,b}$), 122.5 ($C4_{a,b}$), 35.1 (C12), 31.3 (C_{alk}), 30.8 (C13), 28.7 (C_{alk}), 28.6 (C_{alk}), 22.1 (C_{alk}), 14.0 (C18). **IR (neat, ATR): $\tilde{\nu}$** = 2952, 2920, 2850, 2662, 2543, 1678, 1601, 1582, 1501, 1424, 1289, 1221, 1143, 1125, 1111, 1098, 1011, 944, 868, 840, 805, 755, 722, 690, 672. **HRMS (EI^+):** *m/z* calcd. for $[C_{20}H_{24}N_2O_2]^+$: 324.1838, found: 324.1834 ($[M-e^-]^+$).

N-(4-Hydroxy-3-methoxybenzyl)-4-((4-heptylphenyl)diazenyl)benzamide (AzCA1)

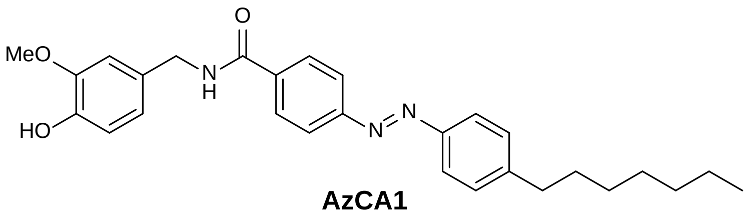

AzCA1

A solution of **4-((4-heptylphenyl)diazenyl)benzoic acid** (**FAAzo1**, 25 mg, 77 µmol, 1.0 equiv.) and TBTU (25 mg, 77 µmol, 1.0 equiv.) in EtOAc (3.0 mL) was treated with NEt_3 (24 mg, 3.0 equiv.) under an argon atmosphere at room temperature. After 1 h, vanillamine hydrochloride (29 mg, 0.15 mmol, 2.0 equiv.) was added and stirring was continued at room temperature for 2 h. The solution was then diluted with EtOAc (15 mL) and washed with H_2O (2x20 mL). The phases were separated and the organic layer was dried over anhydrous sodium sulfate. The solvent was removed under reduced pressure and the residue was purified by flash silica gel chromatography (9.0 g SiO_2, 40:1 CH_2Cl_2:MeOH) to yield ***N*-(4-hydroxy-3-methoxybenzyl)-4-((4-heptylphenyl)diazenyl)benzamide** (**AzCA1**, 31 mg, 89%) as a red solid.

TLC (hexanes/EtOAc, 1:1): Rf = 0.48. **^{1}H NMR ($CDCl_3$, 400 MHz, 25 °C):** δ 7.95-7.90 (m, 4H, $H3_{a,b}$, $H4_{a,b}$), 7.88-7.84 (m, 2H, $H9_{a,b}$, $J_{9,10}$ = 8.4 Hz), 7.35-7.31 (m, 2H, $H10_{a,b}$, $J_{10,9}$ = 8.4 Hz), 6.92-6.86 (m, 3H, H21, H24, H25), 6.40 (t, 1H, NH, $J_{NH,19}$ = 6.5 Hz), 5.63 (s, 1H, OH), 4.59 (d, 2H, $H19_{a,b}$, $J_{19,NH}$ = 6.5 Hz), 3.90 (s, 3H, $H26_{a,b,c}$), 2.69 (t, 2H, $H12_{a,b}$, $J_{12,13}$ = 7.6 Hz), 1.70-1.61 (m, 2H, $H13_{a,b}$), 1.37-1.23 (m, 8H, $H14_{a,b}$, $H15_{a,b}$, $H16_{a,b}$, $H17_{a,b}$), 0.88 (t, 3H, $H18_{a,b,c}$, $J_{18,17}$ = 6.5 Hz).**^{13}C NMR ($CDCl_3$, 100 MHz, 25 °C):** δ 166.7 (C1), 154.5 (C_{azo}), 150.9 (C_{azo}), 147.5 (C2), 146.9 (C22), 145.4 (C23), 135.9 (C11), 130.0 (C20), 129.3 (2C, C_{azo}), 128.1 (2C, C_{azo}), 123.3 (2C, C_{azo}), 122.9 (2C, C_{azo}), 121.2 (C21), 114.6 (C24), 111.0 (C25), 56.1 (C26), 44.4 (C19), 36.1 (C12), 31.9 (C_{alk}), 31.4 (C13), 29.4 (C_{alk}), 29.3 (C_{alk}), 22.8 (C17), 14.2 (C18). **IR (neat, ATR):** $\tilde{\nu}$ = 3350, 3199, 2922, 2851, 1627, 1605, 1572, 1547, 1523, 1491, 1452, 1360, 1287, 1214, 1159, 1127, 1040, 868, 809, 773. **HRMS (ESI$^+$):** *m/z* calcd. for $[C_{28}H_{34}N_3O_3]^+$: 460.2600, found: 460.2592 ($[M+H]^+$).

Preparation of *N*-(4-hydroxy-3-methoxybenzyl)-2-(4-((4-hexylphenyl)diazenyl)phenyl) acetamide (AzCA2)

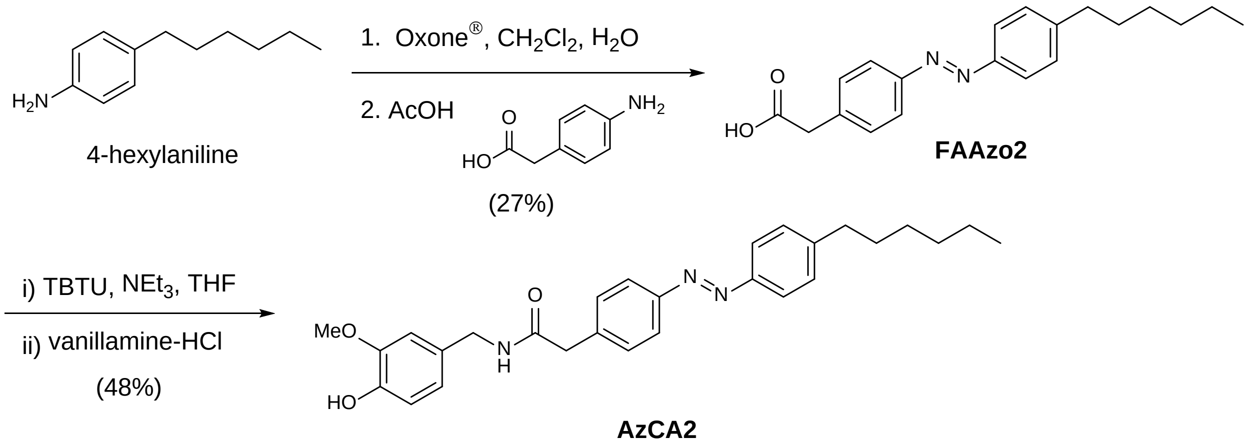

2-(4-((4-Hexylphenyl)diazenyl)phenyl)acetic acid (FAAzo2)

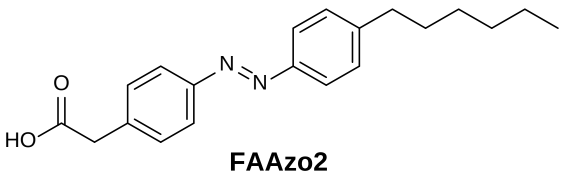

FAAzo2

A solution of 4-hexylaniline (3.0 g, 17 mmol, 1.5 equiv.) in CH_2Cl_2 (200 mL), was treated with a solution of Oxone® (30.4 g, 99 mmol, 9.0 equiv.) in H_2O (200 mL) at 23 °C. The resulting biphasic reaction mixture was stirred vigorously at room temperature overnight or until all the aniline was consumed, as observed by TLC analysis. Subsequently, the phases were separated and the aqueous phase was extracted with CH_2Cl_2 (2x140 mL). The combined organic extracts were washed sequentially with a 1 M aqueous hydrochloric acid solution (240 mL), saturated aqueous sodium bicarbonate solution (240 mL), and H_2O (3x240 mL). The washed organic layer was treated with 4-aminophenylacetic acid (1.7 g, 11 mmol, 1.0 equiv.) and AcOH (150 mL). The CH_2Cl_2 was then removed under reduced pressure at 35 °C and the solution was stirred overnight. The AcOH was then removed under reduced pressure and the red oil was azeotroped from toluene (100 mL). The residue was then purified by flash silica gel chromatography (0.30 kg SiO_2, 99:1 CH_2Cl_2:AcOH) to yield **2-(4-((4-hexylphenyl)diazenyl) phenyl)acetic acid** (**FAAzo2**, 1.00 g, 27%) as a red solid.

TLC (hexanes/EtOAc, 1:1): Rf = 0.79. **^{1}H NMR (D_6-DMSO, 400 MHz, 25 °C):** δ 12.45 (s, 1H, H_{COOH}), 7.84-7.79 (m, 4H, $H5_{a,b}$, $H10_{a,b}$, $J_{5,4} \approx J_{10,11} \approx 8.3$ Hz), 7.49-7.46 (m, 2H, $H11_{a,b}$, $J_{11,10} \approx 8.4$ Hz), 7.42-7.38 (m, 2H, $H4_{a,b}$, $J_{4,5} \approx 8.4$ Hz), 3.69 (s, 2H, $H2_{a,b}$), 2.66 (t, 2H, $H13_{a,b}$, $J_{13,14} = 7.4$ Hz), 1.65-1.56 (m, 2H, $H14_{a,b}$), 1.35-1.22 (m, 6H, $H15_{a,b}$, $H16_{a,b}$, $H17_{a,b}$), 0.85 (t, 3H, $H18_{a,b,c}$, $J_{18,17} = 7.0$ Hz). **^{13}C-NMR (D_6-DMSO, 100 MHz, 25 °C):** δ 172.8 (C1), 151.1 (C6), 150.7 (C9), 146.9 (C12), 139.0 (C3), 131.0 ($C4_{a,b}$), 129.8 ($C11_{a,b}$), 123.0 ($C10_{a,b}$), 122.8 ($C5_{a,b}$), 40.9 (C2), 35.5 (C13), 31.5 (C_{alk}), 31.2 (C4), 28.8 (C_{alk}), 22.5 (C_{alk}), 14.4 (C18). **IR (neat, ATR):** $\tilde{\nu}$ = 3022, 2952, 2926, 2850, 2728, 2636, 1691, 1601, 1497, 1465, 1413, 1377, 1301, 1249, 1197, 1171, 1153, 1114, 1010, 904, 866, 844, 825, 783, 726, 677. **HRMS (EI$^+$):** *m/z* calcd. for [$C_{20}H_{24}N_2O_2$]: 324.1838, found: 324.1832 ([M-e$^-$]$^+$).

***N*-(4-Hydroxy-3-methoxybenzyl)-2-(4-((4-hexylphenyl)diazenyl)phenyl)acetamide (AzCA2)**

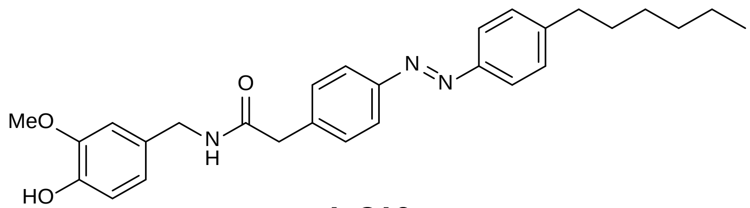

AzCA2

***N*-(4-((4-Hexylphenyl)diazenyl)phenyl)acetic acid** (**FAAzo2**, 25 mg, 77 µmol, 1.0 equiv.) was converted to red solid ***N*-(4-hydroxy-3-methoxybenzyl)-2-(4-((4-hexylphenyl)diazenyl) phenyl)acetamide** (**AzCA2**, 17 mg, 48%) in an analogous manner as described above for the preparation of ***N*-(4-hydroxy-3-methoxybenzyl)-4-((4-heptylphenyl)diazenyl)benzamide (AzCA1)**. *Note: all reagents and solvents were scaled according to molarity.*

TLC (hexanes/EtOAc, 1:2): Rf = 0.40. **^{1}H NMR ($CDCl_3$, 400 MHz, 25 °C):** δ 7.88-7.81 (m, 2H, $H5_{a,b}$, $H10_{a,b}$, $J_{5,4} \approx J_{10,11} \approx 8.3$ Hz), 7.41 (d, 2H, $H4_{a,b}$, $J_{4,5} = 8.3$ Hz), 7.32 (d, 2H, $H11_{a,b}$, $J_{11,10} = 8.3$ Hz), 6.84-6.80 (m, 1H, H24), 6.71-6.64 (m, 2H, H25, H21), 5.75 (t, 1H, NH), 5.66 (s, 1H, OH), 4.34 (d, 2H, $H19_{a,b}$, $J_{19,NH} = 5.7$ Hz), 3.80 (s, 3H, H_{OMe}), 3.67 (s, 2H, $H2_{a,b}$), 2.68 (t, 2H, $H13_{a,b}$, $J_{13,14} = 7.7$ Hz), 1.72-1.61 (m, 2H, $H14_{a,b}$), 1.40-1.23 (m, 6H, $H15_{a,b}$, $H16_{a,b}$, $H17_{a,b}$), 0.89 (t, 3H, $H18_{a,b,c}$, $J_{18.17} = 6.7$ Hz). **^{13}C NMR ($CDCl_3$, 100 MHz, 25 °C):** δ 170.3 (C1), 152.1 (C_{azo}), 151.0 (C_{azo}), 147.0 (C3), 146.8 (C22), 145.2 (C23), 137.6 (C12), 130.2 (2C, C_{azo}), 130.0 (C20), 129.3 (2C, C_{azo}), 123.4 (2C, C_{azo}), 123.0 (2C, C_{azo}), 120.7 (2C, C_{azo}), 114.5 (C24), 110.4 (C25), 56.0 (C26), 43.8 (C2), 43.8 (C19), 36.0 (C13), 31.8 (C15), 31.4 (C14), 29.1 (C16), 22.7 (C17), 14.2 (C18). **IR (neat, ATR): $\tilde{\nu}$** = 3457, 3285, 2953, 2922, 2852, 1648, 1635, 1601, 1518, 1462, 1434, 1428, 1339, 1276, 1248, 1234, 1194, 1152, 1120, 1039, 1019, 1011, 838, 797, 716, 677. **HRMS (ESI$^+$):** *m/z* calcd. for $[C_{28}H_{34}N_3O_3]^+$: 460.2600, found: 460.2592 ($[M+H]^+$).

Preparation of *N*-(4-hydroxy-3-methoxybenzyl)-3-(4-((4-pentylphenyl)diazenyl)phenyl) propanamide (AzCA3)

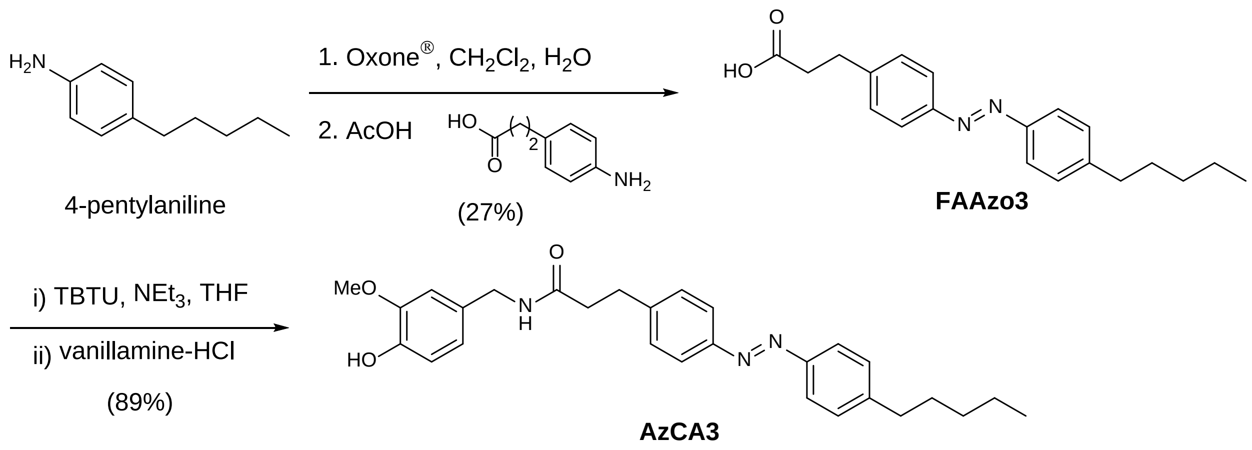

3-(4-((4-Pentylphenyl)diazenyl)phenyl)propanoic acid (FAAzo3)

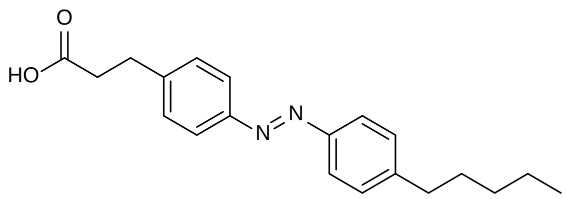

FAAzo3

4-Pentylaniline (1.5 g, 9.2 mmol, 1.5 equiv.) and 4-aminophenylpropionic acid (1.0 g, 6.1 mmol, 1.0 equiv.) were converted to red solid **3-(4-((4-pentylphenyl)diazenyl)phenyl)propanoic acid** (**FAAzo3**, 1.0 g, 27%) in an analogous manner as described above for the preparation of **2-(4-((4-hexylphenyl)diazenyl)phenyl)acetic acid (FAAzo2).** *Note: all reagents and solvents were scaled according to molarity.*

TLC (hexanes/EtOAc, 10:1): Rf = 0.74. **^{1}H NMR ($CDCl_3$, 400 MHz, 25 °C):** δ 7.87-7.81 (m, 4H, $H6_{a,b}$, $H11_{a,b}$), 7.37-7.30 (m, 4H, $H5_{a,b}$, $H12_{a,b}$, $J_{12,11} \approx J_{5,6} \approx$ 8.4 Hz), 3.05 (t, 2H, $H2_{a,b}$, $J_{2,3}$ = 7.6 Hz), 2.75 (t, 2H, $H3_{a,b}$, $J_{3,2}$ = 7.7 Hz), 2.68 (t, 2H, $H14_{a,b}$), 1.71-1.62 (m, 2H, $H15_{a,b}$), 1.40-1.32 (m, 4H, $H16_{a,b}$, $H17_{a,b}$), 0.91 (t, 3H, $H18_{a,b,c}$, $J_{18,17}$ = 6.9 Hz). **^{13}C NMR ($CDCl_3$, 100 MHz, 25 °C):** δ 178.8 (C1), 151.6 (C_{azo}), 151.1 (C_{azo}), 146.6 (C_{azo}), 143.2 (C_{azo}), 129.2 (2C, C_{azo}), 129.1 (2C, C_{azo}), 123.1 (2C, C_{azo}), 122.9 (2C, C_{azo}), 36.0 (C14), 35.5 (C2), 31.6 (C3), 31.1 (C16), 30.6 (C15), 22.7 (C17), 14.2 (C18). **IR (neat, ATR):** $\tilde{\nu}$ = 3026, 2950, 2924, 2853, 2616, 1691, 1599, 1579, 1494, 1431, 1417, 1358, 1315, 1286, 1217, 1186, 1153, 1105, 1011, 932, 837, 806, 725, 678. **HRMS (EI$^+$):** *m/z* calcd. for [$C_{20}H_{24}N_2O_2$]: 324.1838, found: 324.1831 ($[M-e^-]^+$).

***N*-(4-Hydroxy-3-methoxybenzyl)-3-(4-((4-pentylphenyl)diazenyl)phenyl)propanamide (AzCA3)**

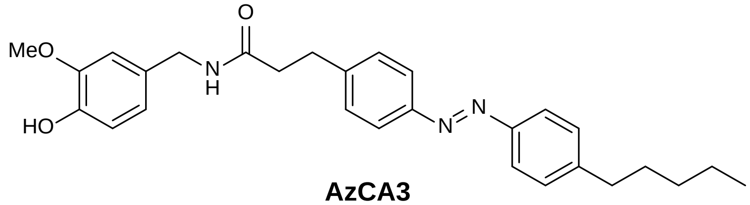

AzCA3

3-(4-((4-)Pentylphenyl)diazenyl)phenyl)propanoic acid (**FAAzo3**, 25 mg, 77 µmol, 1.0 equiv.) was converted to red solid ***N*-(4-hydroxy-3-methoxybenzyl)-3-(4-((4-pentylphenyl)diazenyl) phenyl)propanamide** (**AzCA3**, 17 mg, 89%) in an analogous manner as described above for the preparation of ***N*-(4-hydroxy-3-methoxybenzyl)-4-((4-heptylphenyl)diazenyl)benzamide (AzCA1)**. *Note: all reagents and solvents were scaled according to molarity.*

TLC (CH_2Cl_2:MeOH:AcOH, 95:5:1): Rf = 0.47. **^{1}H NMR ($CDCl_3$, 400 MHz, 25 °C):** δ 7.85-7.78 (m, 4H, $H6_{a,b}$, $H11_{a,b}$, $J_{6,5} \approx J_{11,12} \approx$ 8.3 Hz), 7.35-7.30 (m, 4H, $H5_{a,b}$, $H12_{a,b}$, $J_{5,6} \approx J_{12,11} \approx$ 8.4 Hz), 6.82 (d, 1H, H22, $J_{22,21}$ = 7.8 Hz), 6.71-6.65 (m, 2H, H21, H25), 5.62-5.56 (m, 2H, NH, OH), 4.31 (d, 2H, $H19_{a,b}$, $J_{19,NH}$ = 5.8 Hz), 3.80 (s, 3H, $H26_{a,b,c}$), 3.07 (t, 2H, $H3_{a,b}$, $J_{3,2}$ = 7.5 Hz), 2.68 (t, 2H, $H14_{a,b}$, $J_{14,15}$ = 8.0 Hz), 2.53 (t, 2H, $H2_{a,b}$, $J_{2,3}$ = 7.4 Hz), 1.70-1.60 (m, 2H, $H15_{a,b}$), 1.39-1.31 (m, 4H, $H16_{a,b}$, $H17_{a,b}$), 0.90 (t, 3H, $H18_{a,b,c}$, $J_{18,17}$ = 6.8 Hz). **^{13}C NMR ($CDCl_3$, 100 MHz, 25 °C):** δ 171.6 (C1), 151.4 (C_{azo}), 151.0 (C_{azo}), 146.8 (C22), 146.6 (C_{azo}), 145.2 (C23), 144.0 (C_{azo}), 130.1 (C20), 129.2 (4C, C_{azo}), 123.0 (2C, C_{azo}), 122.9 (2C, C_{azo}), 121.0 (C21), 114.5 (C24), 110.7 (C25), 56.0 (C26), 43.8 (C19), 38.3 (C2), 36.0 (C14), 31.6 (C3), 31.6 (C16), 31.1 (C15), 22.7 (C17), 14.2 (C18). **IR (neat, ATR):** $\tilde{\nu}$ = 3285, 3052, 2953, 2927, 2855, 1642, 1600, 1513, 1462, 1451, 1429, 1375, 1272, 1235, 1155, 1123, 1034, 1012, 848, 735, 731. **HRMS (ESI$^+$):** *m/z* calcd. for $[C_{28}H_{34}N_3O_3]^+$: 460.2600, found: 460.2591 ($[M+H]^+$).

Preparation of *N*-(4-hydroxy-3-methoxybenzyl)-4-(4-((4-butylphenyl)diazenyl)phenyl) butanamide (AzCA4)

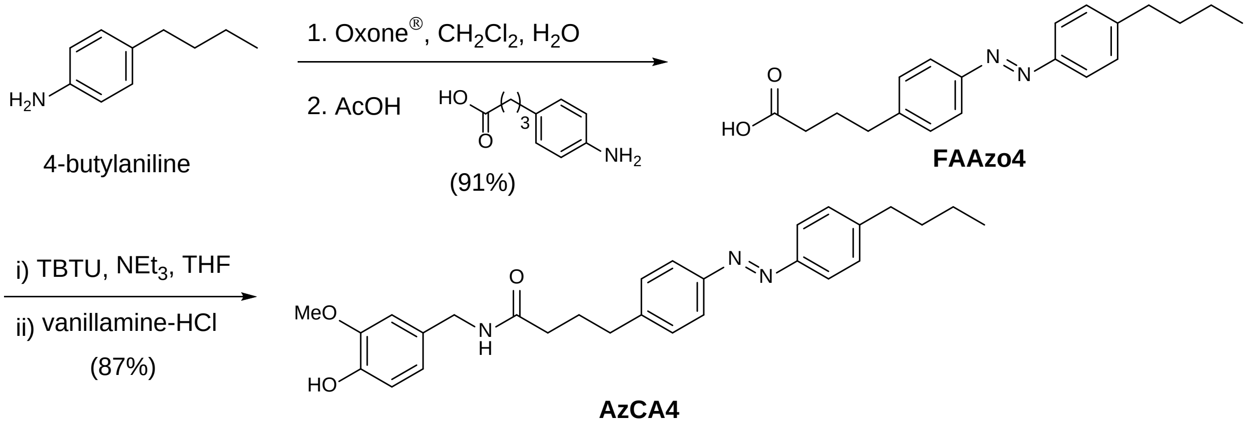

4-(4-((4-Butylphenyl)diazenyl)phenyl)butanoic acid (FAAzo4)

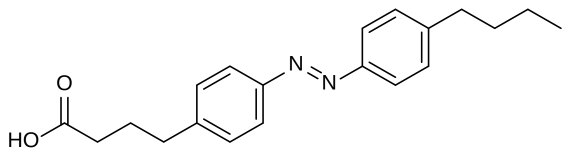

FAAzo4

4-Butylaniline (2.27 g, 15 mmol, 3.0 equiv.) and 4-(4-aminophenyl)butyric acid (0.90 g, 5.1 mmol, 1.0 equiv.) were converted to red solid **4-(4-((4-butylphenyl)diazenyl)phenyl)butanoic acid** (**FAAzo4**, 1.49 g, 91%) in an analogous manner as described above for the preparation of **2-(4-((4-hexylphenyl)diazenyl)phenyl)acetic acid (FAAzo2)**. *Note: all reagents and solvents were scaled according to molarity.*

TLC (CH_2Cl_2/AcOH, 99:1): Rf = 0.15. **^{1}H NMR ($CDCl_3$, 400 MHz, 25 °C):** δ 7.86-7.81 (m, 4H, $H7_{a,b}$, $H12_{a,b}$, $J_{7,6} \approx J_{12,13} \approx$ 8.4 Hz), 7.35-7.29 (m, 4H, $H6_{a,b}$, $H13_{a,b}$, $J_{6,7} \approx J_{13,12} \approx$ 8.4 Hz), 2.76 (t, 2H, $H4_{a,b}$, $J_{4,3}$ = 7.8 Hz), 2.71 (t, 2H, $H15_{a,b}$, $J_{15,16}$ = 7.8 Hz), 2.43 (t, 2H, $H2_{a,b}$, $J_{2,3}$ = 7.3 Hz), 2.08-1.98 (m, 2H, $H3_{a,b}$), 1.70-1.61 (m, 2H, $H16_{a,b}$), 1.44-1.34 (m, 2H, $H17_{a,b}$), 0.95 (t, 3H, $H18_{a,b,c}$, $J_{18,17}$ = 7.3 Hz). **^{13}C NMR ($CDCl_3$, 100 MHz, 25 °C):** δ 179.6 (C1), 151.3 (C8), 151.0 (C11), 146.4 (C14), 144.3 (C5), 129.2 (2C, C_{azo}), 129.1 (2C, C_{azo}), 122.9 (2C, C_{azo}), 122.8 (2C, C_{azo}), 35.6 (C15), 34.9 (C4), 33.5 (C16), 33.3 (C2), 26.0 (C3), 22.4 (C17), 14.0 (C18). **IR (neat, ATR):** $\tilde{\nu}$ = 3021, 2950, 2927, 2869, 2853, 2685, 2599, 1689, 1600, 1580, 1497, 1461, 1434, 1409, 1337, 1279, 1212, 1152, 1113, 1102, 1060, 1024, 1010, 901, 842, 824, 772, 730, 671. **HRMS (EI$^+$):** *m/z* calcd. for [$C_{20}H_{24}N_2O_2$]: 324.1838, found: 324.1836 ([M-e$^-$]$^+$).

***N*-(4-Hydroxy-3-methoxybenzyl)-4-(4-((4-butylphenyl)diazenyl)phenyl)butanamide (AzCA4)**

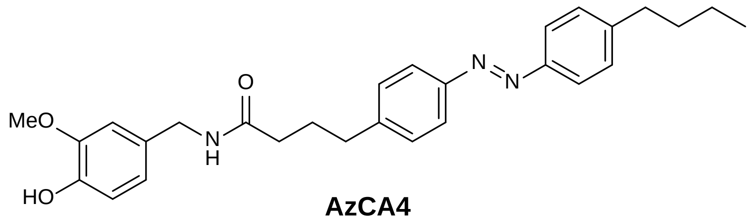

AzCA4

4-(4-((4-Butylphenyl)diazenyl)phenyl)butanoic acid (**FAAzo4**, 16 mg, 77 µmol, 1.0 equiv.) was converted to red solid ***N*-(4-hydroxy-3-methoxybenzyl)-4-(4-((4-butylphenyl)diazenyl)phenyl)butanamide** (**AzCA4**, 20 mg, 87%) in an analogous manner as described above for the preparation of ***N*-(4-hydroxy-3-methoxybenzyl)-4-((4-heptylphenyl)diazenyl)benzamide (AzCA1)**. *Note: all reagents and solvents were scaled according to molarity.*

TLC (hexanes/EtOAc, 1:1): Rf = 0.34. **^{1}H NMR ($CDCl_3$, 400 MHz, 25 °C):** δ 7.84-7.78 (m, 4H, $H12_{a,b}$, $H7_{a,b}$, $J_{12,13} \approx J_{7,6} \approx$ 8.4 Hz), 7.33-7.25 (m, 4H, $H6_{a,b}$, $H13_{a,b}$, $J_{6,7} \approx J_{13,12} \approx$ 8.4 Hz), 6.86 (d, 1H, H24, $J_{24,25}$ = 8.0 Hz), 6.80-6.73 (m, 2H, H21, H25), 5.77-5.68 (m, 2H, NH, OH), 4.34 (d, 2H, $H19_{a,b}$, $J_{19,NH}$ = 5.60 Hz), 3.85 (s, 3H, H26), 2.73 (t, 2H, $H4_{a,b}$, $J_{4,3}$ = 7.4 Hz), 2.68 (t, 2H, $H15_{a,b}$, $J_{15,16}$ = 7.6 Hz), 2.21 (t, 2H, $H2_{a,b}$, $J_{2,3}$ = 7.6 Hz), 2.08-1.99 (m, 2H, $H3_{a,b}$), 1.68-1.59 (m, 2H, $H16_{a,b}$), 1.44-1.30 (m, 2H, $H17_{a,b}$), 0.94 (t, 3H, $H18_{a,b,c}$, $J_{18,17}$ = 7.4 Hz). **^{13}C NMR ($CDCl_3$, 100 MHz, 25 °C):** δ 172.4 (C1), 151.4 (C8), 151.1 (C11), 146.8 (C22), 146.5 (C14), 145.3 (C23), 144.7 (C5), 130.3 (C20), 129.3 (2C, C_{azo}), 129.2 (2C, C_{azo}), 122.9 (2C, C_{azo}), 122.9 (2C, C_{azo}), 121.0 (C21), 114.5 (C24), 110.9 (C25), 56.1 (C26), 43.7 (C19), 35.9 (C2), 35.7 (C15), 35.1 (C4), 33.6 (C16), 27.0 (C3), 22.5 (C17), 14.1 (C18). **IR (neat, ATR):** $\tilde{\nu}$ = 3285, 3054, 2953, 2929, 2857, 1643, 1600, 1514, 1462, 1430, 1377, 1275, 1237, 1155, 1124, 1034, 1013, 845, 736, 677. **HRMS (EI^+):** *m/z* calcd. for [$C_{28}H_{33}N_3O_3$]: 459.2522, found: 459.2516 ($[M-e^-]^+$).

Preparation of *N*-(4-hydroxy-3-methoxybenzyl)-5-(4-((4-propylphenyl)diazenyl)phenyl) pentanamide (AzCA5)

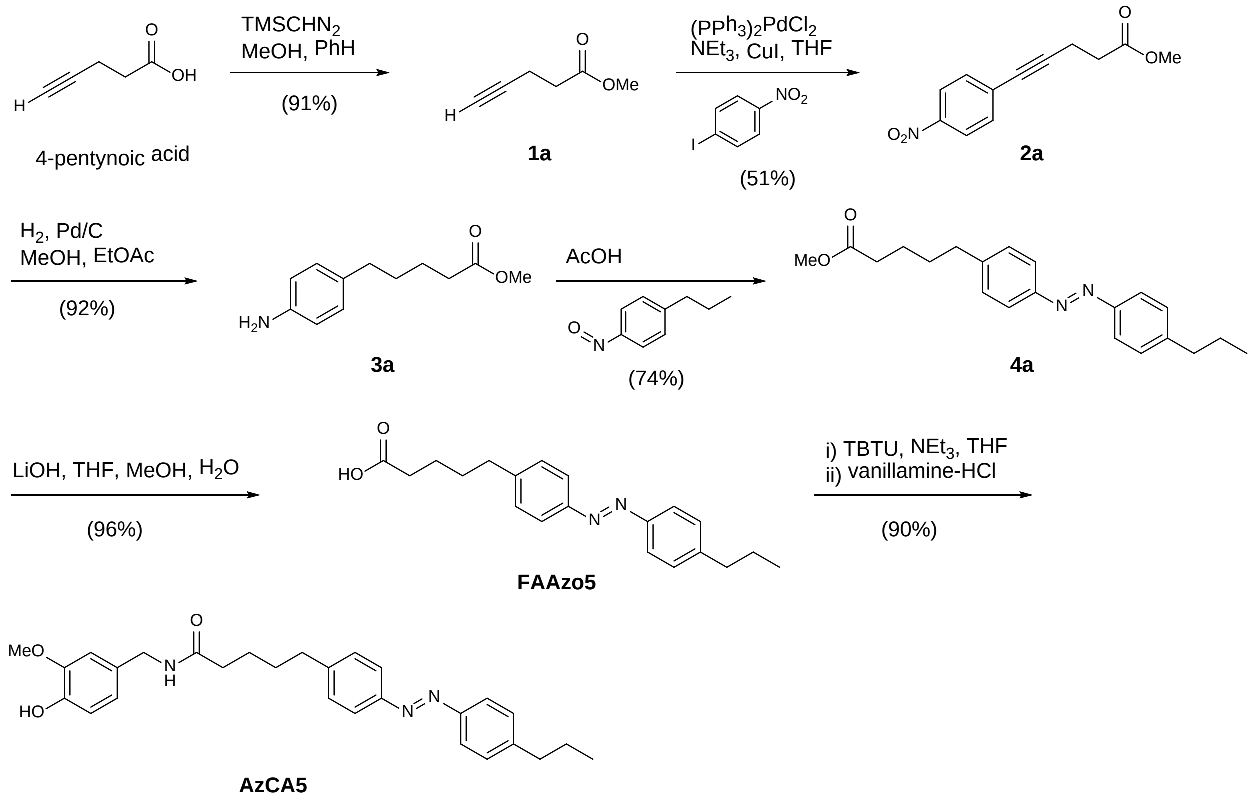

Methyl pent-4-ynoate (1a)

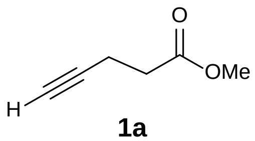

1a

Methyl pent-4-ynoate (**1a**) was prepared as reported by *García-Domínguez et al.* [1]

4-Pentynoic acid (0.36 g, 3.6 mmol, 1.0 equiv.) was dissolved in dry MeOH (7.0 mL) and benzene (25 mL) under an argon atmosphere. Trimethylsilyldiazomethane (2.4 mL, 0.20 M in diethyl ether, 4.7 mmol, 1.3 equiv.) was slowly added at room temperature until the solution retained a faint yellow color. The mixture was then titrated with AcOH until a colorless solution was obtained and the gas evolution had ceased. The reaction mixture was concentrated under reduced pressure (0.25 bar, 40 °C). The resulting oily residue was diluted with diethyl ether (50 mL) and the mixture was washed with H_2O (2x25 mL). The layers were separated and the organic layer was dried over anhydrous sodium sulfate. The dried solution was filtered, and the filtrate was concentrated under reduced pressure (0.25 bar, 40 °C) to yield **methyl pent-4-ynoate** (**1a**, 0.37 g, 91%) as a volatile colorless liquid.

TLC (hexanes/EtOAc, 4:1): Rf = 0.71. **^{1}H NMR ($CDCl_3$, 400 MHz, 25 °C):** δ 3.64 (s, 3H, H_{OMe}), 2.51-2.38 (m, 4H, $H2_{a,b}$, $H3_{a,b}$), 1.94 (t, 1H, H5, $J_{5,3}$ = 2.5 Hz). **^{13}C NMR ($CDCl_3$, 100 MHz, 25 °C):** δ 172.4 (C1), 82.4 (C4), 69.1 (C5), 51.9 (C_{OMe}), 33.1 (C2), 14.3 (C3). Spectral characteristics matched those previously reported.[1] *Note: The product still contained AcOH but this did not affect the following reaction.*

Methyl 5-(4-nitrophenyl)pent-4-ynoate (2a)

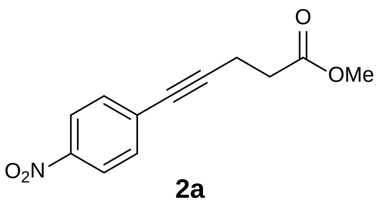

2a

Methyl 5-(4-nitrophenyl)pent-4-ynoate (2a) was prepared utilizing a modified method described by *Sugimoto et al.*[2]

Methyl pent-4-ynoate (**1a**, 0.40 g, 3.6 mmol, 1.0 equiv.) and 4-iodonitrobenzene (1.1 g, 4.3 mmol, 1.2 equiv.) were combined in a round-bottomed flask under an argon atmosphere and were dissolved in degassed/dry THF (28 mL). NEt_3 (1.5 g, 14 mmol, 4.0 equiv.) was then added, followed by the addition of CuI (0.12 g, 0.64 mmol, 0.18 equiv.) and $(PPh_3)_2PdCl_2$ (0.12 g, 0.18 mmol, 0.050 equiv.) at room temperature. The resulting brown solution was heated to 50 °C. After 2 h, the dark red mixture was cooled to room temperature and was diluted with EtOAc (50 mL), and washed with saturated aqueous ammonium chloride solution (50 mL, then 25 mL), water (25 mL) and then half saturated aqueous sodium chloride solution (25 mL). The organic phase was dried over anhydrous sodium sulfate and the dried solution was filtered. The filtrate was concentrated under reduced pressure and after being preadsorbed onto silica gel (4.0 g SiO_2, loaded from THF), the residue was purified by flash silica gel chromatography (0.10 kg SiO_2, 10:1 hexanes:EtOAc) to yield **methyl 5-(4-nitrophenyl)pent-4-ynoate** (**2a**, 0.42 g, 51%) as an orange solid.

TLC (hexanes/EtOAc, 4:1): Rf = 0.56. **^{1}H NMR ($CDCl_3$, 400 MHz, 25 °C):** δ 8.17-8.13 (m, 2H, $H8_{a,b}$, $J_{8,7}$ ≈ 9.0 Hz), 7.53-7.48 (m, 2H, $H7_{a,b}$, $J_{7,8}$ ≈ 9.0 Hz), 3.73 (s, 3H, H_{OMe}), 2.77 (t, 2H, $H2_{a,b}$, $J_{2,3}$ = 7.3 Hz), 2.65 (t, 2H, $H3_{a,b}$, $J_{3,2}$ = 7.3 Hz). **^{13}C NMR ($CDCl_3$, 100 MHz, 25 °C):** δ 172.2 (C1), 146.9 (C9), 132.5 (2C, $C7_{a,b}$), 130.7 (C6), 123.6 (2C, $C8_{a,b}$), 94.2 (C4), 79.9 (C5), 52.1 (C_{OMe}), 33.1 (C2), 15.6 (C3). **HRMS (EI$^+$):** *m/z* calcd. for [$C_{12}H_{11}NO_4$]: 233.0688, found: 233.0674 ($[M-e^-]^+$).

Methyl 5-(4-aminophenyl)pentanoate (3a)

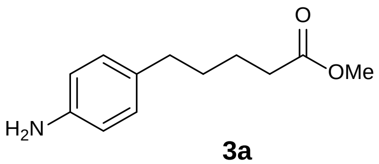

3a

Methyl 5-(4-nitrophenyl)pent-4-ynoate (**2a,** 0.42 g, 1.8 mmol, 1.0 equiv.) was dissolved in 2:1 EtOAc:MeOH (v:v, 30 mL total) and this solution was degassed by sparging with argon gas. Pd/C (10 wt%, 0.13 g) was added and the suspension was again degassed and placed under a hydrogen atmosphere (H_2 pressure applied with a balloon). The mixture was stirred vigorously at room temperature overnight. Upon completion of the reaction as observed by TLC analysis, the mixture was filtered through a pad of celite and the filtrate was concentrated under reduced pressure. The residue was purified by flash silica gel chromatography (50 g SiO_2, 3:1 hexanes:EtOAc) to yield **5-(4-aminophenyl)pentanoate** (**3a**, 0.34 g, 1.6 mmol, 92%) as an orange oil.

TLC (hexanes/EtOAc, 4:1): Rf = 0.13. **^{1}H NMR ($CDCl_3$, 400 MHz, 25 °C):** δ 6.98-6.93 (m, 2H, $H7_{a,b}$, $J_{7,8}$ ≈ 8.3 Hz), 6.64-6.60 (m, 2H, $H8_{a,b}$, $J_{8,7}$ ≈ 8.3 Hz), 3.66 (s, 3H, H_{OMe}), 3.56 (s_{br}, H_{NH2}), 2.51 (t, 2H, $H5_{a,b}$, $J_{5,4}$ = 7.2 Hz), 2.32 (t, 2H, $H2_{a,b}$), 1.69-1.54 (m, 4H, $H3_{a,b}$, $H4_{a,b}$). **^{13}C NMR ($CDCl_3$, 100 MHz, 25 °C):** δ 174.2 (C1), 144.1 (C9), 132.3 (C6), 129.1 (2C, $C7_{a,b}$), 115.3 (2C, $C8_{a,b}$), 51.5 (C_{OMe}), 34.7 (C5), 34.0 (C2), 31.2 (C4), 24.5 (C3). **HRMS (EI^+):** *m/z* calcd. for [$C_{12}H_{17}NO_2$]: 207.1259, found: 207.1238 ($[M-e^-]^+$).

Methyl 5-(4-((4-propylphenyl)diazenyl)phenyl)pentanoate (4a)

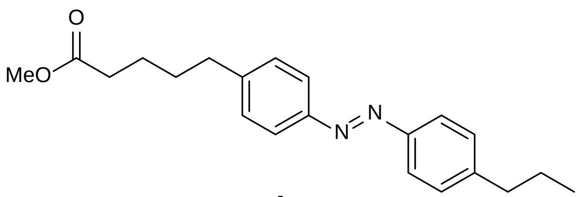

4a

A solution of 4-propylaniline (0.64 g, 4.7 mmol, 3.0 equiv.) in CH_2Cl_2 (50 mL) was treated with Oxone® (3.0 g, 9.6 mmol, 6.0 equiv.) dissolved in H_2O (50 mL) and the resulting biphasic mixture was stirred rapidly at room temperature overnight. The two phases were then separated and the organic phase was washed with H_2O (60 mL). The washed organic phase was concentrated under reduced pressure to approximately 5 mL volume and this residue was then directly purified by flash silica gel chromatography (20 g SiO_2, CH_2Cl_2). The collected green fractions were combined and a solution of **5-(4-aminophenyl)pentanoate** (**3a**, 0.33 g, 1.6 mmol, 1.0 equiv.) in CH_2Cl_2 (2.0 mL) was added followed by AcOH (25 mL). The CH_2Cl_2 was then removed under reduced pressure (0.50 bar, 40 ˚C) and the resulting solution was stirred at room temperature for 40 h. Upon complete consumption of **3a** as determined by TLC analysis, the AcOH was removed under reduced pressure and the residue was azeotroped from toluene (40 mL). The resulting brown oil was purified by flash silica gel chromatography (0.12 kg SiO_2, gradient: 20:1 to 10:1 pentane:EtOAc) to yield **methyl 5-(4-((4-propylphenyl)diazenyl)phenyl) pentanoate** (**4a**, 0.39 g, 74%) as a red oil.

TLC (hexanes/EtOAc, 20:1): Rf = 0.73. **^{1}H NMR ($CDCl_3$, 400 MHz, 25 °C):** δ 7.85-7.81 (m, 4H, $H8_{a,b}$, $H13_{a,b}$, $J_{8,7} \approx J_{13,14} \approx 8.4$ Hz), 7.33-7.29 (m, 4H, $H7_{a,b}$, $H14_{a,b}$, $J_{7,8} \approx J_{14,13} \approx 8.4$ Hz), 3.67 (s, 3H, H_{OMe}), 2.74-2.64 (m, 4H, $H5_{a,b}$, $H16_{a,b}$), 2.38-2.32 (m, 2H, $H2_{a,b}$), 1.75-1.64 (m, 6H, $H3_{a,b}$, $H4_{a,b}$, $H17_{a,b}$), 0.97 (t, 3H, $H18_{a,b,c}$, $J_{18,17} = 7.4$ Hz). **^{13}C NMR ($CDCl_3$, 100 MHz, 25 °C):** δ 174.1 (C1), 151.3 (C_{Azo}), 151.1 (C_{azo}), 146.1 (C_{azo}), 145.4 (C_{azo}), 129.3 (2C, C_{azo}), 129.2 (2C, C_{azo}), 122.9 (2C, C_{azo}), 122.8 (2C, C_{azo}), 51.7 (C_{OMe}), 38.1 (C16), 35.6 (C5), 34.0 (C2), 30.8 (C_{alk}), 24.7 (C_{alk}), 24.6 (C_{alk}), 13.9 (C18). **HRMS (EI$^+$):** *m/z* calcd. for [$C_{21}H_{26}N_2O_2$]: 338.1994, found: 338.1986 ($[M-e^-]^+$).

5-(4-((4-Propylphenyl)diazenyl)phenyl)pentanoic acid (FAAzo5)

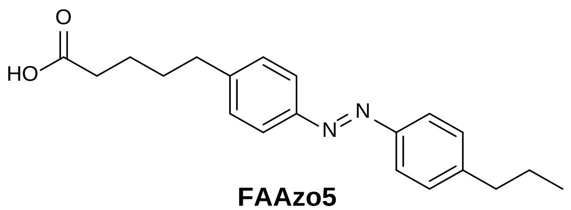

FAAzo5

Methyl 5-(4-((4-propylphenyl)diazenyl)phenyl)pentanoate (**4a**, 0.36 g, 1.1 mmol, 1.0 equiv.) was dissolved in 1:1 MeOH:THF (v:v, 42 mL total). To this solution was added aqueous 1 M lithium hydroxide solution (11 mL, 10 equiv.) and the mixture was stirred at room temperature for 2 h. Upon consumption of the starting material as observed by TLC analysis, the organic solvent was removed under reduced pressure. The resulting solution was acidified to pH = 1 with aqueous 2 M hydrochloric acid solution and the aqueous layer was extracted with EtOAc (2x30 mL). The combined organic phases were washed with H_2O (30 mL), and the washed solution was dried over anhydrous sodium sulfate. The dried solution was filtered, and the filtrate was concentrated under reduced pressure. The residue was purified by flash silica gel chromatography (32 g SiO_2, 99:1 DCM:AcOH) to yield **5-(4-((4-propylphenyl)diazenyl)phenyl) pentanoic acid** (**FAAzo5**, 0.33 g, 96%) as a red solid.

TLC (hexanes/EtOAc, 4:1): Rf = 0.18. **^{1}H NMR ($CDCl_3$, 400 MHz, 25 °C):** δ 7.85-7.81 (m, 4H, $H8_{a,b}$, $H13_{a,b}$, $J_{8,7} \approx J_{13,14} \approx$ 8.3 Hz), 7.33-7.29 (m, 4H, $H17_{a,b}$, $H14_{a,b}$, $J_{7,8} \approx J_{14,13} \approx$ 8.2 Hz), 2.75-2.63 (m, 4H, $H5_{a,b}$, $H16_{a,b}$), 2.40 (t, 2H, $H2_{a,b}$, $J_{2,3}$ = 7.0 Hz), 1.76-1.67 (m, 6H, $H3_{a,b}$, $H4_{a,b}$, $H17_{a,b}$), 0.97 (t, 3H, $H18_{a,b,c}$). **^{13}C NMR ($CDCl_3$, 100 MHz, 25 °C):** δ 179.6 (C1), 151.2 (C_{azo}), 151.0 (C_{azo}), 146.1 (C_{azo}), 141.4 (C_{azo}), 129.8 (2C, C_{azo}), 129.2 (2C, C_{azo}), 122.9 (2C, C_{azo}), 122.9 (2C, C_{azo}), 35.9 (C5), 34.0 (C2), 31.2 (C17), 29.0 (C3), 29.0 (C18), 24.7 (C4), 21.6 (C16). **IR (neat, ATR):** $\tilde{\nu}$ = 2925, 2856, 1701, 1697, 1693, 1599, 1497, 1462, 1420, 1409, 1303, 1255, 1200, 1154, 1109, 1090, 1074, 1012, 932, 851, 834, 744, 731, 677. **HRMS (EI$^+$):** *m/z* calcd. for [$C_{20}H_{24}N_2O_2$]: 324.1838, found: 324.1831 ([M-e$^-$]$^+$).

***N*-(4-Hydroxy-3-methoxybenzyl)-5-(4-((4-propylphenyl)diazenyl)phenyl)pentanamide (AzCA5)**

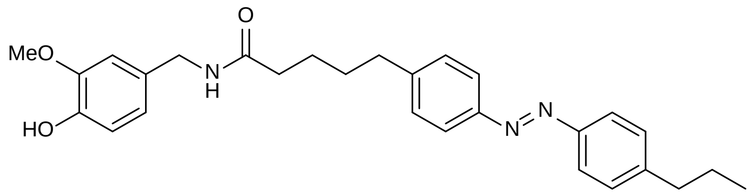

AzCA5

5-(4-((4-Propylphenyl)diazenyl)phenyl)pentanoic acid (**FAAzo5**, 21 mg, 65 µmol, 1 equiv.) was converted to red solid ***N*-(4-hydroxy-3-methoxybenzyl)-5-(4-((4-propylphenyl)diazenyl) phenyl)pentanamide** (**AzCA5**, 27 mg, 90%) in an analogous manner as described above for the preparation of ***N*-(4-hydroxy-3-methoxybenzyl)-4-((4-heptylphenyl)diazenyl)benzamide (AzCA1)**. *Note: all reagents and solvents were scaled according to molarity.*

TLC (CH_2Cl_2/AcOH, 99:1): Rf = 0.43. **^{1}H NMR ($CDCl_3$, 400 MHz, 25 °C):** δ 7.84-7.79 (m, 4H, $H8_{a,b}$, $H13_{a,b}$, $J_{8,7} \approx J_{13,14} \approx$ 8.4 Hz), 7.33-7.24 (m, 4H, $H7_{a,b}$, $H14_{a,b}$, $J_{7,8} \approx J_{14,13} \approx$ 8.4 Hz), 6.85 (d, 1H, H24, $J_{24,25}$ = 8.0 Hz), 6.77-6.71 (m, 2H, H21, H25), 5.80 (t, 1H, NH, $J_{NH,19}$ = 5.8 Hz), 4.33 (d, 2H, $H19_{a,b}$, $J_{19,NH}$ = 5.7 Hz), 3.79 (s, 3H, $H26_{a,b,c}$), 2.71-2.63 (m, 4H, $H5_{a,b}$, $H16_{a,b}$), 2.22 (t, 2H, $H2_{a,b}$, $J_{2,3}$ = 7.0 Hz), 1.77-1.63 (m, 6H, $H3_{a,b}$, $H4_{a,b}$, $H17_{a,b}$), 0.97 (t, 3H, $H18_{a,b,c}$, $J_{18,17}$ = 7.3 Hz). **^{13}C NMR ($CDCl_3$, 100 MHz, 25 °C):** δ 172.7 (C1), 151.2 (C_{azo}), 151.1 (C_{azo}), 146.8 (C22), 146.2 (C_{azo}), 145.5 (C_{azo}), 145.2 (C23), 130.3 (C20), 129.2 (2C, C_{azo}), 129.1 (2C, C_{azo}), 122.9 (2C, C_{azo}), 122.8 (2C, C_{azo}), 120.9 (C25), 114.5 (C24), 110.8 (C21), 55.9 (C26), 43.6 (C19), 38.0 (C16), 36.7 (C2), 35.7 (C5), 31.0 (C_{alk}), 25.5 (C_{alk}), 24.5 (C17), 13.9 (C18). **IR (neat, ATR): $\tilde{\nu}$** = 3291, 3054, 2956, 2930, 2859, 1644, 1600, 1515, 1463, 1430, 1376, 1274, 1236, 1155, 1124, 1035, 1013, 849, 736. **HRMS (EI^+):** *m/z* calcd. for [$C_{28}H_{33}N_3O_3$]: 459.2522, found: 459.2507 ($[M-e^-]^+$).

Preparation of *N*-(4-hydroxy-3-methoxybenzyl)-6-(4-((4-ethylphenyl)diazenyl)phenyl) hexanamide (AzCA6)

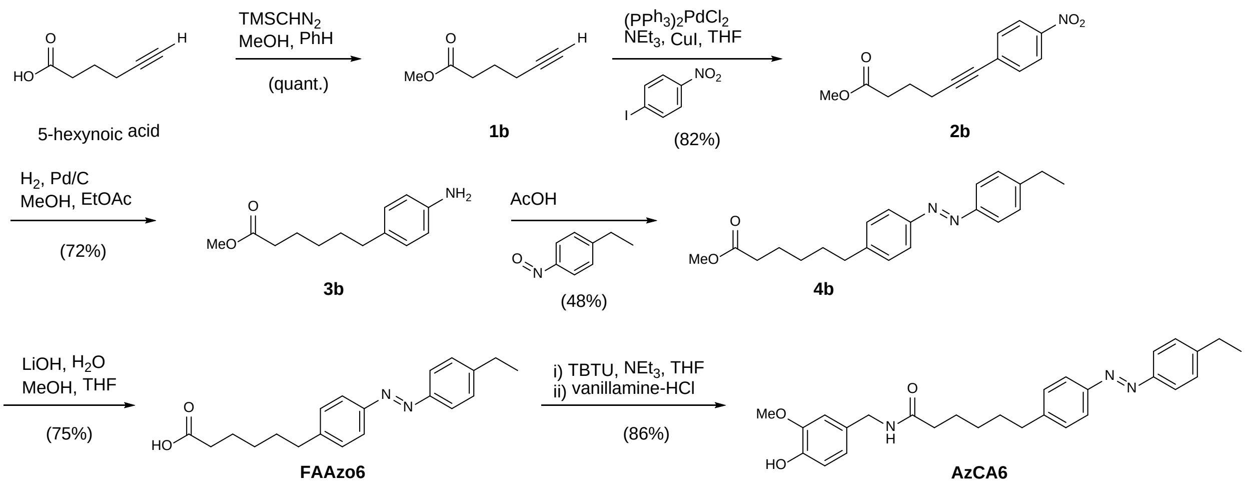

Methyl hex-5-ynoate (1b)

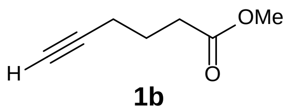

1b

5-Hexynoic acid (0.10 g, 0.89 mmol, 1.0 equiv.) was converted to **methyl hex-5-ynoate** (**1b**, 0.12 g, quant.) in an analogous manner as described above for the preparation of **methyl pent-4-ynoate (1a).** *Note: all reagents and solvents were scaled according to molarity.*

TLC (hexanes/EtOAc, 4:1): Rf = 0.73. **^{1}H NMR ($CDCl_3$, 400 MHz, 25 °C):** δ 3.67 (s, 3H, H_{OMe}), 2.45 (t, 2H, $H2_{a,b}$, $J_{2,3}$ = 7.4 Hz), 2.25 (dt, 2H, $H4_{a,b}$, $J_{4,3}$ = 6.7 Hz, $J_{4,6}$ = 2.7 Hz), 1.96 (t, 1H, H6, $J_{6,4}$ = 2.7 Hz), 1.84 (quint, 2H, $H3_{a,b}$, $J_{3,2} \approx J_{3,4} \approx$ 7.4 Hz). **^{13}C NMR ($CDCl_3$, 100 MHz, 25 °C):** δ 173.7 (C1), 83.4 (C5), 69.2 (C6), 51.7 (C_{OMe}), 32.8 (C2), 23.7 (C3), 18.0 (C4). Spectral characteristics matched those previously reported[3]. *Note: The product still contained AcOH but this did not affect the following reaction.*

Methyl 6-(4-nitrophenyl)hex-5-ynoate (2b)

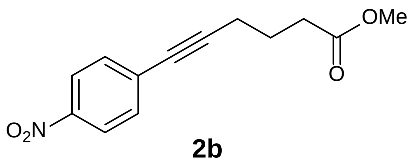

2b

Methyl hex-5-ynoate (**1b**, 94 mg, 0.75 mmol, 1.0 equiv.) was converted to **methyl 6-(4-nitrophenyl)hex-5-ynoate** (**2b**, 0.15 g, 0.61 mmol, 82%) in an analogous manner as described above for the preparation of **methyl 5-(4-nitrophenyl)pent-4-ynoate (2a).** *Note: all reagents and solvents were scaled according to molarity.*

TLC (hexanes/EtOAc, 4:1): Rf = 0.46. **^{1}H NMR ($CDCl_3$, 400 MHz, 25 °C):** δ 8.18-8.14 (m, 2H, $H9_{a,b}$, $J_{9,8} \approx 9.0$ Hz), 7.53-7.49 (m, 2H, $H8_{a,b}$, $J_{8,9} \approx 9.0$ Hz), 3.69 (s, 3H, H_{OMe}), 2.56-2.49 (m, 4H, $H2_{a,b}$, $H4_{a,b}$), 1.96 (quint, 2H, $H3_{a,b}$, $J_{3,2} = J_{3,4} \approx 7.2$ Hz). **^{13}C NMR ($CDCl_3$, 100 MHz, 25 °C):** δ 173.5 (C1), 146.9 (C10), 132.4 (2C, $C8_{a,b}$), 130.9 (C7), 123.7 (2C, $C9_{a,b}$), 95.2 (C5), 80.2 (C6), 51.8 (C_{OMe}), 33.0 (C2), 23.7 (C3), 19.2 (C4).

Methyl 6-(4-aminophenyl)hexanoate (3b)

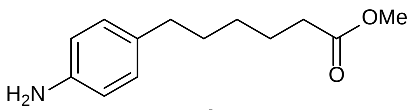

3b

Methyl 6-(4-nitrophenyl)hex-5-ynoate (**2b,** 0.12 g, 0.5 mmol, 1 equiv.) was converted to **methyl 6-(4-aminophenyl)hexanoate** (**3b**, 79 mg, 0.36 mmol, 72%) in an analogous manner as described above for the preparation of **methyl 5-(4-aminophenyl)pentanoate (3a).** *Note: all reagents and solvents were scaled according to molarity.*

TLC (hexanes/EtOAc, 4:1): Rf = 0.13. **^{1}H NMR ($CDCl_3$, 400 MHz, 25 °C):** δ 6.98-6.93 (m, 2H, $H8_{a,b}$, $J_{8,9}$ ≈ 8.5 Hz), 6.64-6.60 (dt, 2H, $H9_{a,b}$, $J_{9,8}$ ≈ 8.4 Hz), 3.66 (s, 3H, H_{OMe}), 3.55 (s_{br}, 2H, H_{NH2}), 2.50 (t, 2H, $H6_{a,b}$, $J_{6,5}$ = 7.6 Hz), 2.30 (t, 2H, $H2_{a,b}$, $J_{2,3}$ = 7.5 Hz), 1.69-1.52 (m, 4H, $H3_{a,b}$, $H5_{a,b}$), 1.38-1.28 (m, 2H, $H4_{a,b}$). **^{13}C NMR ($CDCl_3$, 100 MHz, 25 °C):** δ 174.4 (C1), 144.2 (C10), 132.7 (C7), 129.2 (2C, $C8_{a,b}$), 115.3 (2C, $C9_{a,b}$), 51.6 (C_{OMe}), 34.9 (C6), 34.2 (C2), 31.5 (C5), 28.8 (C3), 25.0 (C4). **HRMS (EI^+):** *m/z* calcd. for [$C_{13}H_{19}NO_2$]: 221.1416, found: 221.1408 ($[M\text{-}e^-]^+$).

Methyl 6-(4-((4-ethylphenyl)diazenyl)phenyl)hexanoate (4b)

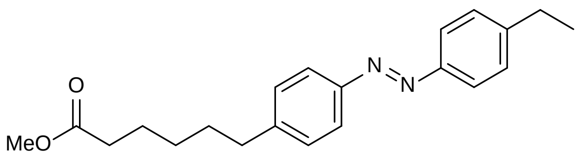

4b

4-Ethylaniline (0.12 g, 0.95 mmol, 3.0 equiv.) and **methyl 6-(4-aminophenyl)hexanoate** (**3b**, 70 mg, 0.32 mmol, 1.0 equiv.) were converted to orange oil **methyl 6-(4-((4-ethylphenyl) diazenyl)phenyl)hexanoate** (**4b**, 52 mg, 48%) in an analogous manner as described above for the preparation of **methyl 5-(4-((4-propylphenyl)diazenyl)phenyl) pentanoate (4a).** *Note: all reagents and solvents were scaled according to molarity.*

TLC (pentane/EtOAc, 20:1): Rf = 0.24 (*trans*), 0.10 (*cis*). **^{1}H NMR ($CDCl_3$, 400 MHz, 25 °C):** δ 7.85-7.80 (m, 4H, $H9_{a,b}$, $H14_{a,b}$, $J_{9,8} \approx J_{14,15} \approx$ 8.4 Hz), 7.35-7.28 (m, 4H, $H8_{a,b}$, $H15_{a,b}$, $J_{8,9} \approx J_{15,14} \approx$ 8.5 Hz), 3.67 (s, 3H, H_{OMe}), 2.77-2.65 (m, 4H, $H6_{a,b}$, $H17_{a,b}$), 2.32 (t, 2H, $H2_{a,b}$), 1.73-1.62 (m, 4H, $H3_{a,b}$, $H5_{a,b}$), 1.43-1.34 (m, 2H, $H4_{a,b}$), 1.29 (t, 3H, $H18_{a,b,c}$, $J_{18,17}$ = 7.6 Hz). **^{13}C NMR ($CDCl_3$, 100 MHz, 25 °C):** δ 174.3 (C1), 151.2 (C_{azo}), 151.1 (C_{azo}), 147.6 (C_{azo}), 145.9 (C_{azo}), 129.2 (2C, C_{azo}), 128.7 (2C, C_{azo}), 122.9 (2C, C_{azo}), 122.9 (2C, C_{azo}), 51.7 (C_{OMe}), 35.7 (C_{alk}), 34.1 (C2), 31.1 (C_{alk}), 29.0 (C_{alk}), 28.9 (C_{alk}), 24.9 (C_{alk}), 15.6 (C18). **HRMS (EI$^+$):** *m/z* calcd. for [$C_{21}H_{26}N_2O_2$]: 338.1994, found: 338.1983 ([M-e$^-$]$^+$).

6-(4-((4-Ethylphenyl)diazenyl)phenyl)hexanoic acid (FAAzo6)

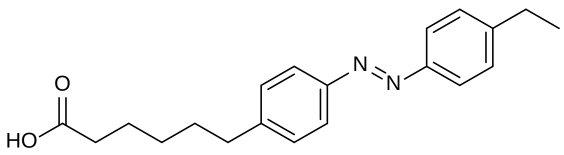

FAAzo6

Methyl 6-(4-((4-ethylphenyl)diazenyl)phenyl)hexanoate (**4b**, 45 mg, 0.13 mmol, 1.0 equiv.) was converted to red solid **6-(4-((4-ethylphenyl)diazenyl)phenyl)hexanoic acid** (**FAAzo6**, 32 mg, 75%) in an analogous manner as described above for the preparation of **5-(4-((4-propylphenyl)diazenyl)phenyl)pentanoic acid (FAAzo5).** *Note: all reagents and solvents were scaled according to molarity.*

TLC (CH_2Cl_2:AcOH, 99:1): Rf = 0.22. **^{1}H NMR ($CDCl_3$, 400 MHz, 25 °C):** δ 7.85-7.80 (m, 4H, $H9_{a,b}$, $H14_{a,b}$, $J_{9,10} \approx J_{14,15} \approx$ 8.3 Hz), 7.35-7.28 (m, 4H, $H8_{a,b}$, $H15_{a,b}$, $J_{8,9} \approx J_{15,14} \approx$ 8.4 Hz), 2.77-2.65 (m, 4H, $H6_{a,b}$, $H17_{a,b}$), 2.39-2.33 (t, 2H, $H2_{a,b}$, $J_{2,3}$ = 7.4 Hz), 1.73-1.64 (m, 4H, $H3_{a,b}$, $H5_{a,b}$), 1.46-1.37 (m, 2H, $H4_{a,b}$), 1.29 (t, 3H, $H18_{a,b,c}$, $J_{18,17}$ = 7.5 Hz). **^{13}C NMR ($CDCl_3$, 100 MHz, 25 °C):** δ 179.4 (C1), 151.2 (C_{azo}), 151.1 (C_{azo}), 147.7 (C_{azo}), 145.8 (C_{azo}), 129.2 (2C, C_{azo}), 128.7 (2C, C_{azo}), 122.9 (2C, C_{azo}), 122.9 (2C, C_{azo}), 35.7 (C_{alk}), 34.0 (C2), 31.0 (C_{alk}), 29.0 (C_{alk}), 28.8 (C_{alk}), 24.6 (C_{alk}), 15.6 (C18). **IR (neat, ATR): $\tilde{\nu}$** = 3022, 2956, 2927, 2851, 1692, 1600, 1497, 1466, 1438, 1410, 1355, 1293, 1249, 1200, 1152, 1056, 1010, 913, 848, 826, 729, 685. **HRMS (EI$^+$):** *m/z* calcd. for [$C_{20}H_{24}N_2O_2$]: 324.1838, found: 324.1838 ([M-e$^-$]$^+$).

_N_-(4-Hydroxy-3-methoxybenzyl)-6-(4-((4-ethylphenyl)diazenyl)phenyl)hexanamide (AzCA6)

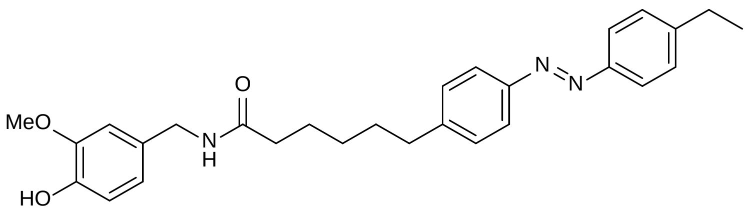

AzCA6

6-(4-((4-Ethylphenyl)diazenyl)phenyl)hexanoic acid (**FAAzo6**, 16 mg, 49 µmol, 1 equiv.) was converted to red solid ***N*-(4-hydroxy-3-methoxybenzyl)-6-(4-((4-ethylphenyl)diazenyl) phenyl)hexanamide** (**AzCA6**, 19 mg, 86%) in an analogous manner as described above for the preparation of ***N*-(4-hydroxy-3-methoxybenzyl)-4-((4-heptylphenyl)diazenyl)benzamide (AzCA1)**. *Note: all reagents and solvents were scaled according to molarity.*

TLC (hexanes/EtOAc, 1:2): Rf = 0.31. **^{1}H NMR ($CDCl_3$, 400 MHz, 25 °C):** δ 7.85-7.79 (m, 4H, $H9_{a,b}$, $H14_{a,b}$, $J_{9,8} \approx J_{14,15} \approx 8.3$ Hz), 7.35-7.25 (m, 4H, $H8_{a,b}$, $H15_{a,b}$, $J_{8,9} \approx J_{15,14} \approx 8.3$ Hz), 6.86 (d, 1H, H24, $J_{24,25} = 8.1$ Hz), 6.81-6.72 (m, 2H, H21, H25), 5.82-5.66 (m, 2H, NH, OH, $J_{NH,19} = 5.3$ Hz), 4.33 (d, 2H, $H19_{a,b}$, $J_{19,NH} = 5.7$ Hz), 3.84 (s, 3H, $H26_{a,b,c}$), 2.77-2.63 (m, 4H, $H6_{a,b}$, $H17_{a,b}$), 2.19 (t, 2H, $H2_{a,b}$, $J_{2,3} = 7.5$ Hz), 1.74-1.62 (m, 4H, $H5_{a,b}$, $H3_{a,b}$), 1.42-1.32 (m, 2H, $H4_{a,b}$), 1.28 (t, 3H, $H18_{a,b,c}$, $J_{18,17} = 7.8$ Hz). **^{13}C NMR ($CDCl_3$, 100 MHz, 25 °C):** δ 172.8 (C1), 151.2 (C_{azo}), 151.1 (C_{azo}), 147.7 (C_{azo}), 146.8 (C22), 145.8 (C_{azo}), 145.2 (C23), 130.4 (C20), 129.2 (2C, C_{azo}), 128.6 (2C, C_{azo}), 122.9 (2C, C_{azo}), 122.9 (2C, C_{azo}), 120.9 (C25), 114.5 (C24), 110.8 (C21), 56.0 (C26), 43.7 (C19), 36.8 (C2), 35.7 (C_{alk}), 31.1 (C_{alk}), 29.0 (C_{alk}), 28.9 (C_{alk}), 25.7 (C_{alk}), 15.6 (C_{alk}). **IR (neat, ATR):** $\tilde{\nu}$ = 3291, 3053, 2961, 2930, 2856, 1651, 1645, 1600, 1557, 1539, 1515, 1546, 1530, 1373, 1274, 1237, 1155, 1124, 1035, 1013, 845, 795, 736. **HRMS (EI$^+$):** *m/z* calcd. for [$C_{28}H_{33}N_3O_3$]: 459.2522, found: 459.2520 ([M-e$^-$]$^+$).

Preparation of *N*-(4-hydroxy-3-methoxybenzyl)-7-(4-(p-tolyldiazenyl)phenyl) heptanamide (AzCA7)

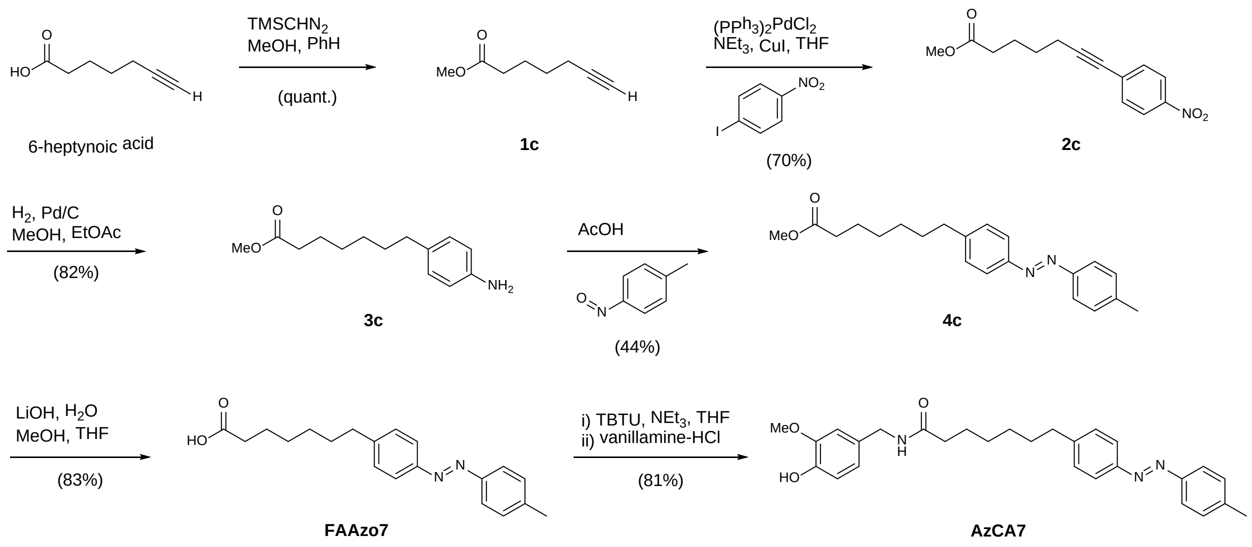

Methyl hept-6-ynoate (1c)

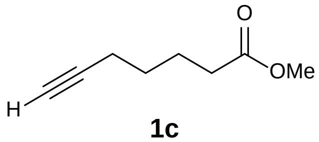

1c

6-Heptynoic acid (0.10 g, 0.79 mmol, 1.0 equiv.) was converted to **methyl hept-6-ynoate** (**1c**, 0.11 g, quant.) in an analogous manner as described above for the preparation of **methyl pent-4-ynoate (1a).** *Note: all reagents and solvents were scaled according to molarity.*

TLC (hexanes/EtOAc, 4:1): Rf = 0.71. **^{1}H NMR ($CDCl_3$, 400 MHz, 25 °C):** δ 3.65 (s, 3H, H_{OMe}), 2.32 (t, 2H, $H2_{a,b}$, $J_{2,3}$ = 7.4 Hz), 2.19 (dt, 2H, $H5_{a,b}$, $J_{5,4}$ = 7.1 Hz, $J_{5,7}$ = 2.6 Hz), 1.94 (t, 1H, H7, $J_{7,5}$ = 2.5 Hz), 1.78-1.69 (m, 2H, H_{alk}), 1.59-1.50 (m, 2H, H_{alk}). *Note: The product still contained AcOH but this did not affect the next reaction.*

Methyl 7-(4-nitrophenyl)hept-6-ynoate (2c)

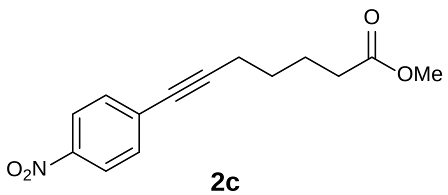

2c

Methyl hept-6-ynoate (**1c**, 96 mg, 0.69 mmol, 1.0 equiv.) was converted to **methyl 7-(4-nitrophenyl)hept-6-ynoate** (**2c**, 0.12 g, 0.47 mmol, 70%) in an analogous manner as described above for the preparation of **methyl 5-(4-nitrophenyl)pent-4-ynoate (2a).** *Note: all reagents and solvents were scaled according to molarity.*

TLC (hexanes/EtOAc, 4:1): Rf = 0.53. **^{1}H NMR ($CDCl_3$, 400 MHz, 25 °C):** δ 8.17-8.13 (m, 2H, $H10_{a,b}$, $J_{10,9}$ ≈ 8.9 Hz), 7.53-7.49 (m, 2H, $H9_{a,b}$, $J_{9,10}$ ≈ 8.9 Hz), 3.68 (s, 3H, H_{OMe}), 2.48 (t, 2H, $H2_{a,b}$, $J_{2,3}$ = 7.0 Hz), 2.38 (t, 2H, $H5a_{a,b}$, $J_{5,4}$ = 7.3 Hz), 1.85-1.76 (m, 2H, $H4_{a,b}$), 1.70-1.62 (m, 2H, $H3_{a,b}$). **^{13}C NMR ($CDCl_3$, 100 MHz, 25 °C):** δ 173.9 (C1), 146.8 (C11), 132.4 (2C, $C9_{a,b}$), 131.1 (C8), 123.6 (2C, $C10_{a,b}$), 95.9 (C6), 79.8 (C7), 51.8 (C_{OMe}), 33.6 (C2), 27.9 (C3), 24.3 (C4), 19.5 (C5). **HRMS (EI$^+$):** *m/z* calcd. for [$C_{15}H_{17}N_1O_4$]: 275.1158, found: 275.1134 ([M-e$^-$]$^+$).

Methyl 7-(4-aminophenyl)heptanoate (3c)

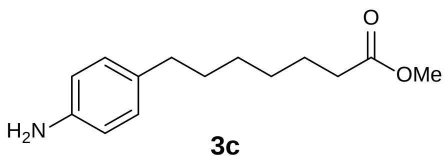

3c

Methyl 7-(4-nitrophenyl)hept-6-ynoate (**2c,** 0.12 g, 0.4 mmol, 1 equiv.) was converted to orange oil **methyl 7-(4-aminophenyl)heptanoate** (**3c**, 85 mg, 0.36 mmol, 82%) in an analogous manner as described above for the preparation of **methyl 5-(4-aminophenyl)pentanoate (3a).** *Note: all reagents and solvents were scaled according to molarity.*

TLC (hexanes/EtOAc, 4:1): Rf = 0.12. **^{1}H NMR ($CDCl_3$, 400 MHz, 25 °C):** δ 6.99-6.93 (m, 2H, $H9_{a,b}$, $H_{9,10}$ ≈ 8.2 Hz), 6.65-6.60 (m, 2H, $H10_{a,b}$, $J_{10,9}$ ≈ 8.3 Hz), 3.66 (s, 3H, H_{OMe}), 3.55 (s_{br}, H_{NH2}), 2.49 (t, 2H, $H7_{a,b}$, $J_{7,6}$ = 8.1 Hz), 2.30 (t, 2H, $H2_{a,b}$), 1.66-1.50 (m, 4H, $H3_{a,b}$, $H6_{a,b}$), 1.39-1.28 (m, 4H, $H4_{a,b}$, $H5_{a,b}$). **^{13}C NMR ($CDCl_3$, 100 MHz, 25 °C):** δ 174.4 (C1), 144.2 (C11), 132.9 (C8), 129.2 (2C, $C9_{a,b}$), 115.3 (2C, $C10_{a,b}$), 51.6 (C_{OMe}), 35.1 (C7), 34.2 (C2), 31.7 (C_{alk}), 29.1 (C_{alk}), 28.9 (C_{alk}), 25.0 (C_{alk}). **HRMS (ESI$^+$):** *m/z* calcd. for $[C_{15}H_{24}NO_2]^+$: 250.1807, found: 250.1801 ($[M+H]^+$).

Methyl 7-(4-(p-tolyldiazenyl)phenyl)heptanoate (4c)

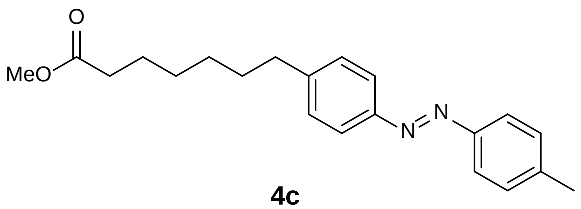

4c

p-Toluidine (0.10 g, 0.96 mmol, 3.0 equiv.) and **methyl 7-(4-aminophenyl)heptanoate** (**3c**, 70 mg, 0.32 mmol, 1.0 equiv.) were converted to orange oil **methyl 7-(4-(p-tolyldiazenyl)phenyl)heptanoate** (**4c**, 48 mg, 44%) in an analogous manner as described above for the preparation of **methyl 5-(4-((4-propylphenyl)diazenyl)phenyl)pentanoate (4a).** *Note: all reagents and solvents were scaled according to molarity.*

TLC (hexanes/EtOAc, 4:1): Rf = 0.74 (*trans*), 0.55 (*cis*). **^{1}H NMR (CDCl$_3$, 400 MHz, 25 °C):** δ 7.84-7.79 (m, 4H, $H10_{a,b}$, $H15_{a,b}$), 7.33-7.28 (m, 4H, $H9_{a,b}$, $H16_{a,b}$, $J_{9,10} \approx J_{16,15} \approx$ 8.5 Hz), 3.67 (s, 3H, H_{OMe}), 2.68 (t, 2H, $H7_{a,b}$, $J_{7,6}$ = 7.5 Hz), 2.43 (s, 3H, $H18_{a,b}$), 2.32-2.27 (m, 2H, $H2_{a,b}$), 1.71-1.54 (m, 4H, $H3_{a,b}$, $H6_{a,b}$), 1.41-1.28 (m, 4H, $H4_{a,b}$, $H5_{a,b}$). **^{13}C NMR (CDCl$_3$, 100 MHz, 25 °C):** δ 174.4 (C1), 151.1 (C_{azo}), 151.0 (C_{azo}), 146.1 (C_{azo}), 141.4 (C_{azo}), 129.8 (2C, C_{azo}), 129.2 (2C, C_{azo}), 122.9 (2C, C_{azo}), 122.9 (2C, C_{azo}), 51.6 (C_{OMe}), 35.9 (C7), 34.2 (C2), 31.2 (C_{alk}), 29.1 (C_{alk}), 29.0 (C_{alk}), 25.0 (C_{alk}), 21.6 (C18). **HRMS (EI$^+$):** *m/z* calcd. for [$C_{21}H_{26}N_2O_2$]: 338.1994, found: 338.1991 ([M-e$^-$]$^+$).

7-(4-(*p*-Tolyldiazenyl)phenyl)heptanoic acid (FAAzo7)

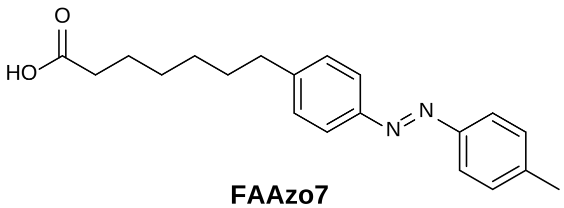

FAAzo7

Methyl 7-(4-(*p*-tolyldiazenyl)phenyl)heptanoate (**4c**, 35 mg, 0.10 mmol, 1 equiv.) was converted to red solid **7-(4-(*p*-tolyldiazenyl)phenyl)heptanoic acid** (**FAAzo7**, 28 mg, 83%) in an analogous manner as described above for the preparation of **5-(4-((4-propylphenyl) diazenyl)phenyl)pentanoic acid (FAAzo5).** *Note: all reagents and solvents were scaled according to molarity.*

TLC (hexanes/EtOAc, 4:1): Rf = 0.13. **^{1}H NMR ($CDCl_3$, 400 MHz, 25 °C):** δ 7.84-7.79 (m, 4H, $H10_{a,b}$, $H15_{a,b}$), 7.32-7.28 (m, 4H, $H9_{a,b}$, $H16_{a,b}$, $J_{9,10} \approx J_{16,15} \approx 8.3$ Hz), 2.68 (t, 2H, $H7_{a,b}$, $J_{7,6} = 7.6$ Hz), 2.43 (s, 3H, $H18_{a,b}$), 2.35 (t, 2H, $H2_{a,b}$, $J_{2,3} = 7.5$ Hz), 1.71-1.58 (m, 4H, $H3_{a,b}$, $H6_{a,b}$), 1.42-1.31 (m, 4H, $H4_{a,b}$, $H5_{a,b}$). **^{13}C NMR ($CDCl_3$, 100 MHz, 25 °C):** δ 179.6 (C1), 151.2 (C_{azo}), 151.0 (C_{azo}), 146.1 (C_{azo}), 141.4 (C_{azo}), 129.8 (2C, C_{azo}), 129.2 (2C, C_{azo}), 122.9 (2C, C_{azo}), 122.9 (2C, C_{azo}), 35.9 (C7), 34.0 (C2), 31.2 (C_{alk}), 29.0 (C_{alk}), 29.0 (C_{alk}), 24.7 (C_{alk}), 21.6 (C18). **IR (neat, ATR):** $\tilde{\nu}$ = 2927, 2851, 1720, 1702, 1697, 1693, 1600, 1497, 1465, 1438, 1413, 1314, 1295, 1280, 1241, 1208, 1152, 1109, 1010, 954, 850, 826, 727. **HRMS (ESI^-):** *m/z* calcd. for $[C_{20}H_{23}N_2O_2]^-$: 323.1765, found: 323.1762 ($[M-H]^-$).

N-(4-Hydroxy-3-methoxybenzyl)-7-(4-(p-tolyldiazenyl)phenyl)heptanamide (AzCA7)

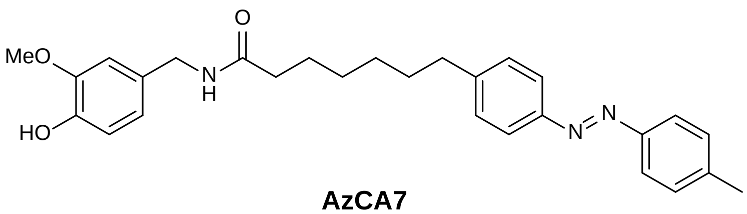

AzCA7

7-(4-(*p*-Tolyldiazenyl)phenyl)heptanoic acid (**FAAzo7**, 14 mg, 43 µmol, 1.0 equiv.) was converted to red solid ***N*-(4-hydroxy-3-methoxybenzyl)-7-(4-(*p*-tolyldiazenyl)phenyl) heptanamide** (**AzCA7**, 16 mg, 81%) in an analogous manner as described above for the preparation of ***N*-(4-hydroxy-3-methoxybenzyl)-4-((4-heptylphenyl)diazenyl)benzamide (AzCA1)**. *Note: all reagents and solvents were scaled according to molarity.*

TLC (hexanes/EtOAc, 2:1): Rf = 0.31. **^{1}H NMR ($CDCl_3$, 400 MHz, 25 °C):** δ 7.76-7.71 (m, 4H, $H10_{a,b}$, $H15_{a,b}$, $J_{10,9} \approx J_{15,16} \approx 8.4$ Hz), 7.25-7.19 (m, 4H, $H9_{a,b}$, $H16_{a,b}$, $J_{9,10} \approx J_{16,15} \approx 8.3$ Hz), 6.78 (d, 1H, H24, $J_{24,25}$ = 8.0 Hz), 6.73-6.65 (m, 2H, H21, H25), 5.71-5.56 (m, 2H, NH, OH, $J_{NH,19}$ = 5.3 Hz), 4.26 (d, 2H, H19 $J_{19,NH}$ = 5.4 Hz), 3.77 (s, 3H, $H26_{a,b,c}$), 2.58 (t, 2H, $H7_{a,b}$, $J_{7,6}$ = 7.5 Hz), 2.36 (s, 3H, $H18_{a,b,c}$), 2.10 (t, 2H, $H2_{a,b}$, $J_{2,3}$ = 7.4 Hz), 1.62-1.51 (m, 4H, $H3_{a,b}$, $H6_{a,b}$), 1.32-1.24 (m, 4H, $H4_{a,b}$, $H5_{a,b}$). **^{13}C NMR ($CDCl_3$, 100 MHz, 25 °C):** δ 172.8 (C1), 151.0 (C_{azo}), 150.9 (C_{azo}), 146.8 (C22), 145.9 (C_{azo}), 145.1 (C23), 141.3 (C_{azo}), 130.3 (C20), 129.7 (2C, C_{azo}), 129.1 (2C, C_{Azo}), 122.7 (4C, C_{Azo}), 120.8 (C25), 114.4 (C24), 110.7 (C21), 55.9 (C26), 43.5 (C19), 36.8 (C2), 35.8 (C7), 31.1 (C_{alk}), 29.1 (C_{alk}), 28.9 (C_{alk}), 25.7 (C_{alk}), 21.5 (C18). **IR (neat, ATR):** $\tilde{\nu}$ = 3292, 2927, 2854, 1652, 1645, 1600, 1558, 1539, 1515, 1464, 1456, 1429, 1373, 1275, 1237, 1155, 1124, 1035, 823, 727. **HRMS (EI$^+$):** *m/z* calcd. for [$C_{28}H_{33}N_3O_3$]: 459.2522, found: 459.2524 ([M-e$^-$]$^+$).

Preparation of *N*-(4-hydroxy-3-methoxybenzyl)-8-(4-(phenyldiazenyl)phenyl) octanamide (AzCA8)

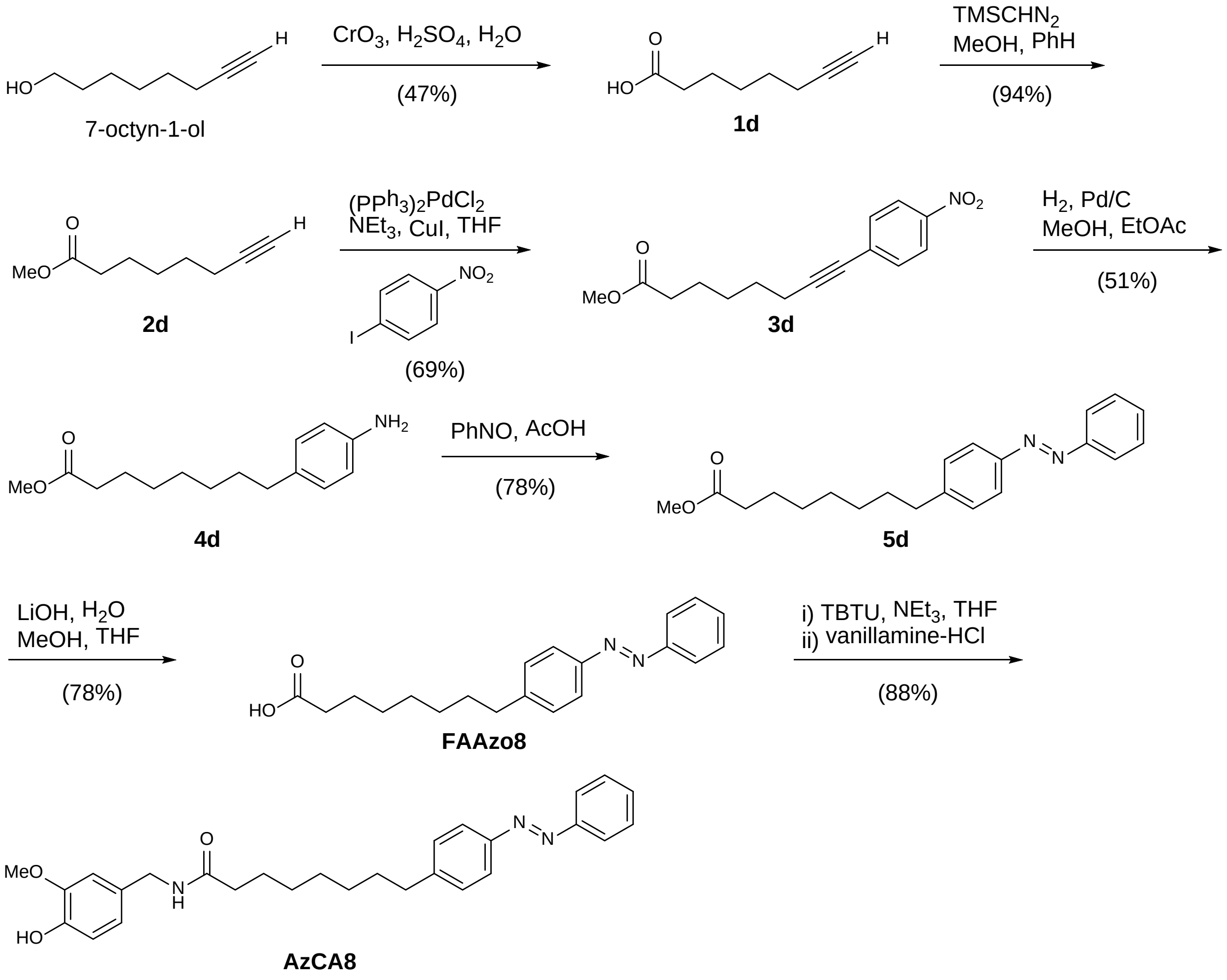

Oct-7-ynoic acid (1d)

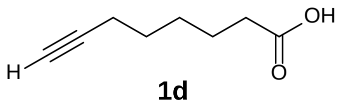

1d

Oct-7-ynoic acid (**1d**) was prepared from 7-octyn-1-ol according to a modified procedure reported by *Reyes et al*[4].

CrO_3 (0.78 g, 7.8 mmol, 1.5 equiv.) was dissolved in H_2O (10 mL) and cooled to 0 °C. H_2SO_4 (96%, 2.4 mL, 45 mmol, 8.7 equiv.) was slowly added and the mixture was allowed to warm to room temperature over 10 min. The solution was again cooled to 0 °C whereupon **7-octyn-1-ol** (0.65 g, 5.2 mmol, 1.0 equiv.) dissolved in acetone (3.3 mL) was added slowly. Upon complete addition of the alcohol, the reaction mixture was allowed to warm to room temperature and stirring was continued for 2 h. The reaction mixture was then diluted with EtOAc (40 mL) and the phases were separated. The aqueous phase was extracted with EtOAc (40 mL) and the combined organic phases were concentrated. The resulting oil was purified by flash column chromatography (65 g SiO_2, 4:1 hexanes:EtOAc to 3:1 hexanes:EtOAc) to yield **oct-7-ynoic acid** (**1d**, 0.34 g, 47%) as a clear, volatile liquid. *Note: This product still contained CH_2Cl_2 which was not further removed due to its volatile nature. This did not affect the following reaction.*

TLC (hexanes/EtOAc, 4:1): Rf = 0.16. **^{1}H NMR ($CDCl_3$, 400 MHz, 25 °C):** δ 10.23 (s_{br}, 1H, H_{COOH}), 2.36 (t, 2H, $H2_{a,b}$, $J_{2,3}$ = 7.6 Hz), 2.19 (dt, 2H, $H6_{a,b}$, $J_{6,5}$ = 6.9 Hz, $J_{6,8}$ = 2.8 Hz), 1.98 (t, 1H, H8, $J_{8,6}$ = 2.8 Hz), 1.69-1.59 (m, 2H, H_{alk}), 1.58-1.50 (m, 2H, H_{alk}), 1.50-1.40 (m, 2H, $H4_{a,b}$). **^{13}C NMR ($CDCl_3$, 100 MHz, 25 °C):** δ 180.2 (C1), 84.4 (C5), 68.5 (C6), 34.0 (C2), 28.2 (C_{alk}), 28.2 (C_{alk}), 24.3 (C_{alk}), 18.3 (C_{alk}). **HRMS (EI^+):** *m/z* calcd. for $[C_8H_{12}O_2]^+$: 140.0837, found: 140.0764 ($[M-e^-]^+$).

Methyl oct-7-ynoate (2d)

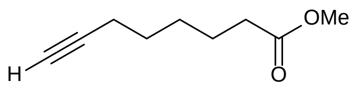

2d

7-Octynoic acid (**1d**, 0.34 g, 2.4 mmol, 1.0 equiv.) was converted to **methyl oct-7-ynoate** (**2d**, 0.35 g, 94%) in an analogous manner as described above for the preparation of **methyl pent-4-ynoate (1a).** *Note: all reagents and solvents were scaled according to molarity.*

TLC (hexanes/EtOAc, 4:1): Rf = 0.50. **^{1}H NMR ($CDCl_3$, 400 MHz, 27 °C):** δ 3.65 (s, 3H, H_{OMe}), 2.31 (t, 2H, $H2_{a,b}$, $J_{2,3}$ = 7.5 Hz), 2.18 (dt, 2H, $H6_{a,b}$, $J_{6,5}$ = 7.0 Hz, $J_{6,8}$ = 2.7 Hz), 1.93 (t, 1H, H8, $J_{8,6}$ = 2.7 Hz), 1.67-1.58 (m, 2H, H_{alk}), 1.56-1.49 (m, 2H, H_{alk}), 1.47-1.39 (m, 2H, $H4_{a,b}$). **^{13}C NMR ($CDCl_3$, 400 MHz, 27 °C):** δ 174.2 (C1), 84.4 (C5), 68.4 (C6), 51.6 (C_{OMe}), 34.0 (C2), 28.3 (C_{alk}), 28.2 (C_{alk}), 24.5 (C_{alk}), 18.3 (C_{alk}).

Methyl 8-(4-nitrophenyl)oct-7-ynoate (3d)

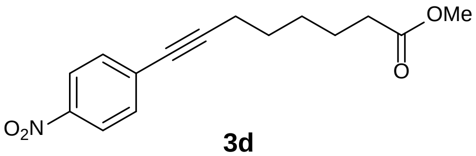

3d

Methyl oct-7-ynoate (**2d**, 350 mg, 2.30 mmol, 1.0 equiv.) was converted to **methyl 8-(4-nitrophenyl)oct-7-ynoate** (**3d**, 430 mg, 69%) in an analogous manner as described above for the preparation of **methyl 5-(4-nitrophenyl)pent-4-ynoate (2a).** *Note: all reagents and solvents were scaled according to molarity.*

TLC (hexanes/EtOAc, 4:1): Rf = 0.59. **^{1}H NMR ($CDCl_3$, 400 MHz, 25 °C):** δ 8.13 (d, 2H, $H11_{a,b}$, $J_{11,10}$ = 8.8 Hz), 7.49 (d, 2H, $H10_{a,b}$, $J_{10,11}$ = 8.8 Hz), 3.65 (s, 3H, H_{OMe}), 2.44 (t, 2H, $H2_{a,b}$, $J_{2,3}$ = 6.9 Hz), 2.33 (t, 2H, $H6_{a,b}$, $J_{6,5}$ = 7.4 Hz), 1.72-1.58 (m, 4H, $H3_{a,b}$, $H5_{a,b}$), 1.53-1.43 (m, 2H, $H4_{a,b}$). **^{13}C NMR ($CDCl_3$, 100 MHz, 25 °C):** δ 174.1 (C1), 146.7 (C12), 132.3 (2C, $C10_{a,b}$), 131.2 (C9), 123.6 (2C, $C11_{a,b}$), 96.4 (C7), 79.6 (C8), 51.6 (C_{OMe}), 34.0 (C2), 28.5 (C_{alk}), 28.1 (C_{alk}), 24.5 (C_{alk}), 19.5 (C6). **HRMS (EI^+):** *m/z* calcd. for $[C_{15}H_{17}NO_4]^+$: 275.1158, found: 275.1134 ($[M-e^-]^+$).

Methyl 8-(4-aminophenyl)octanoate (4d)

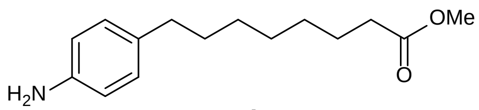

4d

Methyl 8-(4-nitrophenyl)oct-7-ynoate (**3d**, 43 mg, 0.16 mmol, 1 equiv.) was converted to orange oil **methyl 8-(4-aminophenyl)octanoate** (**4d**, 20 mg, 51%) in an analogous manner as described above for the preparation of **methyl 5-(4-aminophenyl)pentanoate (3a).** *Note: all reagents and solvents were scaled according to molarity.*

TLC (hexanes/EtOAc, 4:1): Rf = 0.19. **^{1}H NMR ($CDCl_3$, 400 MHz, 25 °C):** δ 6.97-6.95 (m, 2H, $H10_{a,b}$, $J_{10,11}$ ≈ 8.5 Hz), 6.64-6.60 (m, 2H, $H11_{a,b}$, $J_{11,10}$ ≈ 8.5 Hz), 3.66 (s, 3H, H_{OMe}), 3.55 (s_{br}, 2H, H_{NH2}), 2.48 (t, 2H, $H8_{a,b}$, $J_{8,7}$ = 7.4 Hz), 2.29 (t, 2H, $H2_{a,b}$, $J_{2,3}$ = 7.5 Hz), 1.65-1.49 (m, 4H, $H3_{a,b}$, $H7_{a,b}$), 1.36-1.22 (m, 6H, $H4_{a,b}$, $H5_{a,b}$, $H6_{a,b}$). **^{13}C NMR ($CDCl_3$, 100 MHz, 25 °C):** δ 174.5 (C1), 144.1 (C12), 133.1 (C9), 129.2 (2C, $C10_{a,b}$), 115.3 (2C, $C11_{a,b}$), 51.6 (C_{OMe}), 35.2 (C8), 34.2 (C2), 31.9 (C_{alk}), 29.3 (C_{alk}), 29.2 (C_{alk}), 29.2 (C_{alk}), 25.1 (C_{alk}). **HRMS (ESI^{+}):** *m/z* calcd. for $[C_{14}H_{24}NO_2]^{+}$: 250.1807, found: 250.1801 ($[M+H]^{+}$).

Methyl 8-(4-(phenyldiazenyl)phenyl)octanoate (5d)

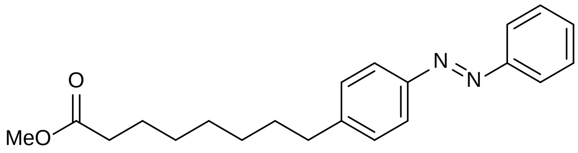

5d

Methyl 8-(4-aminophenyl)octanoate (**4d**, 15 mg, 60 µmol, 1.0 equiv.) was dissolved in CH_2Cl_2 (1 mL). Nitrosobenzene (8 mg, 72 µmol, 1.2 equiv.) followed by AcOH (36 mg, 0.60 mmol, 10 equiv.) were added to this solution and the resulting mixture was stirred at room temperature for 7 h. Upon completion of the reaction, the mixture was diluted with EtOAc (5 mL), and the organic phase was washed with a saturated aqueous sodium bicarbonate solution (10 mL) and water (2x10 mL). The layers were separated, and the organic phase was dried over anhydrous sodium sulfate. The dried solution was filtered and the filtrate was then concentrated under reduced pressure. The residue was purified by flash column chromatography (5 g SiO_2, 20:1 pentane:EtOAc) to yield **methyl 8-(4-(phenyldiazenyl)phenyl)octanoate** (**5d**, 16 mg, 78%) as an orange oil.

TLC (hexanes/EtOAc, 20:1): Rf = 0.36 (*trans*), 0.10 (*cis*). **^{1}H NMR ($CDCl_3$, 400 MHz, 25 °C):** δ 7.92-7.88 (m, 2H, $H11_{a,b,}$ $J_{11,10}$ ≈ 8.4 Hz), 7.87-7.82 (m, 2H, $H16_{a,b}$, $J_{16,17}$ ≈ 8.4 Hz), 7.54-7.43 (m, 3H, $H17_{a,b}$, H18), 7.32 (d, 2H, $H10_{a,b}$, $J_{10,11}$ = 8.4 Hz), 3.67 (s, 3H, H_{OMe}), 2.68 (t, 2H, $H8_{a,b}$, $J_{8,7}$ = 7.4 Hz), 2.30 (t, 2H, $H2_{a,b}$, $J_{2,3}$ = 7.5 Hz), 1.70-1.50 (m, 4H, $H3_{a,b}$, $H7_{a,b}$), 1.41-1.25 (m, 6H, $H4_{a,b}$, $H5_{a,b}$, $H6_{a,b}$).. **^{13}C NMR ($CDCl_3$, 100 MHz, 25 °C):** δ 174.4 (C1), 152.9 (C_{azo}), 151.1 (C_{azo}), 146.6 (C9), 130.8 (C18), 129.2 (2C, C_{azo}), 129.2 (2C, C_{azo}), 123.0 (2C, C_{azo}), 122.9 (2C, C_{azo}), 51.6 (C_{OMe}), 36.0 (C8), 34.2 (C2), 31.4 (C_{alk}), 29.2 (C_{alk}), 29.2 (C_{alk}), 29.2 (C_{alk}), 25.1 (C_{alk}). **HRMS (ESI$^+$):** *m/z* calcd. for $[C_{21}H_{27}N_2O_2]^+$: 339.2073, found: 339.2065 ($[M+H]^+$).

8-(4-(Phenyldiazenyl)phenyl)octanoic acid (FAAzo8)

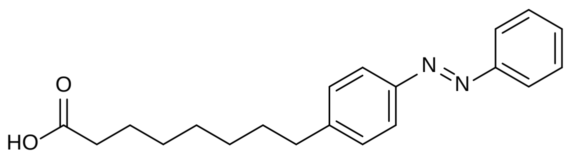

FAAzo8

Methyl 8-(4-(phenyldiazenyl)phenyl)octanoate (**5d**, 16 mg, 47 µmol, 1 equiv.) was converted to red solid **8-(4-(phenyldiazenyl)phenyl)octanoic acid** (**FAAzo8**, 12 mg, 78%) in an analogous manner as described above for the preparation of **5-(4-((4-propylphenyl)diazenyl) phenyl)pentanoic acid (FAAzo5).** *Note: all reagents and solvents were scaled according to molarity.*

TLC (hexanes/EtOAc, 4:1): Rf = 0.13. **^{1}H NMR ($CDCl_3$, 400 MHz, 25 °C):** δ 7.92-7.88 (m, 2H, $H11_{a,b}$, $J_{11,10}$ ≈ 8.5 Hz), 7.87-7.82 (m, 2H, $H16_{a,b,}$ $J_{16,17}$ ≈ 8.5 Hz), 7.54-7.43 (m, 3H, $H17_{a,b}$, H18), 7.32 (d, 2H, $H10_{a,b}$, $J_{10,11}$ = 8.4 Hz), 2.68 (t, 2H, $H8_{a,b}$, $J_{8,7}$ = 7.5 Hz), 2.35 (t, 2H, $H2_{a,b}$, $J_{2,3}$ = 7.4 Hz), 1.70-1.59 (m, 4H, $H3_{a,b}$, $H7_{a,b}$), 1.41-1.28 (m, 6H, $H4_{a,b}$, $H5_{a,b}$, $H6_{a,b}$). **^{13}C NMR ($CDCl_3$, 100 MHz, 25 °C):** δ 179.9 (C1), 152.9 (C_{azo}), 151.1 (C_{azo}), 146.5 (C9), 130.8 (C18), 129.2 (2C, C_{azo}), 129.2 (2C, C_{azo}), 123.0 (2C, C_{azo}), 122.8 (2C, C_{azo}), 36.0 (C8), 34.1 (C2), 31.3 (C_{alk}), 29.2 (C_{alk}), 29.2 (C_{alk}), 29.1 (C_{alk}), 24.8 (C_{alk}). **IR (neat, ATR):** ***ṽ*** = 3180, 2921, 2848, 1701, 1602, 1486, 1464, 1428, 1409, 1343, 1303, 1289, 1264, 1226, 1198, 1154, 1088, 1012, 925, 840, 806, 767, 759, 725, 685. **HRMS (ESI$^-$):** *m/z* calcd. for $[C_{20}H_{23}N_2O_2]^-$: 323.1765, found: 323.1762 ($[M-H]^-$).

***N*-(4-Hydroxy-3-methoxybenzyl)-8-(4-(phenyldiazenyl)phenyl)octanamide (AzCA8)**

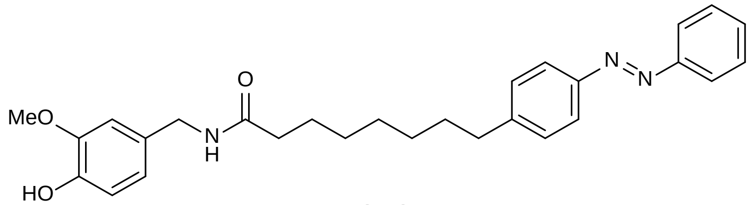

AzCA8

8-(4-(phenyldiazenyl)phenyl)octanoic acid (**FAAzo8**, 12 mg, 37 µmol, 1.0 equiv.) was converted to red oil ***N*-(4-hydroxy-3-methoxybenzyl)-8-(4-(phenyldiazenyl)phenyl) octanamide** (**AzCA8**, 15 mg, 88%) in an analogous manner as described above for the preparation of ***N*-(4-hydroxy-3-methoxybenzyl)-4-((4-heptylphenyl)diazenyl)benzamide (AzCA1)**. *Note: all reagents and solvents were scaled according to molarity.*

TLC (hexanes/EtOAc, 1:2): Rf = 0.26. **^{1}H NMR ($CDCl_3$, 400 MHz, 25 °C):** δ 7.92-7.88 (m, 2H, $H11_{a,b}$), 7.86-7.82 (m, 2H, $H16_{a,b}$, $J_{16,17} \approx 8.4$ Hz), 7.54-7.43 (m, 3H, $H17_{a,b}$, H18), 7.31 (d, 2H, $H10_{a,b}$, $J_{10,11} = 8.4$ Hz), 6.88-6.72 (m, 3H, H21, H24, H25), 5.77-5.63 (m, 2H, H_{NH}, H_{OH}), 4.34 (d, 2H, $H19_{a,b}$, $J_{19,NH} = 5.6$ Hz), 3.85 (s, 3H, H_{OMe}), 2.67 (t, 2H, $H8_{a,b}$, $J_{8,7} = 7.6$ Hz), 2.18 (t, 2H, $H2_{a,b}$, $J_{2,3} = 7.5$ Hz), 1.70-1.58 (m, 4H, $H3_{a,b}$, $H6_{a,b}$), 1.38-1.22 (m, 6H, $H4_{a,b}$, $H5_{a,b}$, $H6_{a,b}$). **^{13}C NMR ($CDCl_3$, 100 MHz, 25 °C):** δ 173.0 (C1), 152.8 (C_{azo}), 151.1 (C_{azo}), 146.8 (C22), 146.5 (C9), 145.2 (C23), 130.8 (C18), 130.5 (C20), 129.2 (2C, C_{azo}), 129.2 (2C, C_{azo}), 123.0 (2C, C_{azo}), 122.8 (2C, C_{azo}), 120.9 (C25), 114.5 (C24), 110.8 (C20), 56.0 (C_{OMe}), 43.7 (C19), 36.9 (C2), 36.0 (C8), 31.3 (C_{alk}), 29.3 (C_{alk}), 29.2 (2C, C_{alk}), 25.9 (C_{alk}). **IR (neat, ATR): $\tilde{\nu}$** = 3286, 2928, 2853, 1733, 1717, 1699, 1683, 1674, 1652, 1645, 1634, 1616, 1600, 1557, 1538, 1516, 1464, 1456, 1436, 1418, 1373, 1274, 1153, 1124, 1035, 768, 688, 667. **HRMS (EI$^+$):** *m/z* calcd. for $[C_{28}H_{33}N_3O_3]^+$: 459.2522, found: 459.2506 ($[M\text{-}e^-]^+$).

Supplementary References

1. García-Domínguez, P. *et al.* A DNA methyltransferase modulator inspired by peyssonenyne natural product structures. *ChemMedChem* **7,** 2101–2112 (2012).
2. Sugimoto, K. *et al.* Protecting-group-free total synthesis of (-)-rhazinilam and (-)-rhazinicine using a gold-catalyzed cascade cyclization. *Angew. Chem. Int. Ed. Engl.* **52,** 7168–7171 (2013).
3. Papahatjis, D. P., Nahmias, V. R., Nikas, S. P., Schimpgen, M. & Makriyannis, A. Design and synthesis of (13S)-methyl-substituted arachidonic acid analogues: templates for novel endocannabinoids. *Chemistry* **16,** 4091–4099 (2010).
4. Reyes, S., Huigens, R. W., Su, Z., Simon, M. L. & Melander, C. Synthesis and biological activity of 2-aminoimidazole triazoles accessed by Suzuki-Miyaura cross-coupling. *Org. Biomol. Chem.* **9,** 3041–3049 (2011).
